# Betaine alleviates obesity-related metabolic disorders in rats: insights from microbiomes, lipidomics, and transcriptomics

**DOI:** 10.3389/fnut.2025.1604801

**Published:** 2025-07-10

**Authors:** Xian Wang, Xin Shang, Yan Pan, Yu Fu, Huilian Zhu, Shuxun Yan

**Affiliations:** ^1^Department of Endocrinology, The First Affiliated Hospital of Henan University of Chinese Medicine, Zhengzhou, China; ^2^First School of Clinical Medicine, Henan University of Chinese Medicine, Zhengzhou, China; ^3^Guangdong Provincial Key Laboratory of Food, Nutrition and Health, Department of Nutrition, School of Public Health, Sun Yat-Sen University, Guangzhou, China

**Keywords:** betaine, obesity, gut microbiota, lipidomics, transcriptomics

## Abstract

**Background:**

Betaine is a natural food component that plays an important role in improving body composition, but the details of its effects on adipose tissue remains to be elucidated. Here, we hypothesize that betaine can alleviate obesity-related metabolic disorders, thus gut microbiota, transcriptomics and lipidomics were used to investigate the obesity-alleviating effects of betaine on high-fat diet (HFD)-fed rats.

**Methods:**

In this study, rats fed a HFD were subjected to an 8-week oral intervention with betaine. We measured changes in body weight (BW), blood lipid profiles, and gastrointestinal hormones to assess therapeutic effects. The epididymal white adipose tissue was extracted and stained to evaluate the pathological morphology of adipocytes. The structure of the rat gut microbiota and the expression profiles of lipid metabolism-related genes in adipocytes were analyzed using 16S rRNA sequencing and transcriptomics. Furthermore, the lipid metabolomics of adipose tissue samples was investigated using an LC-MS analysis platform.

**Results:**

Our findings imply that betaine has been linked to the alleviation of community alterations in gut microbiota resulting from HFD, facilitating the proliferation of beneficial microbiota (e.g., Lactobacillu, Lachnospiraceae_NK4A136_group) and the generation of short-chain fatty acids (SCFA). Particularly, our results indicate that betaine treatment led to notable variations in the content of DHA-riched glycerophospholipids which exhibited a positive correlation with the secretion of intestinal hormone (e.g., cholecystokininand peptide YY). Additionally, betaine upregulated the expression of genes related to thermogenesis and glycerophospholipids metabolic pathways in the adipose tissues.

**Conclusion:**

Our findings indicate that betaine has the potential to decrease HFD-related obesity by regulating adipose tissue metabolism and promote the synthesis of polyunsaturated phospholipid rich in DHA. The underlying mechanism of betaine action might encompass its influence on gut microbiota modulation and SCFA metabolism.

## 1 Introduction

Obesity is a driver of many chronic diseases and has become a common global challenge (1). The World Obesity Atlas published in 2024 predicts that the global prevalence of overweight and obesity among adults is expected to increase from 2.2 billion in 2020 to 3.3 billion by 2035, with the worldwide proportion increasing from 42 to 54%. This means that almost one in every five adults is overweight or obese. Obesity is defined as an abnormal or excessive buildup of body fat that can negatively impact health and is strongly linked to disorders affecting multiple organs and systems, including renal failure, diabetes mellitus, liver disease, cardiovascular disease, osteoarthritis, and dyslipidemia (2–6). Furthermore, Obesity has become the largest contributor of the metabolic disease burden and is associated with 13 different forms of cancer (7, 8). Currently, food restriction and exercise are the main treatments for obesity, but these are difficult to accomplish in the fast-paced, modern world. Therefore, more therapy targets must be identified in order to provide safer and more efficient obesity treatments.

Obesity and overweight have many different causes, including environmental, dietary, and hereditary factors (9). Gut is one of the largest interfaces between the internal environment and the outside world, and gut microbiota is involved in many physiological processes such as digestion, metabolism or energy absorption of the host (10–12). Changes in gut microbiota have also been linked to the development of obesity (13), with recent evidence revealing that quantitative and qualitative differences exist in the gut microbiota between lean and obese individuals (14).

Betaine, also known as N-trimethylglycine, is typically encountered in a variety of foods, including shellfish, wheat, beetroots, and spinach (15). For the past few years, betaine has been widely studied in the fields of functional food and biomedicine because of its beneficial biological activities (16–19). Originating from dietary intake as well as the oxidation of choline, betaine plays a role as a methyl donor in the maintenance of osmotic pressure balance and participates in normal lipid metabolism in animals (20, 21). Betaine deficiency is associated with many obesity-related metabolic diseases, including lipid disorders, diabetes, and vascular diseases. In recent years, the role of betaine to combat obesity in animals, including rats (22), pigs (23), and chickens (24) has been confirmed. Furthermore, research involving humans has indicated that higher betaine concentration is related to lower BMI, body fat percentage (BFP) and waist circumference (25). Nevertheless, the specifics regarding the impact of this substance on obesity, adipose tissue, as well as its fundamental molecular mechanisms, are still largely unknown.

To date, no studies have combined microbiota analysis, transcriptomics and lipidomics to investigate the effectiveness of betaine and elucidate its potential underlying mechanism by which it regulates lipid metabolism in adipose tissue. Our earlier research has demonstrated that the serum level of betaine exhibited a dose-dependent correlation with improved body composition and fat distribution patterns, attributed to a reduction in trunk fat mass (26). In this study, dual-energy X-ray absorptiometry (DXA) was employed to assess serum betaine levels, along with body composition and fat distribution, with the aim of determining the association and investigating the impact of betaine on lipid accumulation. The above results could offer a foundational rationale for the utilization of betaine in functional food products and it encouraged us to further explore the application of betaine in obesity prevention and control.

Therefore, to investigate the impact of betaine on adipose tissue metabolism and diet-induced obesity, our study performed multi-omics analyses including gut microbiota sequencing, transcriptomics, and lipidomics. Through comprehensive analysis of betaine’s effects on molecular interaction networks in HFD-fed rats, our integrated multi-omics approach provides unique systemic insights for mechanistic research and the development of food-derived therapeutics.

## 2 Materials and methods

### 2.1 Animals and diets

The 6-week-old male SD rats (Vitalriver laboratory animal technology, Beijing, China) were randomly divided into 3 groups after 1 week of adaptive feeding: (I) normal chow diet group (NCD; 70% carbohydrate, 20% protein, 10% fat by energy); (II) HFD-fed group (HFD; 35% carbohydrate, 20% protein, 45% fat by energy); (III) HFD with 1.5% betaine-treated HFD-fed group (HBE). After fed with HFD for 16 weeks, group (III) were changed to add 1.5% betaine administration in drinking water (13328B, Adamas-beta®, Shanghai, China) once daily for another 8 weeks, while continuing to consume the HFD. At the end of experiment, all rats’ epididymal white adipose tissues (eWATs) were collected and stored at −80°C. Animal experimental ethics review committee of Henan TCM University granted approval for the animal experiments (approval number: IACUC-202307016).

### 2.2 Physiological and biochemical analyses

Blood samples were obtained from fasted rats at 4°C for serum separation, which involved centrifugation at 3000 g for 15 min at the same temperature. The serum was subsequently stored at −20°C. Servicebio Biotechnology Co., Ltd. (Wuhan, China) conducted the measurements of serum liver transhepatic enzyme levels and lipid profile indicators. The concentrations of triglycerides (TG) in eWATs were quantified with a triglyceride assay kit (Rayto life and analytical sciences Co., Ltd., Shenzhen, China). SCFAs content in feces was analyzed using gas chromatography–mass spectrometry (8890B, Agilent, United States). Toward the end of the experiment, after rats that had fasted for the whole night received an intraperitoneal injection of 2 g glucose/kg body weight, the blood glucose level measured from the tail vein was determined after 0, 30, 60, 90, and 120 min of injection with the glucometer (Roche Diagnostics, Mannheim, Germany), respectively.

### 2.3 Hematoxylin–eosin staining

After fixation with 10% neutral formalin, epididymal white adipose tissue (eWAT) and liver tissue were embedded in paraffin and subsequently sectioned into 5-μm-thick slices. These slices were then stained using hematoxylin and eosin (H&E) and examined under the upright microscope (Eclipse, Niokon, Japan).

### 2.4 Gut microbiota analysis

Genomic DNA was promptly extracted from fresh stool samples employing a fecal DNA isolation kit (MJYH, Shanghai, China), adhering to the manufacturer’s instructions, and subsequently stored at −80°C. PCR amplification cycling conditions were set as follows: initial denaturation at 95°C for 3 min, followed by 27 cycles of 95°C for 30 s (denaturation), 55°C for 30 s (annealing), and 72°C for 45 s (extension). A final extension at 72°C lasted for 10 min, ending at 4°C. Subsequently, high-throughput pyrosequencing of the PCR products was performed on an Illumina NextSeq 2000 PE300 platform (Illumina, San Diego, United States). Using the OTUs data, Mothur v1.30.2 was employed to compute rarefaction curves as well as alpha diversity metrics, such as the Chao and Ace indices. Principal coordinate analysis (PCoA) was conducted to assess the similarity between microbial communities across various samples and the linear discriminant analysis effect size (LEfSe) was utilized to identify bacterial taxa with significant abundance, defined by an LDA score > 3.5 and a *p*-value < 0.05.

### 2.5 Untargeted adipose tissue metabolome analysis

LC–MS/MS analysis was conducted on a Thermo UHPLC-Q Exactive HF-X Vanquish Horizon system equipped with an Accucore C30 column (100 mm × 2.1 mm i.d., 2.6 μm; Thermo, United States). Mass spectrometric data was acquired employing a Benchtop Orbitrap Mass Spectrometer (UHPLC-Q-Exactive HF-X, Thermo, USA) which was furnished with a heated-electrospray ionization (HESI) source capable of operating in both positive and negative ion modes. The data matrix obtained from the preprocessing results encompassed information on lipid class, retention time (RT), mass-to-charge ratio (m/z) values, along with the corresponding peak intensities.

### 2.6 RNA-sequencing analysis

Total RNA was extracted from six individual adipose tissue samples of each group. The transcripts per million reads (TPM) method was employed to calculate the expression level of each transcript and identify the differential expression genes (DEGs). RSEM was used to quantify gene abundances (27). Essentially, the DESeq2 package was utilized to conduct the differential expression analysis. Genes were deemed significantly differentially expressed if they exhibited a |log2FC| ≥ 1 and a *p*-value < 0.05, according to DESeq2 criteria. Furthermore, functional-enrichment analysis was conducted to annotate DEGs that were significantly enriched in metabolic pathways, with a p-value threshold of <0.05, relative to the whole-transcriptome background.

### 2.7 Statistical analysis

All animal experimental data were expressed as mean ± SD. Before performing statistical analyses, the normality of the data was assessed using the Shapiro–Wilk test. GraphPad Prism 9.5 (GraphPad Software, San Diego, United States) was utilized to plot histograms and line graphs. For data that were normally distributed, multiple comparisons were performed by one-way ANOVA with Tukey’s post-hoc test. For data that did not meet the assumption of normality, the Kruskal-Wallis one-way analysis of variance by ranks was applied. *p* < 0.05 compared between two groups were considered statistically significant. Using iPath 3.0, a comprehensive transcriptome and metabolome analysis was performed to identify significant alterations in metabolic pathways. Bioinformatics analyses were performed on the online platform.^1^

## 3 Results

### 3.1 Betaine alleviates obesity-related metabolic disorders resulting from HFD

**Figure 1:**
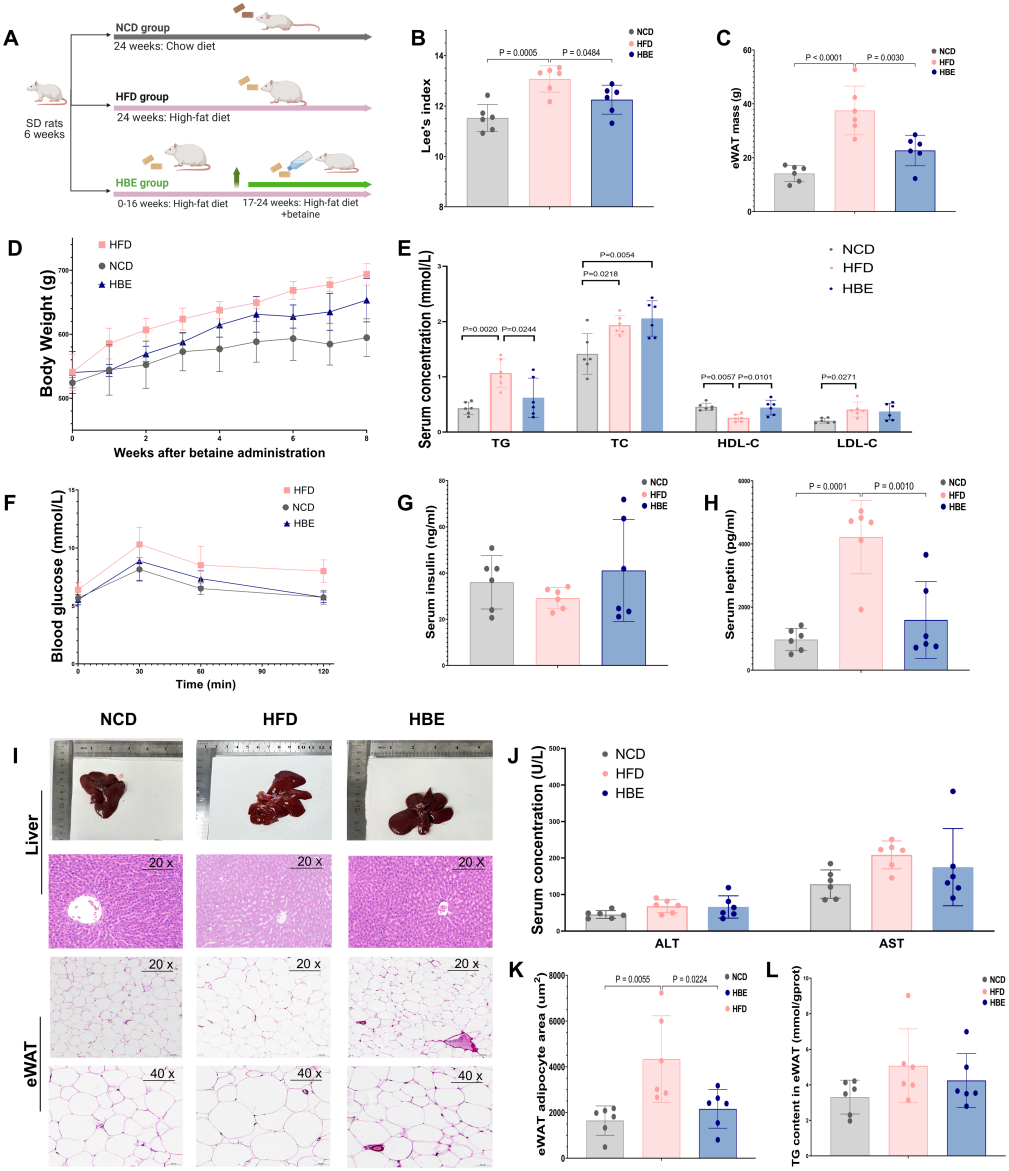
Effects of betaine on body weight, tissue mass, and histopathology of adipocytes in HFD-fed rats. **(A)** A cartoon representing the betaine administration intervention in rats fed an HFD; The **(B)** lee’s index, **(C)** epididymal white adipose tissue (eWAT) mass weight of each group; **(E)** TC, TG, LDL-C, HDL-C levels, **(G)** insulin and **(H)** leptin concentrations in serum of each group; **(D)** body weight in HFD-fed rats over the 8-weeks intervention of betaine; **(F)** glucose concentrations during an glucose tolerance test (GTT); **(I)** representative H&E staining, three rats for each group were used for H&E staining with similar results; **(J)** the alanine transaminase (ALT) and aspartate aminotransferase (AST) levels in serum; **(K)** the TG levels in eWAT; **(K)** the mean adipocyte area of eWAT. Data were expressed as mean ± SD (*n* = 6).

After administering 1.5% betaine in the drinking water and continuing HFD feeding for a duration of 8 weeks (Figure 1A), Lee’s index and the weight of epididymal white adipose tissue (eWAT) were lower in the HBE group than in the HFD group (Figures 1B,C). The lipid profile findings indicated that the levels of TG in the HBE group were markedly decreased compared to those observed in the HFD group, while high-density lipoprotein cholesterol (HDL-C) levels were increased (Figure 1E). Glucose tolerance tests (GTT) showed that the HBE group exhibited higher glucose tolerance compared to HFD rats (Figure 1F). However, no statistically significant differences were observed in fasting insulin levels (Figure 1G), alanine aminotransferase (ALT), or aspartate aminotransferase (AST) between the two groups (Figure 1J).

In addition, another characteristic of diet-induced obesity is hyperleptinemia, and mutations in leptin and leptin receptors can in turn lead to obesity (28). We found that betaine intervention significantly reduced serum leptin levels and body weight gain (Figures 1D,H) in HFD-fed rats when HFD was continuously administered, suggesting that the anti-obesity effect of betaine may be related to leptin signaling and carbohydrate metabolism.

Histological examination with H&E staining (Figure 1I) showed a decreased adipocyte area of the adipose tissue in the HBE group compared to the HFD group (Figure 1K). Together, the current findings collectively highlight the beneficial role of betaine in alleviating obesity-related metabolic disorders resulting from HFD.

### 3.2 Betaine regulates the structure and composition of gut microbiota

**Figure 2:**
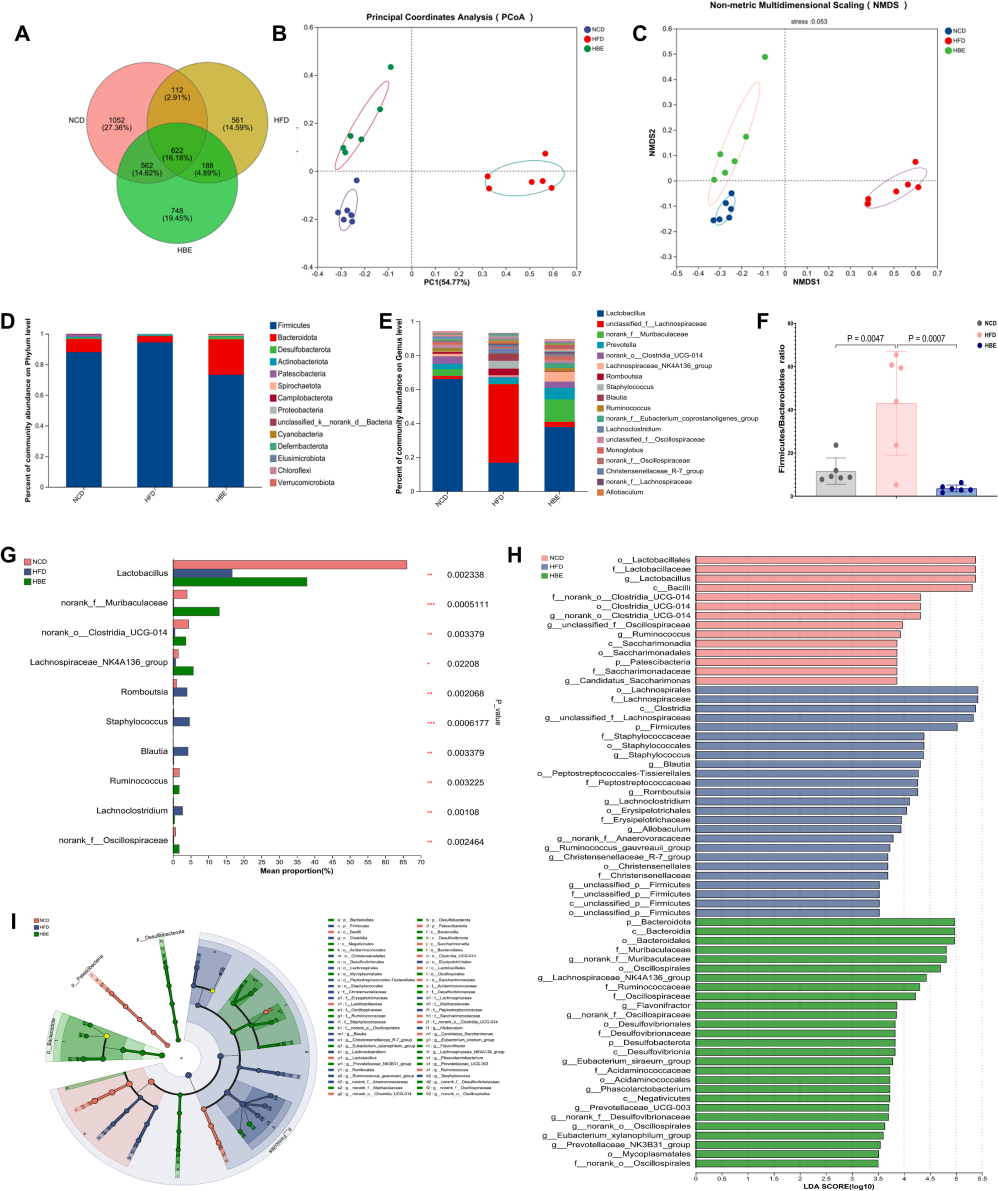
Betaine regulated gut microbiota. **(A)** Venn diagram **(B)** PCoA and **(C)** NMDS of gut microbiota; **(D)** the relative abundance of gut microbiota at the phylum level; **(E)** the relative abundance of gut microbiota at the genus level; **(F)** Firmicutes/Bacteroidetes ratio;**(G)** Kruskal-Wallis H test of the relative abundance of top 10 genera at the genus level; **(H)** the taxonomic cladogram and **(I)** histogram of LDA score based on LEfSe analysis (LDA > 3.5 was considered to be the differential characteristic taxon).

Initial investigations have demonstrated the interrelationship between gut microbes and obesity, as well as the contribution of betaine as an edible extract in daily foods to microbiome homeostasis in obese individuals. However, its fundamental regulation mechanism is still mostly unknown. To assess changes in microbial community diversity between the three groups, we measured the ace index (*p* = 0.041) and the chao index (*p* = 0.078), which showed a significant reduction in *α* diversity in the HFD group (Supplementary Figures 1A,B). These findings were consistent with the petal plot analysis, which indicated that betaine supplementation led to an increase in amplicon sequence variants (ASVs) (Figure 2A). Principal coordinates analysis (PCoA), non-metric multidimensional scaling (NMDS) and community heatmap analysis shown that HFD altered the gut microbiota profile significantly but betaine remodelled its profile to that of NCD (Figures 2B,C; Supplementary Figure 1C).

Additionally, an investigation was conducted into the effects of betaine on gut microbiota composition. As depicted in Figure 2D, at the phylum level, Firmicutes predominated with approximately 90%, trailed by Bacteroidetes, Desulfobacterota, and Actinobacteriota. A 16-week HFD led to a notably higher Firmicutes/Bacteroidetes (F/B) ratio in the HFD group compared to the normal control diet (NCD) group. After 8-week betaine treatment, relative abundance of Bacteroidetes was significantly increased while that of Firmicutes was decreased, resulting in a decreased F/B ratio (Figure 2F). At the genus level, the relative abundance of *Lactobacillus* significantly decreased, while that of *Lachnospiraceae* increased markedly under the HFD. Betaine administration mitigated this shift, leading to a gut microbiota profile similar to that of the NCD group (Figure 2E). These findings were also in agreement with the results from PCoA and NMDS analyses. The Kruskal-Wallis H test of the relative abundance of top 10 genera at the genus level indicated that the relative abundance of *Muribaculaceae*, *Clostridia*_UCG-014, *Lachnospiraceae*_NK4A136_group, and *Ruminococcus* was also lowered by HFD, but elevated by betaine. Meanwhile, HFD raised the relative abundance of *Blautia* and *Romboutsia* (Figure 2G). Furthermore, LEfSe analysis was conducted to investigate the particular microorganisms affected by betaine, with the results displayed in Figures 2H,I. At the genus level, *Lactobacillus*, *Clostridia*_UCG-014, *Ruminococcus* were the differentially characterized genera for the NCD group, while Staphylococcus and *Lachnospiraceae*_NK4A136_group were, respectively, for the HFD and HBE groups. In addition, the relative abundance of *Oscillospirales* and *Ruminococcaceae* were also significant in the HBE group.

### 3.3 Betaine alleviates obesity-related metabolic disorders through enhancing the production of SCFAs via the gut microbiota

**Figure 3:**
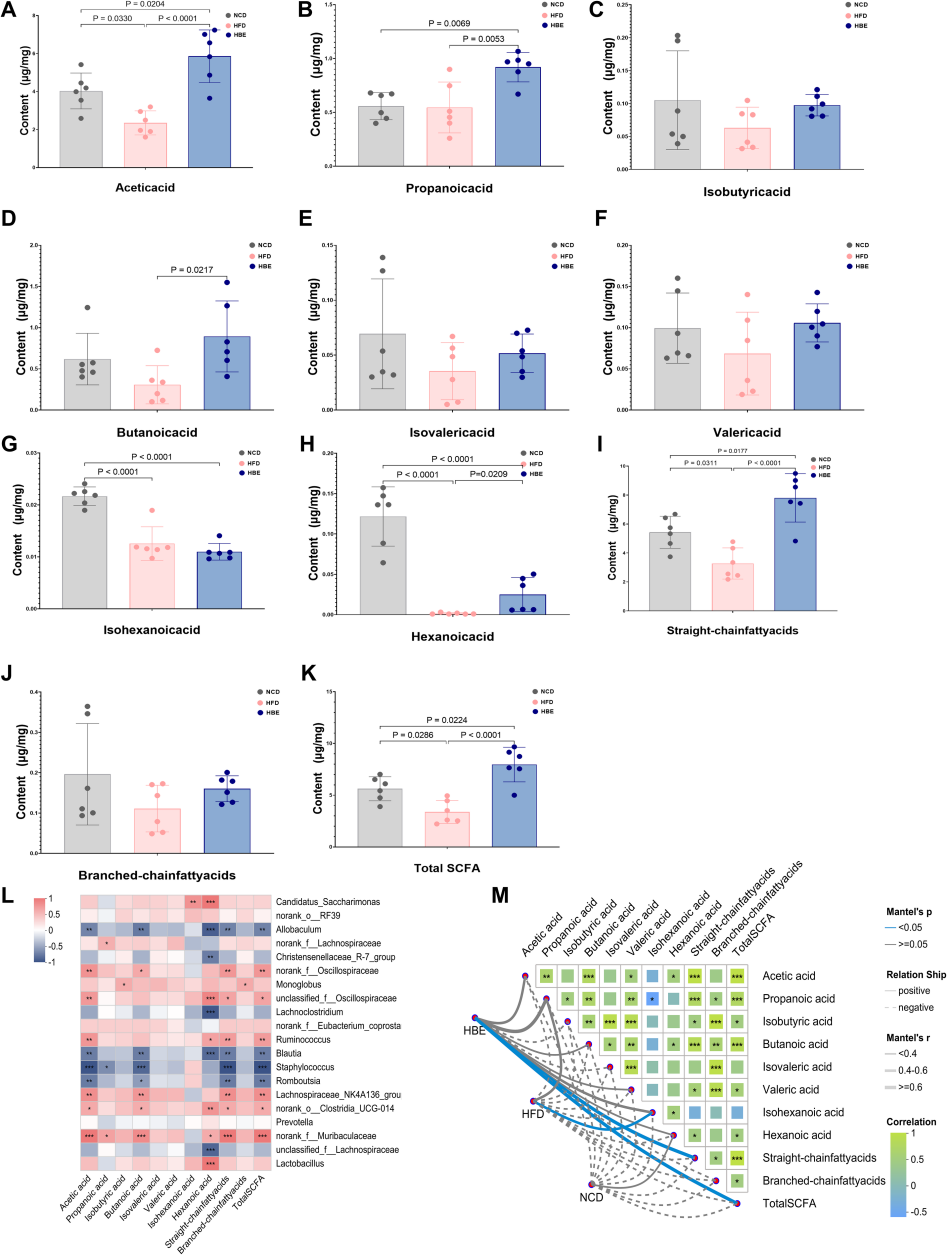
Betaine alters the SCFAs content in feces. **(A)** Acetic acid, **(B)** propanoic acid, **(C)** isobutyric acid, **(D)** butanoic acid, **(E)** isovaleric acid, **(F)** valeric acid, **(G)** isohexanoic acid, **(H)** hexanoic acid, **(I)** straight-chain fatty acids, **(J)** branched-chain fatty acids, and **(K)** total SCFA cotent in feces; **(L)** the Spearman’s correlation heatmap of gut microbiota (top 20) and SCFAs cotent; **(M)** Mantel-test analsis of the correlations between the gut microbiota composition (genus level) and SCFAs cotent in HBE, HFD and NCD groups. Data were expressed as mean ± SD (*n* = 6).

We analyzed SCFA composition in fecal samples from NCD, HFD and HBE groups to observe the response of SCFA to betaine treatment. Compared with the NCD group, HFD reduced the concentration of acetic acid,butanoic acid, valeric acid, hexanoic acid, isobutyric acid, isovaleric acid, isohexanoic acid, as well as the straight-chain fatty acids, branched-chain fatty acids, and total SCFAs. However, almost all of the above SCFAs reductions were increased after betaine intervention (Figures 3A–K). To obtain deeper understanding of the effects of SCFAs on obesity and metabolic processes, we conducted a correlation analysis of differential genus (top 20) and SCFAs concentrations. The results indicated that *Blautia* and *Romboutsia* were associated with a reduction in multiple SCFAs, whereas the representative genus of HBE group (*Lachnospiraceae*_NK4A136_group, *Ruminococcus*, *Muribaculaceae*, *Clostridia*_UCG-014) were linked to an increase in several specific SCFAs and total SCFAs. Furthermore, there was a positive correlation between the concentration of *Lactobacillus* and hexanoic acid (Figure 3L). As shown in Figure 3M, Mantel-test analysis also demeonstrated that straight-chain fatty acids (Mantel’s r = 0.76, *p* = 0.028) and total SCFAs (Mantel’s r = 0.76, *p* = 0.027) are the best explanator variable for HBE group and isohexanoic acid is highly correlated to HFD group (Mantel’s r = 0.44, *p* = 0.026). A more detailed description of the variables is available in Supplementary Table 1.

### 3.4 Administration of betaine modifies the lipidomic profiles within the adipose tissues of rats

**Figure 4:**
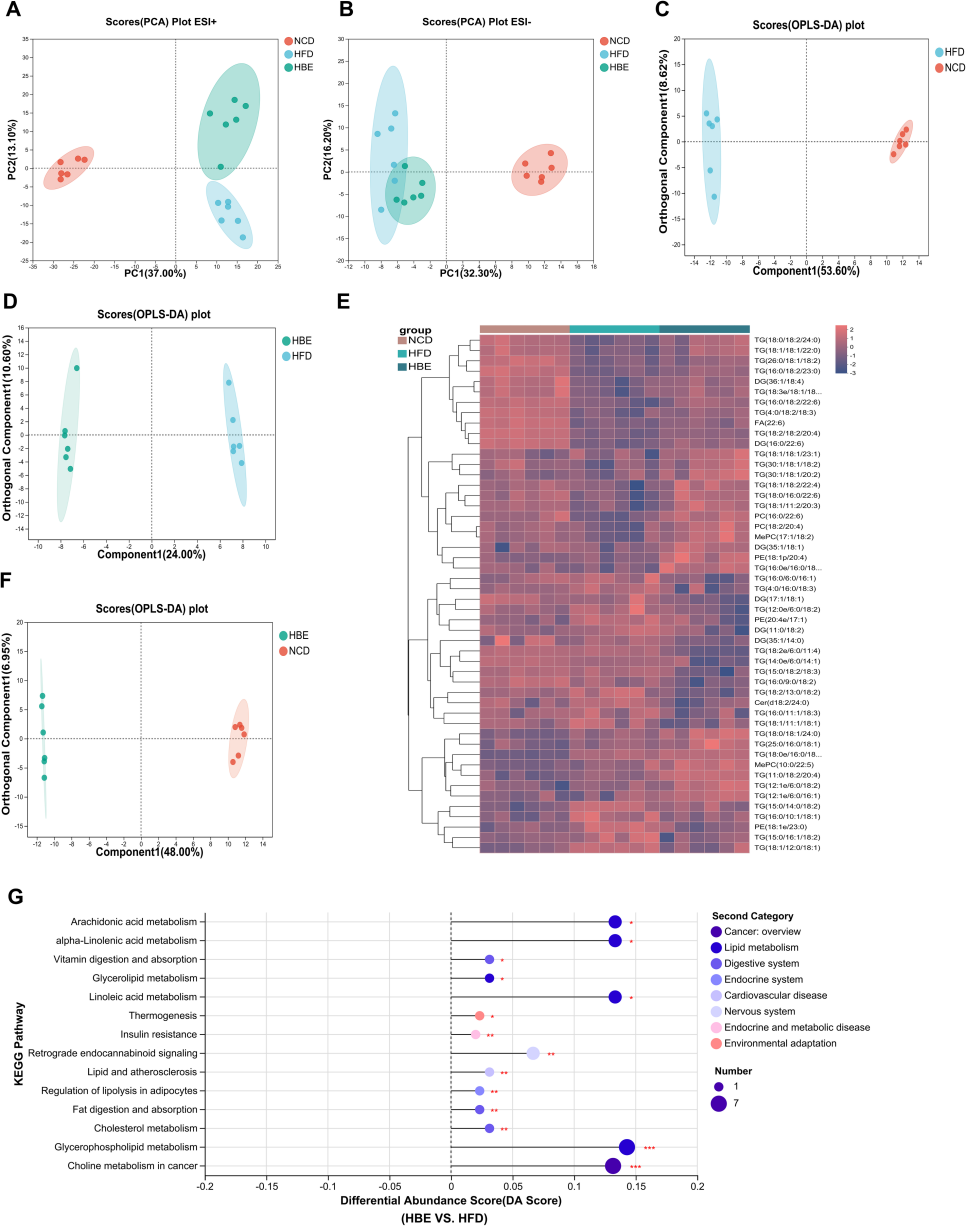
Betaine was associated with lipid metabolism in HFD-fed rats. Lipidomic analysis was performed on adipose tissue samples of rats (n = 6). **(A)** PCA plot in positive ion; **(B)** PCA plot in negative ion; **(C)** OPLS-DA plot of NCD vs. HFD; **(D)** OPLS-DA plot of HFD vs. HBE; **(E)** Top 50 different classes of lipids compared by heatmap; **(F)** OPLS-DA plot of NCD vs. HBE; **(G)** Differential abundance score of significant KEGG pathways (HFD vs. HBE).

To further verify the effects of betaine on lipid metabolism in the adipose tissues, a lipidomic analysis was performed using LC–MS/MS. A total of 1,511 metabolites were annotated, with 1,283 ESI + ions and 228 ESI- ions detected, respectively. The characteristic relative standard deviation (RSD) analysis of quality control samples showed that the repeatability and stability of lipidomics analysis were qualified (Supplementary Figures 3A,B). Principal component analysis (PCA) showed that significant differences were observed between HFD-fed and NCD groups (Figures 4A,B), while betaine treatment resulted in characteristics in the HBE group more similar to those in the NCD group. Orthogonal partial least squares discriminant analysis (OPLS-DA) plot exhibited a distinct segregation pattern among the three groups in terms of adipose tissues (Figures 4C,D,F). In hierarchical cluster analysis of differentially altered lipids (DALs), significant changes in lipid profiles were found in rats fed HFD compared with the NCD group, and betaine treatment further modulated HFD-induced dysregulation of lipid metabolism (Figure 4E). Figure 4G; Supplementary Figures 3C,D displayed the enrichment of pathways via KEGG analysis, with regulation of lipolysis in adipocytes, glycerophospholipid (GP) metabolism, alpha-linolenic acid metabolism and thermogenesis pathway were up-regulated (differential abundance score >0.1, *p* < 0.001) in HBE group than in HFD group. Besides, the DALs observed between the HBE and HFD control groups were predominantly enriched in various lipid metabolic pathways, including choline metabolism in cancer, cholesterol metabolism, fat digestion and absorption, lipid and atherosclerosis, retrograde endocannabinoid signaling, insulin resistance, vitamin digestion and absorption, and arachidonic acid metabolism pathway.

### 3.5 Administration of betaine alters the components of lipids in adipose tissues

**Figure 5:**
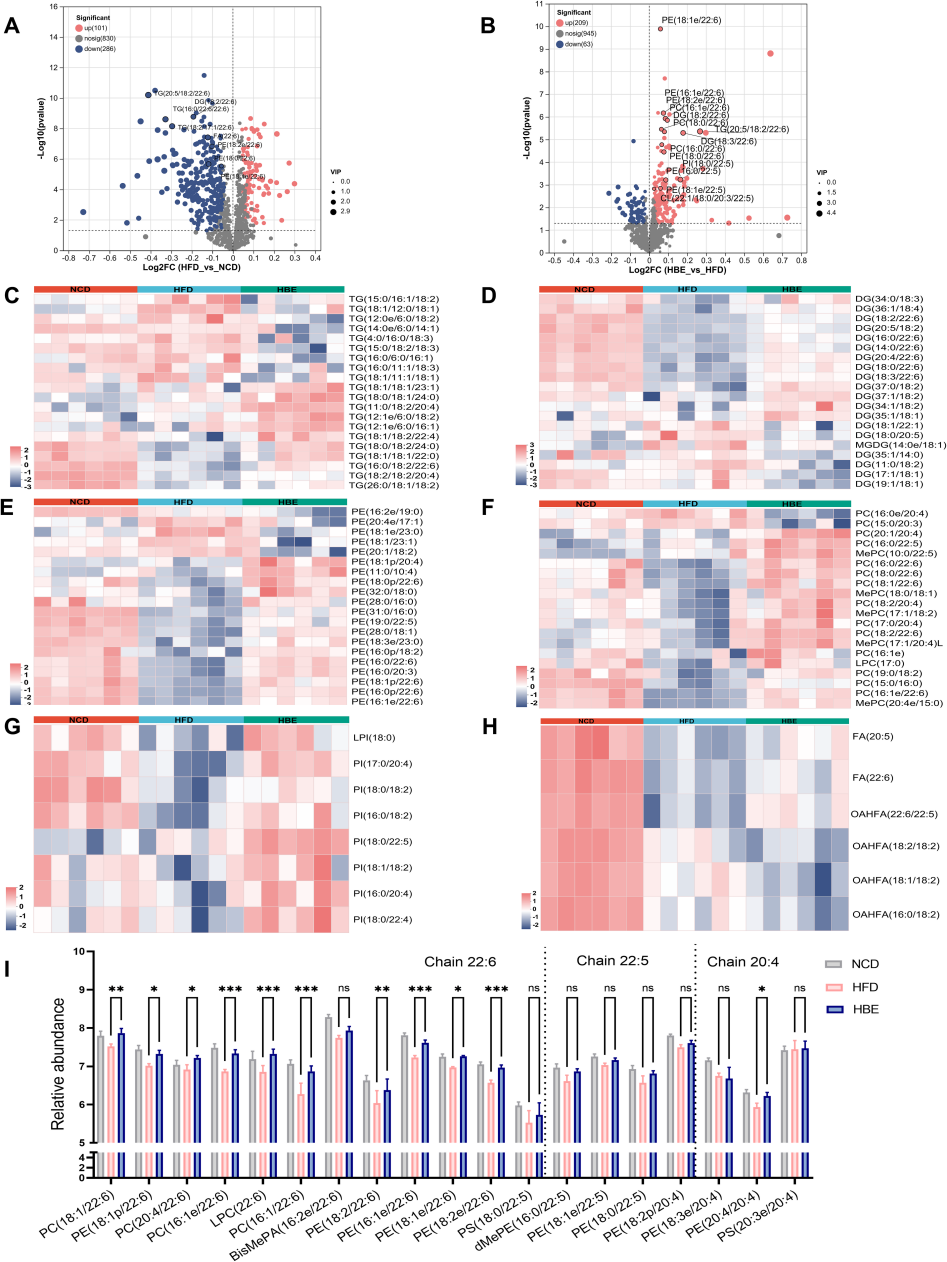
Betaine alters lipid metabolism of adipose tissues. Volcano plot showing differentially lipid in the comparison of **(A)** HFD group vs. NCD group and **(B)** HBE group vs. HFD group (*p* < 0.05; fold change > 1). Heatmap analyses show top 20 **(C)** triglycerides (TG), **(D)** diglyceride (DG), **(E)** phosphatidylcholine (PC), **(F)** phosphatidylethanolamines (PE), **(G)** phosphatidylinositol (PI), and **(H)** fatty acids (FA) species in HFD group, NCD group and HBE group. **(I)**The relative abundance of individual glycerophospholipids species with chain 22:6, 22:5, and 20:4 in eWAT from 3 groups. Data were given as the mean ± SD, *n* = 6.*p < 0.05, **p < 0.01,***p < 0.001 determined by one-way ANOVA analysis.

To further determine the preferences of betaine on regulating lipid metabolism in adipose tissues, we explored the changes in content and composition of different lipid classes. Glycerolipids (GLs), comprising triglycerides (TG), diglycerides (DG), and free fatty acids (FFA), constitute the primary constituents of adipose tissue and serve as significant substrates for lipid oxidation. The total abundance of TG and DG did not change significantly between HFD and HBE (Supplementary Figures 2A,B), however, further analysis of the composition of glycerolipids showed that the species of DG and TG had changed. After betaine treatment, the most downregulated TG species were chain lengths of 50 carbons or less (C < 50) with 2–3 double bonds, whereas chain lengths of 55 carbons or more (C > 55) with more than 4 double bonds were the most upregulated, including TG(18:1/18:2/22:4), TG(16:0/18:2/22:6), TG(18:2/18:2/20:4), TG(11:0/18:2/20:4) (Figure 5C). When there is an energy demand, the TGs containing very long chain fatty acids in lipid droplets can be mobilized and rapidly transported to the peroxisome for fatty acid oxidation. However, these TG species were significantly lower in HFD group than in NCD group. Notably, species analyses of DG as a precursor to TG showed similar differences between the three groups. Compared with the NCD group, DG species with more than 4 double bonds were downregulated in HFD group and relieved after the betaine intervention. Interestingly, of the 11 detected DGs that produced the above changes, 6 were DG species that contained docosahexaenoic acid (DHA, 22:6), including DG (18:2/22:6), DG (16:0/22:6), DG (20:4/22:6), DG (18:0/22:6), and DG (18:3/22:6) (Figure 5D). It is generally accepted that DHA modulates energy metabolism and exhibits beneficial properties in anti-inflammatory processes, as well as enhancing insulin secretion. Consistent with DG, all 3 fatty acids (FA) species that contained more than 4 double bonds were increased, while 2 FA species that contained of 2–3 double bonds were decreased (Figure 5H).

Although glycerophospholipids (GPs) represent a small fraction of the lipidome, they are not only the main components of the cell membrane, but also participate in regulating the dynamics and homeostasis of the cell membrane. We observed that multiple phospholipid species, such as phosphatidylethanolamine (PE), phosphatidylcholine (PC), and phosphatidylinositol (PI), decreased after 12-week of HFD feeding and relieved after 8-week of betaine intervention. (Supplementary Figures 3D–F). GPs are the main components of mitochondrial membranes, and an increase in their content in adipose tissue suggests an enhancement in mitochondrial function and energy metabolism levels of adipocytes. Interestingly, in top 20 differential species of the GP subclasses, PE, PC and PI species with 20:4/22:5/22:6 chain also increased in the NCD group and HBE group, whereas in the HFD group we observed a decline pattern (Figures 5E–G). We presented the concentration levels of specific GP species featuring a chain of 22:6, 22:5, and 20:4 in Figure 5I. Most of GP with chain 22:6, 22:5, and 20:4 was higher in NCD group and HBE group, especially chain 22:6. Additionally, volcano plots also demonstrated that most of the lipid species with chain 22:6 and 22:5 were increased in response to betaine (Figures 5A,B). Our study suggests that betaine have a beneficial effect on adipose tissue by regulating lipid composition and promoting lipolysis.

### 3.6 Betaine treatment regulated the expression of genes involved in lipid metabolism

**Figure 6:**
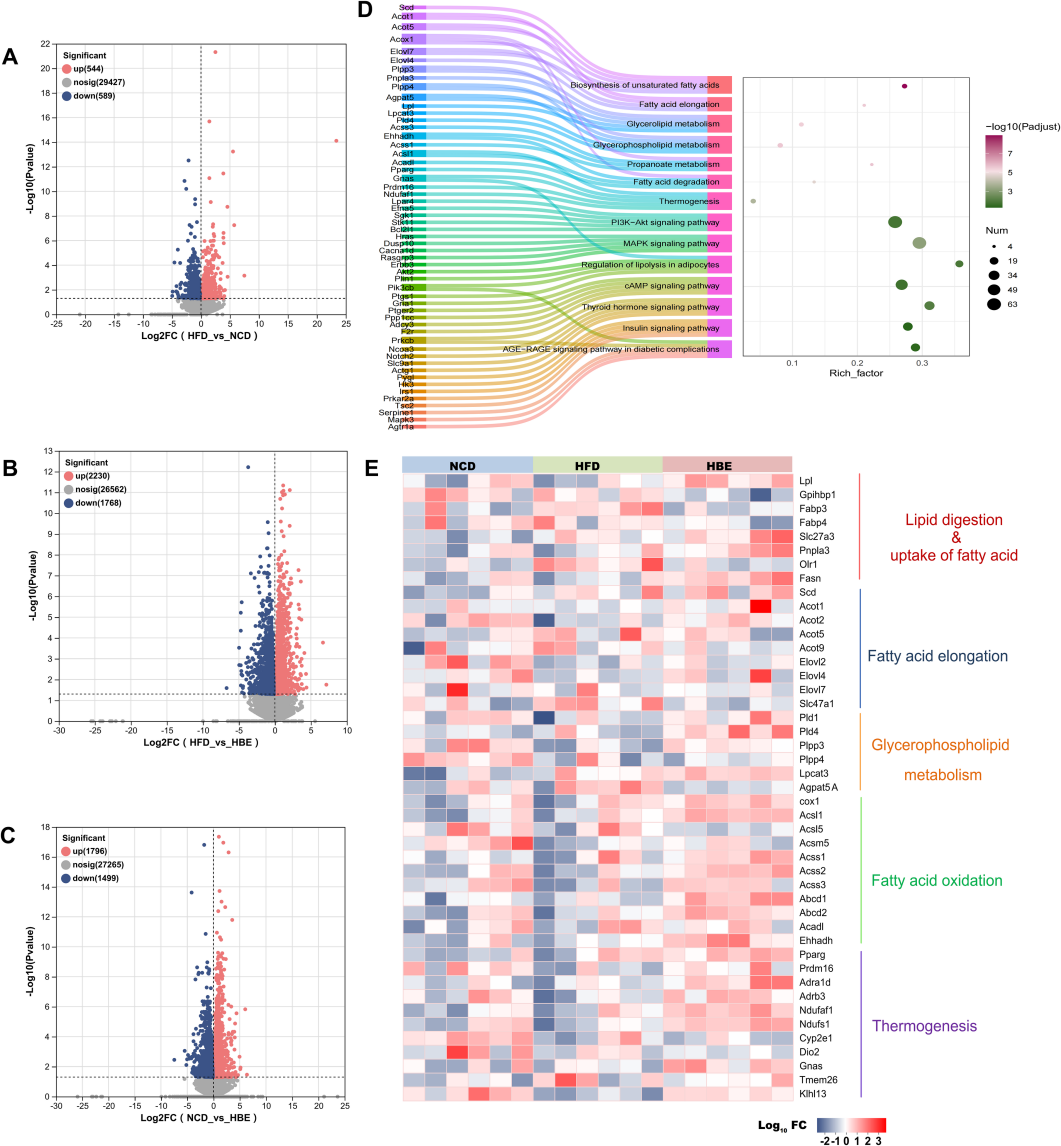
Betaine regulated adipose tissue transcriptome in a large scale. RNA-seq analysis was performed on adipose tissue samples of rats (*n* = 6). **(A-C)** Volcano plot showing differentially expressed genes in the comparison of HFD group vs. NCD group, HBE group vs. HFD group and NCD group vs. HBE group. (*p* < 0.05; fold change > 1) **(D)** KEGG pathway enrichment analysis based on DEGs in HFD group vs. HBE group. **(E)** Relative expression levels of selected genes from the RNA-seq dataset for important enzymes or regulators that involve in lipid digestion, uptake of fatty acid, glycerophospholipid metabolism, fatty acid elongation, fatty acid oxidation and thermogenesis.

To further explore the underlying mechanisms of betaine in regulating lipid metabolism, RNA-seq-based transcriptomic analysis was conducted to identify potential molecular targets. Supplementary Figures 4A–C showed that the sequencing saturation was close to 1, indicating high-quality transcriptomic data. Analysis of differential expression (Figures 6A–C) revealed 1,133 DEGs between the HFD control and NCD groups, with 544 genes being upregulated and 589 genes downregulated. PCA analysis showed that betaine separated HBE group from HFD group, which accounted for 29.86 and 11.41% of total variation, respectively (Supplementary Figure 4D). The RNA-seq data further highlighted that betaine markedly altered the transcriptomic landscape in the adipose tissues of HFD-fed rats compared to betaine-treated rats. A total of 3,998 DEGs were identified in HBE group vs. HFD group (Figure 6B). These DEGs were predominantly enriched in pathways linked to lipid metabolism, including fatty acid degradation, glycerophospholipid metabolism, biosynthesis of unsaturated fatty acids, and MAPK signaling pathway, aligning with our histopathological and lipidomic analyses (Figure 6D). Furthermore, KEGG and GO pathway analysis indicated that DEGs in betaine-treated rats versus HFD-fed rats were also enriched in processes related to carbohydrate metabolism and thermogenesis, such as thyroid hormone, insulin, and AGE-RAGE signaling pathway in diabetic complications (Figure 6D; Supplementary Figure 4E). Collectively, these results implied that betaine modulated intra-adipose lipid profiles through extensive regulation of genes involved in energy metabolism.

Subsequently, our analysis concentrated on the notable alterations in gene expression, particularly those regulating important enzymes involved in lipid transport, glycerophospholipid metabolism, fatty acid elongation processes, fatty acid oxidation, and adipose tissue thermogenesis during HFD and betaine administration intervention. The major genes involved in lipid digestion and fatty acid transportation increased in the betaine-treated adipose tissues, including *Lpl*, a gene encoding a crucial hydrolytic enzyme that catalyzes the hydrolysis of triglycerides in lipoproteins into fatty acids and monoglycerides; *Slc27a3*, a gene encoding a protein with acetyl-coenzyme A (acyl-CoA) synthetase activity that catalyzes the first step of fatty acid beta oxidation by linking fatty acids to CoA to form fatty acyl-CoA esters. In addition, genes that were crucial for lipolysis and oxidation were upregulated, including *Pnpla3*, a gene encoding a key enzyme involved in lipolysis which catalyzes the release of fatty acids from stored triglycerides (TG) within adipocytes; Acsl1, a gene encoding a rate-limiting enzyme in lipid metabolism, specifically involved in the activation of long-chain fatty acids (LCFAs) for their subsequent utilization in cellular lipid synthesis or degradation via beta-oxidation. Moreover, betaine treatment also significantly increased the expression of phospholipid metabolism genes such as *Pld1*, *Pld4*, *Plpp3*, *Plpp4*, *Lpcat3*, thermogenic genes such as *Pparg*, *Prdm16*, *Ndufaf1*, and beige marker genes such as *Dio2*, *Tmem26*, *Klhl13* and *Sla27a3* (Figure 6E).

### 3.7 Integrated analysis of transcriptome and lipidomic in lipid metabolism

**Figure 7:**
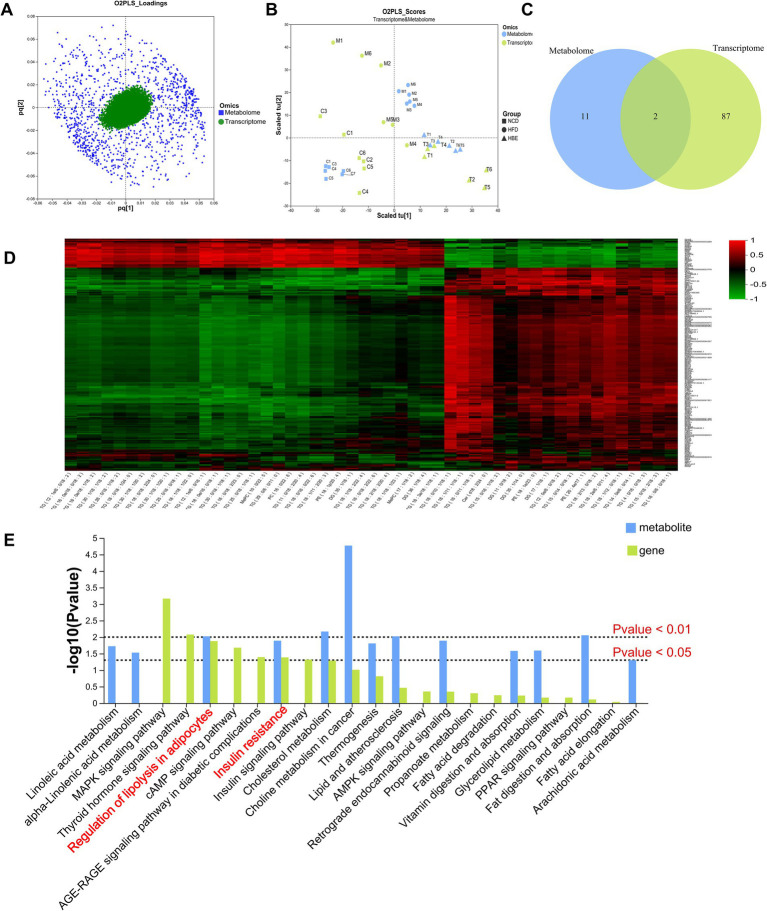
Correlation analysis. **(A)** The O2PLS model of loadings plot. **(B)** The O2PLS model of joint score plot. **(C)** Venn diagram of the number of pathways involved in differential genes and differential metabolites. **(D)** Hierarchical cluster heat map for Spearman correlation analysis of differential genes and differential metabolites. Correlation coefficient r is indicated by color. r > 0 indicates positive correlation, represented by red, r < 0 indicates negative correlation, represented by green, and lighter color indicates stronger correlation. **(E)** The *p* value histogram of KEGG enrichment analysis for integrated metabolomics and transcriptomics. The x-axis indicates the enriched metabolic pathways, the y-axis represents –log (*p*-value), green represents the enriched p value of DEGs, and blue represents the enriched *p* value of the diferential metabolites.

**Figure 8:**
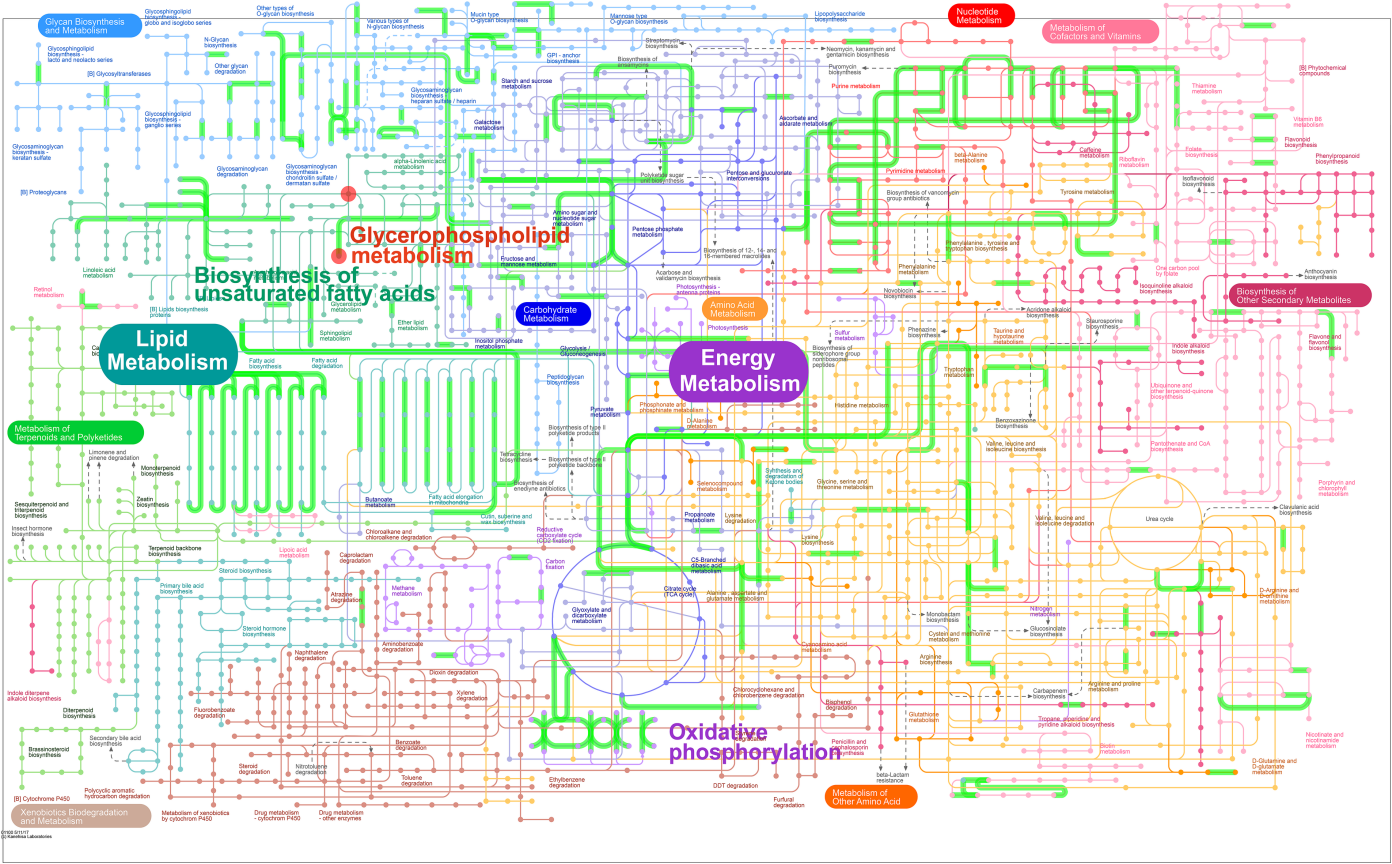
Integrated transcriptome and lipidomic analysis in iPath 3.0 for visualizing KEGG general metabolic pathway map. Red dots represent differential metabolites (HFD group vs. HBE group) and green lines represent DEGs (HFD group vs. HBE group), respectively.

In order to explore the combined role of genes and metabolites in the defense of betaine against HFD, we used spearman correlation and O2PLS to integrate transcriptomes and lipidomic. The findings indicated that the overall expression trend of the transcriptome and metabolic group samples was the same (Figures 7A,B) and spearman correlation hierarchical clustering analysis can cluster genes and metabolites with similar expression patterns. All these indicate a well correlation between transcriptomics and metabolomics (Figure 7D). The jointly analysis of KEGG pathway showed that DEGs and differentially expressed metabolites were mainly involved in two pathways, including regulation of lipolysis in adipocytes and insulin resistance (Figure 7C; Supplementary Table 2). At the same time, three co-enriched pathways, thermogenesis, retrograde endocannabinoid signaling, and choline metabolism in cancer were significantly changed (Figure 7E). To further verify this observation, a comprehensive analysis was conducted by integrating significant differential metabolites (VIP > 1, *p* < 0.05) with DEGs (FC ≥ 1.0, *p* < 0.05) within iPath 3.0, aiming to further elucidate the metabolic pathways altered by betaine intervention (Figure 8). When comparing HFD to HBE, the primary enriched pathways were lipid metabolism and energy metabolism. Based on the combined transcriptome and metabolome analysis, it is hypothesized that betaine may mitigate obesity resulting from HFD through its modulation of energy production and lipid metabolic pathways.

### 3.8 Effect of betaine on serum gastrointestinal hormone levels

**Figure 9:**
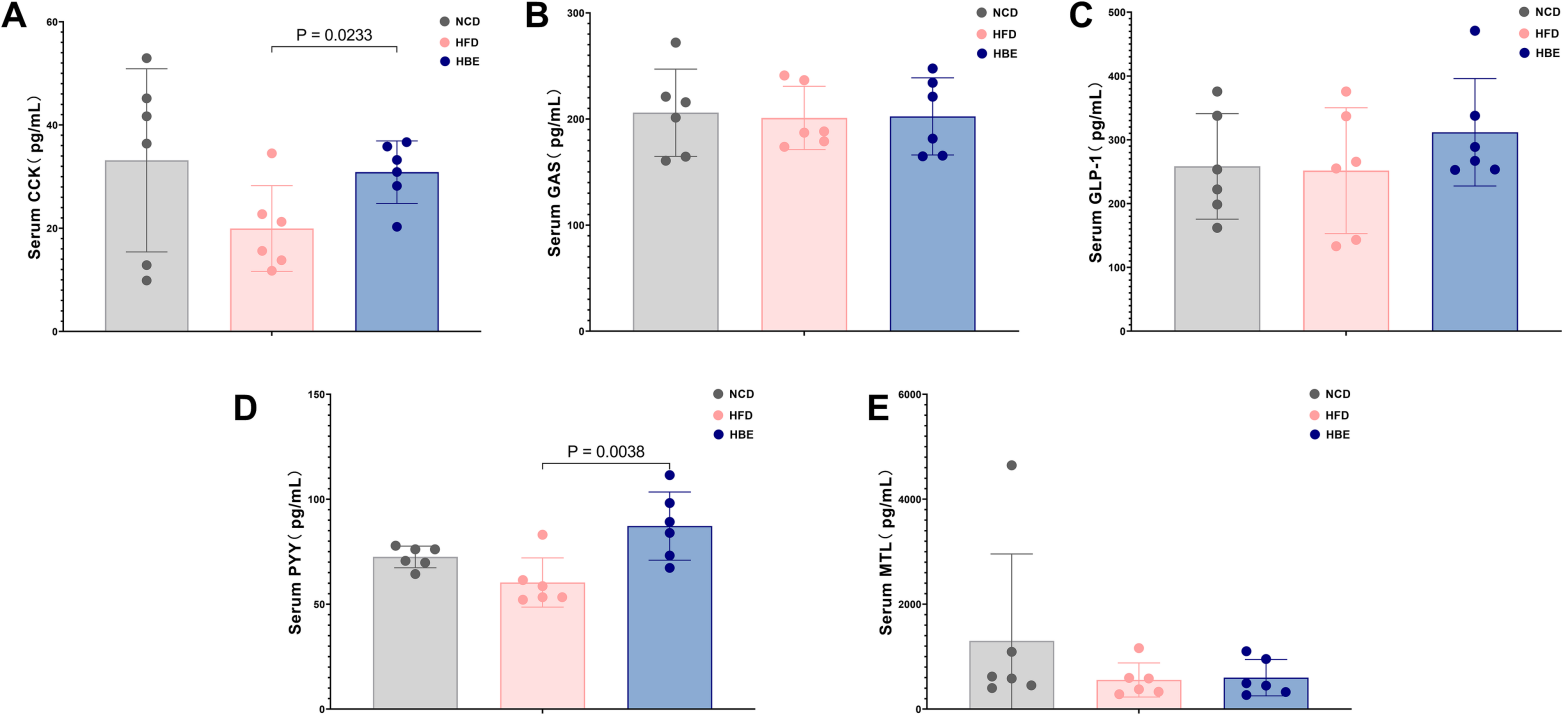
Effect of betaine on serum gastrointestinal hormone levels. **(A)** Cholecystokinin (CCK), **(B)** gastrin (GAS), **(C)** glucagon-like peptide-1(GLP-1), **(D)** peptide YY(PYY) and **(E)** motilin (MTL) levels in serum. Data were expressed as mean ± SD (*n* = 6).

To explore the effects of betaine on the levels of gastrointestinal hormones, the levels of serum glucagon-like peptide-1(GLP-1), cholecystokinin (CCK), motilin (MTL), gastrin (GAS) and peptide YY (PYY) were compared between the three groups. Compared with the NCD group, the HFD reduced the serum CCK, PYY and MTL levels, while the 8-week betaine treatment increased the serum GLP-1, CCK and PYY levels in the HBE group, and the differences in CCK and PYY levels between the HBE group and the HFD group were statistically significant as shown in Figure 9.

### 3.9 Analysis of correlations among obesity-associated biochemical indicators, gut microbiota, SCFA, gastrointestinal hormones, differential metabolites, and differential genes in adipose tissues

**Figure 10:**
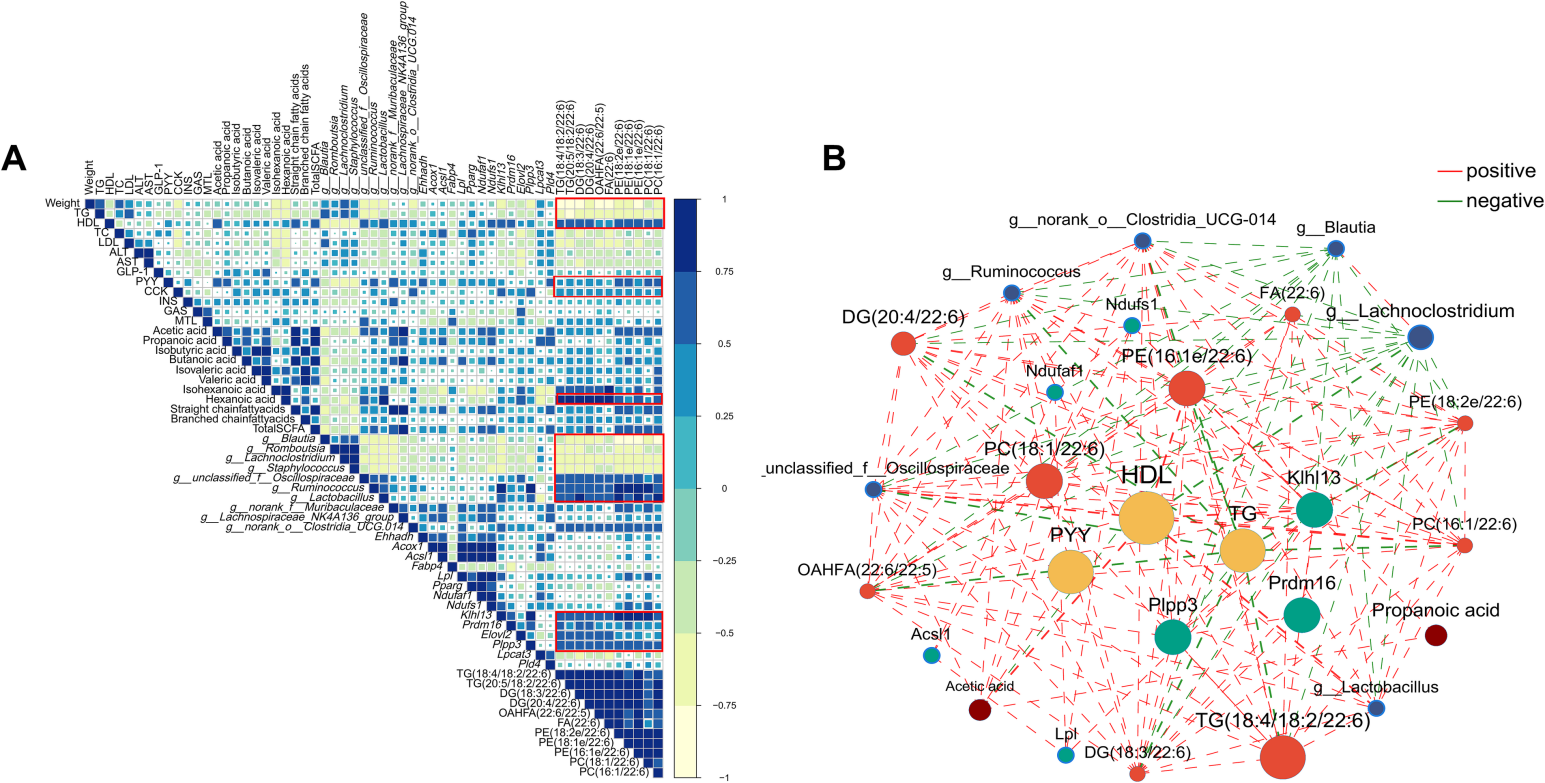
The Spearman’s correlation. **(A)** heatmap and **(B)** network (only correlations with |R| > 0.5 with *p* < 0.01 were presented) among lipid-related biochemical indexes, gut microbiota, short-chain fatty acid, differential genes and differential metabolites. Yellow nodes represented lipid-related biochemical indexes, blue nodes represented gut microbiota, green nodes represented differential genes, red nodes represented differential metabolites and brown nodes represented short-chain fatty acid. The red lines represented positive correlations while the green lines represented negative correlations.

Spearman’s correlation analysis was employed to examine the relationships between obesity-related biochemical indexes, microbial genera, SCFA, gastrointestinal hormones, differential metabolites, and genes (Figure 10A; Supplementary Tables 3, 4). Moreover, the correlation network highlighted significant and strong associations among these six components (|R| > 0.5, *p* < 0.01, as shown in Figure 10B. Overall, several lipid species with chain 22:6, including TG(18:4/18:2/22:6), TG(20:5/18:2/22:6), DG(18:3/22:6), DG(20:4/22:6), OAHFA(22:6/22:5), FA(22:6), PE(18:2e/22:6), PE(18:1e/22:6), PE(16:1e/22:6), PC(18:1/22:6), and PC(16:1/22:6), displayed significantly positive correlations with HDL, PYY, CKK, *Lactobacillus*, *Ruminococcus*, *Oscillospira*, hexanoic acid, genes related to thermogenesis, and significantly negative correlation with body weight and TG. In short, correlation analysis results revealed that these genera, SCFA, hormones, genes, and lipid species with chain 22:6 could serve as potential biomarkers for obesity alleviation.

## 4 Discussion

Obesity, a prevalent global health concern, is commonly marked by the accumulation of fat, dyslipidemia, systemic low-grade inflammation and disturbances in the gut microbiota. Betaine is a kind of natural material, widely exists in the daily food and its deficiency is associated with many obesity-related chronic metabolic diseases. Previous studies have demonstrated that betaine plays a crucial role in the regulation of lipid metabolism by enhancing fatty acid oxidation, modulating lipoprotein esterase activity and acting as a methyl donor in metabolic processes (23, 29, 30). However, most of these studies are based on the liver’s role in energy regulation and emphasize the role of betaine in liver lipid-related pathology. The extent to which betaine can effectively alleviate metabolic disorders by regulating adipose tissue lipid metabolism is still under debate (31, 32). To investigate the effects of betaine on adipose tissue metabolism and its underlying systemic mechanisms, we employed a multi-omics approach to examine its influence on genes associated with lipid metabolism, fatty acid profiles, and the composition of the gut microbiota in the context of a high-fat diet. Herein, works in this regard showed that gut microbiota was important regulator of the function of betaine in reducing HFD-induced fat accumulation in the adipose tissues and improves dyslipidemia and hormone profiles. Furthermore, treatment with betaine results in significant alterations in the lipid composition and the expression patterns of genes associated with lipid metabolism. Our findings, obtained through multi-omics approaches, demonstrate that betaine exerts multifaceted effects on energy metabolism by enhancing fatty acid-oxidation, improving mitochondrial functionality, and elevating thermogenesis in adipocytes by improving the synthesis of polyunsaturated phospholipid rich in docosahexaenoic acid (DHA).

The accumulation of excessive lipid due to obesity causes hypertrophy of adipocytes, and the ectopic deposition of lipids in tissues and organs other than adipose tissue results in disorders of lipid and blood glucose metabolism. In our study, HFD increased the Lee’s index in rats, and the results of the 8-week dietary intervention indicated that betaine significantly alleviated this change. Similarly, the groups presented similar tendencies in body weight, fat mass, and lipid indexes. The results of lipid profile showed that betaine supplementation decrease TG level but increase the HDL-C level in the serum of HBE group compared with HFD group. This is in line with previous studies where betaine reduced TG in rats fed with HFD (33). Additionally, the GTT results of this study demonstrated that betaine can also regulate the stability of glucose. The assessment of hepatic damage typically involves measuring elevated levels of serum ALT and AST. Here, HFD increase ALT and AST while betaine ameliorated liver damage as well as HFD-induced transaminase elevation. These results suggest that betaine can enhance liver metabolism and alleviate obesity-related disorders such as glycaemia homeostasis and lipid disorders by reducing fat weight.

Research on animals and humans has previously revealed considerable differences in the gut microbiota of obese versus normal-weight individuals. Notably, deficiencies or disruptions in the gut microbiota are frequently implicated in the progression of obesity (34, 35). Our current findings show that betaine supplementation significantly alters the composition of the gut microbiota in HFD-fed rats, particularly the functional role of some bacteria in intestinal tract that intrigued us. *Lactobacillus* (36, 37), *Lachnospiraceae*_NK4A136_group (38), *Ruminococcus* (39), *Roseburia* (40), and *Oscillospira* (41) have been recognized as significant factors in preventing obesity, as they modulate lipid metabolism, alter the production of gastrointestinal peptides, maintaining gut health and immune defense. *Blautia*, belonging to the phylum Firmicutes, can stimulate insulin secretion and contribute to metabolic disorders, including hypertriglyceridemia, fatty liver, and insulin resistance, which is positively correlated with waistline, body mass index, low density lipoprotein, TG and total cholesterol (42, 43). Similarly, *Romboutsia* has also been identified as a pathobiont positively and significantly associated with body weight, serum lipids and uric acid (43). In line with our results, we observed an elevated abundance of *Lactobacillu*, *Lachnospiraceae*_NK4A136_group, *Ruminococcus*, *Roseburia*, and *Oscillospira* in HFD-fed rats after betaine supplementation, which were negatively correlated with body weight and TG. We also observed that the relative abundance of *Blautia*, which was positively correlated with body weight and TG, was significantly reduced in the HBE group.

SCFAs, a class of organic compounds with carbon chains less than 6 carbon atoms in length, are key metabolites produced by the gut microbiota. According to previous research, SCFAs have positive impacts on host metabolism by prevent excessive inflammation, regulate intestinal hormone secretion and reverses HFD-induced metabolic disorder via modulate the MAPK signaling pathway (44, 45). Conversely, deficiency of SCFAs is linked to the progression of T2DM, elevated adiposity, and obesity (46). In this study, transcriptomic data show that MAPK signaling pathways is among the top enriched pathways in adipose tissues in HBE-fed rats, and more SCFA-producing genera in the feces of NCD group or HBE group than in HFD group. In particular, betaine supplementation revised almost all SCFA species altered by HFD feeding, implying a potential connection between betaine’s obesity-protective effects and microbial-derived SCFAs. In addition, following betaine supplementation, acetate and butyrate increased significantly, and levels of isobutyric acid, isovaleric acid, valeric acid, hexanoic acid, and total short-chain fatty acids also elevated. Acetate and butyrate are the two most abundant microbes in the feces, and previous studies have highlighted their importance in the treatment of obesity and obesity related metabolic disorders (47). Similar results were found in our study, where we also demonstrated that hexanoic acid also contributed to inhibit HFD-induced metabolic disorders. Wu, F. et al. found that the intake of hexanoic acid was negatively correlated with the risk of overweight/obesity and increased the intake of hexanoic acid may have a protective effect against overweight/obesity (48).

Adipose tissues are crucial for human energy homeostasis. Targeting energy expenditure in adipose tissue is emerging as a promising strategy in the battle against obesity. In this study, we focused on the beneficial effects of betaine on adipose tissue in HFD-fed rats. The metabolism and mass of adipose tissue are primarily influenced by adipocyte size and turnover, and changes in these factors are linked to pathological conditions (49). In pathological analysis, the adipose tissue weight of the rats treated with betaine was significantly reduced, small adipocytes increased, and large adipocytes decreased. Adipocyte hypertrophy is closely related to dyslipidemia and insulin resistance in humans (50, 51). The decrease in adipocyte size observed in betaine-treated obese rats may suggest enhanced insulin sensitivity within their adipose tissues.

Lipids serving as the energy storage and inter/intra-cellular signaling molecules, which allows them to directly read out the metabolic state of the cell (52). However, a comprehensive lipidomic assessment of the effects of betaine on obesity that can define specific lipid alterations is still largely lacking. In this study, the primary lipid type we analyzed across three groups was TG, which comprised more than 95% of adipose tissues (Data not shown). Nonetheless, betaine extensively regulated the TG profiles, exhibiting both positively and negatively effects. In the demand of energy, triglycerides containing very long chain fatty acids in lipid droplets can be quickly mobilized to peroxisome for fatty acid oxidation (53). We observed that the most downregulated TG species after betaine treatment were chain lengths of 50 carbons or less (C < 50) with 2–3 double bonds, whereas chain lengths of 55 carbons or more (C > 55) with more than 4 double bonds were the most upregulated. The pattern of composition observed here aligned with findings from another study which reported that TG with chain lengths of 56 carbons with 2–3 double bonds were significantly lower than brown adipose tissues in isceral adipose tissue, which is contributes to the high risk of metabolic diseases (54). GPs are important lipids involved in cellular energy metabolism, and our lipidomic profiles have shown that phospholipid also displayed a distinct distribution in HFD group and HBE group. As well known, PC and PE are the basic substances to maintain the structure and function of the inner mitochondrial membrane, while PI is the key determinant of cytomembrane identity and act as spatiotemporal cues to direct membrane dynamics. The elevated PC, PE and PI after betaine treatment suggested an enhanced mitochondrial activity in adipose tissues. More specifically, PE and PC containing side chain of very-long chain (n ≥ 20) fatty acyl, such as C20:4, C22:4, C22:5, C22:6, especially those rich in DHA (C22:6), were significantly increased in HBE group.

Consistent with the findings of lipidomics, the transcriptomic analysis indicated that significant alterations in gene expression were also observed in fatty acid transport, lipolysis, thermogenesis and other lipid metabolism related pathways. Adipocytes synthesize and secrete LPL, an enzyme that hydrolyzes TGs in circulating capillaries to produce free FAs, which is then transported to the capillaries (55). After the release of free FAs by LPL, FATP3/SLC27A3 facilitates the uptake of FAs into adipocytes and is responsible for approximately 30% of long chain and ultra-long chain acyl-CoA synthetase activity (56). Once taken up by adipocytes, fatty acids are traf-ficked to the mitochondria for the purpose of undergoing oxidation, providing substrates for ACSL1. ACSL1 then activates these fatty acids to FA-CoA, making them available for *β*-oxidation, glycerophospholipid synthesis, and other metabolic pathways (57). In our study, we found that betaine could up-regulate the expression of *Lpl*, *Slc27a3*, *Acsl1* genes and promote the lipid digestion and fatty acid transportation. The *Elovl2* and *Elovl4* genes play pivotal roles in the synthesis of long-chain polyunsaturated fatty acids (LC-PUFAs) (58, 59). Figure 6E demonstrates a significant upregulation of genes associated with fatty acid synthesis, such as *Elovl2*, *Elovl4*, and *Fasn*, in the HBE group. This upregulation may be linked to the elevated levels of polyunsaturated fatty acids found in adipose tissue. LPCAT3 is an enzyme that specifically recognizes and catalyzes polyunsaturated acyl transfer reactions and plays an important role in producing phospholipids with a specific fatty acid composition (60). The proteins encoded by *Plpp3* and *Plpp4* gene have the activity of lysophosphatidic acid hydrolase, thereby affecting the synthesis and properties of phospholipids (61). Meanwhile, *Pld1* and *Pld4* as phospholipase D family genes play a pivotal role in the synthesis of specific phospholipid components. In our study, we found that betaine could up-regulate the expression of *Lpcat3*, *Plpp3*, *Plpp4*, *Pld1* and *Pld4* genes and promote the synthesis of LC-PUFAs, especially those polyunsaturated phospholipid rich in DHA.

In the past few years, studies focused on the metabolic process occurring in adipose tissues demonstrated that dietary supplementation with DHA could be used to prevent obesity related inflammation (62). Recently, numerous studies suggested that DHA in the form of phospholipids can effectively regulate glycolipid metabolism and alleviate obesity-related disorders via improve mitochondrial function and increased thermogenesis in adipocytes (63, 64). The upregulation of genes associated with fatty acid oxidation and thermogenesis primarily facilitated these reactions. In our present investigation, we further noted that the expression levels of thermogenic genes, including *Prdm16*, *Pparg*, *Ndufaf*, *Tmem26*, *Klhl13*, *Dio2*, and *Scla27a3*, were notably elevated in the HBE group. This upregulation correlated with the observations from the lipidomic analysis. These findings indicated that rats treated with betaine exhibited enhanced thermogenic and metabolic activities compared to those in the HFD group, resulting in increased thermogenesis and improved mitochondrial function. KEGG analysis also showed that several energy metabolism-related signaling pathways were the most enriched signaling pathways in adipose tissue, including MAPK signaling pathway, cAMP signaling pathway and PI3K-Akt signaling pathway. Based on the above results, we speculate that betaine can regulate fatty acid *β*-oxidation by activating lipid metabolism-related signals in adipose tissue, which is worthy of further exploration in future studies.

Lipids are the main components of adipocytes, so liposomal remodeling triggers specific signaling pathways to modulate energy metabolism. Some studies suggest that omega-3 fatty acids, including DHA, may indirectly impact on the secretion of various hormones of the gastrointestinal tract regulators (65, 66). Thus, we began to further explored whether betaine could affect the production of gut hormones and found that the secretion of GLP-1 was increased in HBE group than in HFD group. In addition, HFD decreased serum PYY and CCK levels but could be mitigated by betaine intervention. Moreover, some lipid species with chain C22:6 increased by betaine, including TG(18:4/18:2/22:6), TG(20:5/18:2/22:6), DG(18:3/22:6), DG(20:4/22:6), OAHFA(22:6/22:5), FA(22:6), PE(18:2e/22:6), PE(18:1e/22:6), PE(16:1e/22:6), PC(18:1/22:6), and PC(16:1/22:6), displayed significantly positive correlations with PYY and CCK. CCK, a hormone secreted by intestinal I-cells that stimulates bile and pancreatic lipase release, plays a crucial role in regulating food consumption, energy storage, and maintaining glucose balance. Wanxiu Cao et al. found that DHA-riched triacylglycerol (DHA-TG) specifically enhanced CCK expression, leading to decreased food intake and modulation of neuropeptide expression in rats fed a HFD (67). Like CCK, PYY belongs to the family of glucagon-like peptides which help generate feelings of fullness, and the levels are lower in obese individuals (35, 68). These findings indicate that betaine may influence the secretion of intestinal hormones through modulation of the synthesis of specific fatty acids. However, further mechanistic investigations (e.g., utilizing receptor antagonists) are warranted to elucidate the precise mechanisms by which betaine affects intestinal hormone regulation.

In short, these results from our comprehensive omics-based analysis suggested that betaine may alleviate obesity resulting from HFD by regulating lipid metabolism and gastrointestinal hormones, which is probably associated with the modulation of gut microbiota and alterations in SCFA species. Nevertheless, the precise functional roles of these pathways remain to be thoroughly elucidated through further investigation. A limitation of the current work is the absence of deeper molecular and functional confirmation of key omics findings. In further study, independent verification of core DEGs using qPCR and Western blotting at both mRNA and protein levels should be conducted to robustly confirm our findings. Meanwhile, considering that our research has identified the impact of betaine on the synthesis of DHA-rich phospholipids, the enzyme activity assay should be conducted to evaluate the regulatory effects of these specific lipid molecules on key metabolic enzymes or signaling pathways. Additionally, to further elucidate the mechanism by which DHA-rich phospholipids promotes thermogenesis, the mitochondrial respiratory function of brown and beige fat cells or tissues should be directly measured using high-resolution respirometry techniques (such as the Seahorse analyzer). This will provide direct physiological evidence for the regulation of mitochondrial biogenesis and activity by betaine.

## 5 Conclusion

In summary, our results revealed that betaine exhibited obesity-alleviating effects including the reduction of fat accumulation and weight gain, amelioration of dyslipidemia,enhancement of lipolysis, and acceleration of polyunsaturated fatty acid metabolism. Correlation analysis of multi-omics techniques suggest that betaine intervention could cause significant correlations between certain gut microbiota (e.g., *Lactobacillus*, *Ruminococcus*), certain lipid species (e.g., DHA-TG, DHA-PE), genes and pathways involving glycerophospholipid synthesis and lipid metabolism, and gastrointestinal hormones (e.g., CCK, PYY), which may be specific biomarkers of betaine to reduce HFD-induced obesity. It should be recognized that the mechanism by which betaine alleviates obesity-related metabolic disorders is complex and may be related to gut microbiome regulation and SCFA metabolism. These results suggest that betaine can regulate energy metabolism and gastrointestinal hormones by regulating fatty acid metabolism and promoting DHA fatty acid biosynthesis which may provide fresh perspectives on elucidating the underlying mechanisms by which betaine prevents obesity. Considering that betaine can be found naturally in many foods, it has the potential to become a new type of daily food to reduce HFD-induced obesity.

## Data Availability

The original contributions presented in the study are publicly available. The data presented in the study are deposited in the SRA repository, accession number: PRJNA1276250, PRJNA1277164 and in the OMIX, China National Center for Bioinformation: https://ngdc.cncb.ac.cn/omix/release/OMIX010582.

## Footnotes

^1^ https://cloud.majorbio.com

## References

[1] Wood B Garton K Milsom P Baker P Anastasiou K Clark J. Using a systems thinking approach to map the global rise of ultra-processed foods in population diets. Obes Rev. (2024) 26:e13877. doi: 10.1111/obr.13877, PMID: 39627009 PMC11884965

[2] Hojs R Ekart R Bevc S Vodošek Hojs N. Chronic kidney disease and obesity. Nephron. (2023) 147:660–4. doi: 10.1159/000531379, PMID: 37271131

[3] Nedunchezhiyan U Varughese I Sun AR Wu X Crawford R Prasadam I. Obesity, inflammation, and immune system in osteoarthritis. Front Immunol. (2022) 13:750. doi: 10.3389/fimmu.2022.907750 PMC9289681 35860250

[4] Nussbaumerova B Rosolova H. Obesity and dyslipidemia. Curr Atheroscler Rep. (2023) 25:947–55. doi: 10.1007/s11883-023-01167-2, PMID: 37979064

[5] Piché M-E Tchernof A Després J-P. Obesity phenotypes, diabetes, and cardiovascular diseases. Circ Res. (2020) 126:1477–500. doi: 10.1161/CIRCRESAHA.120.316101, PMID: 32437302

[6] Polyzos SA Kountouras J Mantzoros CS. Obesity and nonalcoholic fatty liver disease: from pathophysiology to therapeutics. Metabolism. (2019) 92:82–97. doi: 10.1016/j.metabol.2018.11.014, PMID: 30502373

[7] Avgerinos KI Spyrou N Mantzoros CS Dalamaga M. Obesity and cancer risk: emerging biological mechanisms and perspectives. Metabolism. (2019) 92:121–35. doi: 10.1016/j.metabol.2018.11.001, PMID: 30445141

[8] Chew NWS Ng CH Tan DJH Kong G Lin C Chin YH. The global burden of metabolic disease: data from 2000 to 2019. Cell Metab. (2023) 35:414–428.e3. doi: 10.1016/j.cmet.2023.02.003, PMID: 36889281

[9] Müller TD Blüher M Tschöp MH DiMarchi RD. Anti-obesity drug discovery: advances and challenges. Nat Rev Drug Discov. (2022) 21:201–23. doi: 10.1038/s41573-021-00337-8, PMID: 34815532 PMC8609996

[10] Góralczyk-Bińkowska A Szmajda-Krygier D Kozłowska E. The microbiota-gut-brain Axis in psychiatric disorders. Int J Mol Sci. (2022) 23:11245. doi: 10.3390/ijms231911245, PMID: 36232548 PMC9570195

[11] Li T-T Chen X Huo D Arifuzzaman M Qiao S Jin W-B. Microbiota metabolism of intestinal amino acids impacts host nutrient homeostasis and physiology. Cell Host Microbe. (2024) 32:661–675.e10. doi: 10.1016/j.chom.2024.04.004 38657606 PMC11636940

[12] Matson V Chervin CS Gajewski TF. Cancer and the microbiome-influence of the commensal microbiota on Cancer, immune responses, and immunotherapy. Gastroenterology. (2021) 160:600–13. doi: 10.1053/j.gastro.2020.11.041, PMID: 33253684 PMC8409239

[13] Jiao W Sang Y Wang X Wang S. Metabonomics and the gut microbiome analysis of the effect of 6-shogaol on improving obesity. Food Chem. (2023) 404:134734. doi: 10.1016/j.foodchem.2022.134734, PMID: 36327507

[14] Cuevas-Sierra A Ramos-Lopez O Riezu-Boj JI Milagro FI Martinez JA. Diet, gut microbiota, and obesity: links with host genetics and epigenetics and potential applications. Adv Nutr. (2019) 10:S17–30. doi: 10.1093/advances/nmy078, PMID: 30721960 PMC6363528

[15] Yi E-Y Kim Y-J. Betaine inhibits in vitro and in vivo angiogenesis through suppression of the NF-κB and Akt signaling pathways. Int J Oncol. (2012) 41:1879–85. doi: 10.3892/ijo.2012.1616, PMID: 22940742

[16] Craig SAS. Betaine in human nutrition. Am J Clin Nutr. (2004) 80:539–49. doi: 10.1093/ajcn/80.3.539, PMID: 15321791

[17] Hassanpour S Rezaei H Razavi SM. Anti-nociceptive and antioxidant activity of betaine on formalin- and writhing tests induced pain in mice. Behav Brain Res. (2020) 390:112699. doi: 10.1016/j.bbr.2020.112699, PMID: 32417277

[18] Jurko L Makuc D Štern A Plavec J Žegura B Bošković P. Cytotoxicity and antibacterial efficacy of betaine- and Choline-substituted polymers. ACS Appl Polym Mater. (2023) 5:5270–9. doi: 10.1021/acsapm.3c00691, PMID: 37469879 PMC10353005

[19] Zhao G He F Wu C Li P Li N Deng J. Betaine in inflammation: mechanistic aspects and applications. Front Immunol. (2018) 9:1070. doi: 10.3389/fimmu.2018.01070, PMID: 29881379 PMC5976740

[20] Du J Zhang P Luo J Shen L Zhang S Gu H. Dietary betaine prevents obesity through gut microbiota-drived microRNA-378a family. Gut Microbes. (2021) 13:1–19. doi: 10.1080/19490976.2020.1862612, PMID: 33550882 PMC7889173

[21] Choline SJ. Betaine and methionine interactions in chickens, pigs and fish (including crustaceans). World Poult Sci J. (1999) 55:353–74. doi: 10.1079/WPS19990025

[22] Li Q Qu M Wang N Wang L Fan G Yang C. Betaine protects rats against ischemia/reperfusion injury-induced brain damage. J Neurophysiol. (2022) 127:444–51. doi: 10.1152/jn.00400.2021, PMID: 35020521

[23] Fu R Wang Q Kong C Liu K Si H Sui S. Mechanism of action and the uses betaine in pig production. J Anim Physiol Anim Nutr (Berl). (2022) 106:528–36. doi: 10.1111/jpn.13633, PMID: 34486782

[24] Guo F Jing M Zhang A Yu Y Gao P Wang Q. Betaine alleviates LPS-induced chicken skeletal muscle inflammation with the epigenetic modulation of the TLR4 gene. Animals (Basel). (2022) 12:1899. doi: 10.3390/ani12151899, PMID: 35892549 PMC9330308

[25] Ashtary-Larky D Bagheri R Tinsley GM Asbaghi O Salehpour S Kashkooli S. Betaine supplementation fails to improve body composition: a systematic review and meta-analysis. Br J Nutr. (2022) 128:975–88. doi: 10.1017/S0007114521004062, PMID: 34743773

[26] Chen Y Liu Y Liu Y Wang X Guan K Zhu H. Higher serum concentrations of betaine rather than choline is associated with better profiles of DXA-derived body fat and fat distribution in Chinese adults. Int J Obes. (2015) 39:465–71. doi: 10.1038/ijo.2014.158, PMID: 25152241

[27] Li B Dewey CN. RSEM: accurate transcript quantification from RNA-Seq data with or without a reference genome. BMC Bioinformatics. (2011) 12:323. doi: 10.1186/1471-2105-12-323, PMID: 21816040 PMC3163565

[28] Çakır I Hadley CK Pan PL Bagchi RA Ghamari-Langroudi M Porter DT. Histone deacetylase 6 inhibition restores leptin sensitivity and reduces obesity. Nat Metab. (2022) 4:44–59. doi: 10.1038/s42255-021-00515-3, PMID: 35039672 PMC8892841

[29] Fan C Hu H Huang X Su D Huang F Zhuo Z. Betaine supplementation causes an increase in fatty acid oxidation and carbohydrate metabolism in livers of mice fed a high-fat diet: a proteomic analysis. Food Secur. (2022) 11:881. doi: 10.3390/foods11060881, PMID: 35327303 PMC8949908

[30] Zeisel SH. Betaine supplementation and blood lipids: fact or artifact? Nutr Rev. (2006) 64:77–9. doi: 10.1301/nr.2006.feb.77-79, PMID: 16536184

[31] Huang QC Xu ZR Han XY Li WF. Effect of dietary betaine supplementation on lipogenic enzyme activities and fatty acid synthase mRNA expression in finishing pigs. Anim Feed Sci Technol. (2008) 140:365–75. doi: 10.1016/j.anifeedsci.2007.03.007

[32] Martins JM Neves JA Freitas A Tirapicos JL. Effect of long-term betaine supplementation on chemical and physical characteristics of three muscles from the Alentejano pig. J Sci Food Agric. (2012) 92:2122–7. doi: 10.1002/jsfa.5595, PMID: 22307525

[33] Szkudelska K. Betaine supplementation to rats alleviates disturbances induced by high-fat diet: pleiotropic effects in model of type 2 diabetes. J Physiol Pharmacol. (2022) 72:11. doi: 10.26402/jpp.2021.5.11 35288478

[34] Álvarez-Arraño V Martín-Peláez S. Effects of probiotics and Synbiotics on weight loss in subjects with overweight or obesity: a systematic review. Nutrients. (2021) 13:3627. doi: 10.3390/nu13103627, PMID: 34684633 PMC8540110

[35] Gomes AC Hoffmann C Mota JF. The human gut microbiota: metabolism and perspective in obesity. Gut Microbes. (2018) 9:1–18. doi: 10.1080/19490976.2018.1465157, PMID: 29667480 PMC6219651

[36] Kang Y Kang X Yang H Liu H Yang X Liu Q. *Lactobacillus acidophilus* ameliorates obesity in mice through modulation of gut microbiota dysbiosis and intestinal permeability. Pharmacol Res. (2022) 175:106020. doi: 10.1016/j.phrs.2021.106020, PMID: 34896249

[37] Liu Y-W Liong M-T Tsai Y-C. New perspectives of *Lactobacillus plantarum* as a probiotic: the gut-heart-brain axis. J Microbiol. (2018) 56:601–13. doi: 10.1007/s12275-018-8079-2, PMID: 30141154

[38] Ma L Ni Y Wang Z Tu W Ni L Zhuge F. Spermidine improves gut barrier integrity and gut microbiota function in diet-induced obese mice. Gut Microbes. (2020) 12:1832857–19. doi: 10.1080/19490976.2020.1832857, PMID: 33151120 PMC7668533

[39] Feng J Ma H Huang Y Li J Li W. Ruminococcaceae_UCG-013 promotes obesity resistance in mice. Biomedicines. (2022) 10:3272. doi: 10.3390/biomedicines10123272, PMID: 36552029 PMC9776008

[40] Tamanai-Shacoori Z Smida I Bousarghin L Loreal O Meuric V Fong SB. Roseburia spp.: a marker of health? Future Microbiol. (2017) 12:157–70. doi: 10.2217/fmb-2016-0130, PMID: 28139139

[41] Yang J Li Y Wen Z Liu W Meng L Huang H. Oscillospira - a candidate for the next-generation probiotics. Gut Microbes. (2021) 13:1987783. doi: 10.1080/19490976.2021.1987783, PMID: 34693878 PMC8547878

[42] Perry RJ Peng L Barry NA Cline GW Zhang D Cardone RL. Acetate mediates a microbiome–brain–β-cell axis to promote metabolic syndrome. Nature. (2016) 534:213–7. doi: 10.1038/nature18309, PMID: 27279214 PMC4922538

[43] Zeng Q Li D He Y Li Y Yang Z Zhao X. Discrepant gut microbiota markers for the classification of obesity-related metabolic abnormalities. Sci Rep. (2019) 9:13424. doi: 10.1038/s41598-019-49462-w, PMID: 31530820 PMC6748942

[44] Park B-O Kim SH Kong GY Kim DH Kwon MS Lee SU. Selective novel inverse agonists for human GPR43 augment GLP-1 secretion. Eur J Pharmacol. (2016) 771:1–9. doi: 10.1016/j.ejphar.2015.12.010, PMID: 26683635

[45] Truax AD Chen L Tam JW Cheng N Guo H Koblansky AA. The inhibitory innate immune sensor NLRP12 maintains a threshold against obesity by regulating gut microbiota homeostasis. Cell Host Microbe. (2018) 24:364–378.e6. doi: 10.1016/j.chom.2018.08.009, PMID: 30212649 PMC6161752

[46] Zaky A Glastras SJ Wong MYW Pollock CA Saad S. The role of the gut microbiome in diabetes and obesity-related kidney disease. Int J Mol Sci. (2021) 22:9641. doi: 10.3390/ijms22179641, PMID: 34502562 PMC8431784

[47] Coppola S Avagliano C Calignano A Berni Canani R. The protective role of butyrate against obesity and obesity-related diseases. Molecules. (2021) 26:682. doi: 10.3390/molecules26030682, PMID: 33525625 PMC7865491

[48] Wu F Mao L Zhang Y Chen X Zhuang P Wang W. Individual SFA intake and risk of overweight/obesity: findings from a population-based nationwide cohort study. Br J Nutr. (2022) 128:75–83. doi: 10.1017/S0007114521002890, PMID: 34338170

[49] Morigny P Boucher J Arner P Langin D. Lipid and glucose metabolism in white adipocytes: pathways, dysfunction and therapeutics. Nat Rev Endocrinol. (2021) 17:276–95. doi: 10.1038/s41574-021-00471-8, PMID: 33627836

[50] Kawai T Autieri MV Scalia R. Adipose tissue inflammation and metabolic dysfunction in obesity. Am J Physiol Cell Physiol. (2021) 320:C375–91. doi: 10.1152/ajpcell.00379.2020, PMID: 33356944 PMC8294624

[51] Marcelin G Silveira ALM Martins LB Ferreira AV Clément K. Deciphering the cellular interplays underlying obesity-induced adipose tissue fibrosis. J Clin Invest. (2019) 129:4032–40. doi: 10.1172/JCI129192, PMID: 31498150 PMC6763252

[52] Casati S Giannasi C Niada S Bergamaschi RF Orioli M Brini AT. Bioactive lipids in MSCs biology: state of the art and role in inflammation. Int J Mol Sci. (2021) 22:1481. doi: 10.3390/ijms22031481, PMID: 33540695 PMC7867257

[53] Zhang F Chen Z Wu D Tian L Chen Q Ye Y. Recombinant human GLP-1 beinaglutide regulates lipid metabolism of adipose tissues in diet-induced obese mice. iScience. (2021) 24:103382. doi: 10.1016/j.isci.2021.103382, PMID: 34841227 PMC8605346

[54] Hou B Zhao Y He P Xu C Ma P Lam SM. Targeted lipidomics and transcriptomics profiling reveal the heterogeneity of visceral and subcutaneous white adipose tissue. Life Sci. (2020) 245:117352. doi: 10.1016/j.lfs.2020.117352, PMID: 32006527 PMC7988272

[55] Davies BSJ Beigneux AP Barnes RH Tu Y Gin P Weinstein MM. GPIHBP1 is responsible for the entry of lipoprotein lipase into capillaries. Cell Metab. (2010) 12:42–52. doi: 10.1016/j.cmet.2010.04.016, PMID: 20620994 PMC2913606

[56] Gimeno RE. Fatty acid transport proteins. Curr Opin Lipidol. (2007) 18:271–6. doi: 10.1097/MOL.0b013e3281338558, PMID: 17495600

[57] Cao Y Wang S Liu S Wang Y Jin H Ma H. Effects of long-chain fatty acyl-CoA Synthetase 1 on diglyceride synthesis and arachidonic acid metabolism in sheep adipocytes. Int J Mol Sci. (2020) 21:2044. doi: 10.3390/ijms21062044, PMID: 32192050 PMC7139739

[58] Aslibekyan S Jensen MK Campos H Linkletter CD Loucks EB Ordovas JM. Genetic variation in fatty acid elongases is not associated with intermediate cardiovascular phenotypes or myocardial infarction. Eur J Clin Nutr. (2012) 66:353–9. doi: 10.1038/ejcn.2012.2, PMID: 22293571 PMC3806713

[59] Xiao J Wang W-X. Genome-wide identification and expression profile of Elovl genes in threadfin fish Eleutheronema. Sci Rep. (2023) 13:1080. doi: 10.1038/s41598-023-28342-4, PMID: 36658196 PMC9852283

[60] Lager I Yilmaz JL Zhou X-R Jasieniecka K Kazachkov M Wang P. Plant acyl-CoA:lysophosphatidylcholine acyltransferases (LPCATs) have different specificities in their forward and reverse reactions. J Biol Chem. (2013) 288:36902–14. doi: 10.1074/jbc.M113.521815, PMID: 24189065 PMC3873549

[61] Tang X Brindley DN. Lipid phosphate phosphatases and cancer. Biomol Ther. (2020) 10:1263. doi: 10.3390/biom10091263, PMID: 32887262 PMC7564803

[62] Opreanu M Tikhonenko M Bozack S Lydic TA Reid GE McSorley KM. The unconventional role of acid sphingomyelinase in regulation of retinal microangiopathy in diabetic human and animal models. Diabetes. (2011) 60:2370–8. doi: 10.2337/db10-0550, PMID: 21771974 PMC3161322

[63] Ding L Yang J Guo H Cong P Xu J Xue C. Dietary Eicosapentaenoic acid containing Phosphoethanolamine Plasmalogens remodels the Lipidome of White adipose tissue and suppresses high-fat diet induced obesity in mice. Mol Nutr Food Res. (2023) 67:e2200321. doi: 10.1002/mnfr.202200321, PMID: 37439463

[64] Zhou M Ding L Wen M Che H Huang J Zhang T. Mechanisms of DHA-enriched phospholipids in improving cognitive deficits in aged SAMP8 mice with high-fat diet. J Nutr Biochem. (2018) 59:64–75. doi: 10.1016/j.jnutbio.2018.05.009, PMID: 29986309

[65] Jansen KM Dahdah N Gama-Perez P Schots PC Larsen TS Garcia-Roves PM. Impact of GLP-1 receptor agonist versus omega-3 fatty acids supplement on obesity-induced alterations of mitochondrial respiration. Front Endocrinol (Lausanne). (2023) 14:1098391. doi: 10.3389/fendo.2023.1098391, PMID: 37033212 PMC10076843

[66] Polley KR Kamal F Paton CM Cooper JA. Appetite responses to high-fat diets rich in mono-unsaturated versus poly-unsaturated fats. Appetite. (2019) 134:172–81. doi: 10.1016/j.appet.2018.12.008, PMID: 30550892

[67] Cao W Liu F Li RW Yang R Wang Y Xue C. Triacylglycerol rich in docosahexaenoic acid regulated appetite via the mediation of leptin and intestinal epithelial functions in high-fat, high-sugar diet-fed mice. J Nutr Biochem. (2022) 99:108856. doi: 10.1016/j.jnutbio.2021.108856, PMID: 34517098

[68] Batterham RL Cohen MA Ellis SM Le Roux CW Withers DJ Frost GS. Inhibition of food intake in obese subjects by peptide YY3-36. N Engl J Med. (2003) 349:941–8. doi: 10.1056/NEJMoa030204, PMID: 12954742

