# Supplementary material for: Betaine alleviates obesity-related metabolic disorders in rats: insights from microbiomes, lipidomics, and transcriptomics
Source: Front Nutr. 2025 Jul 10;12:1604801. doi: 10.3389/fnut.2025.1604801 (PMC12286965; doi:10.3389/fnut.2025.1604801)
Supplement: Supplementary file 1 [file Table_1.pdf]

Supplementary Table 1. The Mantel test analysis of differential genus (top 20) and SCFAs concentrations. Related to Figure 3M.

DIM 1	DIM2	Mantel r statistic	p_value	Number of permutations
HFD	Isohexanoic acid	0.44643	0.026389	999
HBE	Hexanoic acid	0.23929	0.131944	999
HBE	Isobutyric acid	-0.12143	0.656944	999
HFD	Propanoic acid	0.41786	0.086111	999
HBE	Acetic acid	0.51429	0.105556	999
HBE	Straight-chainfattyacids	0.75714	0.027778	999
HBE	Valeric acid	0.08214	0.379167	999
HBE	Isohexanoic acid	0.54643	0.154167	999
HBE	Branched-chainfattyacids	-0.18929	0.786111	999
HBE	Isovaleric acid	-0.17143	0.743056	999
HFD	Straight-chainfattyacids	-0.17857	0.709722	999
HBE	Butanoic acid	0.20714	0.283333	999
HFD	Isovaleric acid	-0.07857	0.518056	999
HBE	Propanoic acid	0.63929	0.104167	999
HFD	Hexanoic acid	-0.18214	0.791667	999
NCD	Acetic acid	-0.24643	0.709722	999
NCD	Straight-chainfattyacids	-0.01071	0.409722	999
NCD	Isohexanoic acid	-0.05	0.491667	999
HFD	Acetic acid	-0.18571	0.697222	999
NCD	Valeric acid	-0.175	0.698611	999
NCD	Propanoic acid	-0.33571	0.969444	999
HBE	TotalSCFA	0.75714	0.027778	999
HFD	Butanoic acid	-0.225	0.780556	999
NCD	Isovaleric acid	-0.06071	0.541667	999
NCD	Hexanoic acid	0.25714	0.216667	999
HFD	Isobutyric acid	-0.26071	0.804167	999
HFD	Branched-chainfattyacids	-0.16429	0.643056	999
NCD	Isobutyric acid	-0.23214	0.829167	999
NCD	TotalSCFA	-0.01071	0.431944	999
HFD	TotalSCFA	-0.17857	0.709722	999
NCD	Butanoic acid	-0.3	0.858333	999
NCD	Branched-chainfattyacids	-0.21786	0.827778	999
HFD	Valeric acid	-0.16071	0.636111	999
all	all	0.3483	0.007	999

Supplementary Table 2. The KEGG pathway joint analysis in transcriptome and metabolome. Related to Figure 7C.

Pathway ID	Pathway Description	First Category	Second Category	Metabolite number	*P* value (metabolite)	Gene number	*P* value (gene)
rno04931	Insulin resistance	Human Diseases	Endocrine and metabolic disease	1	**0.013***	21	**0.041***
rno04923	Regulation of lipolysis in adipocytes	Organismal Systems	Endocrine system	1	**0.010***	15	**0.013***
rno04723	Retrograde endocannabinoid signaling	Organismal Systems	Nervous system	4	**0.013***	22	0.451
rno05231	Choline metabolism in cancer	Human Diseases	Cancer: overview	7	**0.000***	18	0.098
rno05417	Lipid and atherosclerosis	Human Diseases	Cardiovascular disease	1	**0.010***	30	0.345
rno04714	Thermogenesis	Organismal Systems	Environmental adaptation	1	**0.016 ***	36	0.154
rno04975	Fat digestion and absorption	Organismal Systems	Digestive system	1	**0.009***	5	0.776
rno04977	Vitamin digestion and absorption	Organismal Systems	Digestive system	1	**0.026***	4	0.593
rno00561	Glycerolipid metabolism	Metabolism	Lipid metabolism	1	**0.026 ***	8	0.683
rno04979	Cholesterol metabolism	Organismal Systems	Digestive system	1	**0.007***	11	0.052
rno00564	Glycerophospholipid metabolism	Metabolism	Lipid metabolism	7	**<0.001***	7	0.996
rno04910	Insulin signaling pathway	Organismal Systems	Endocrine system	0	-	25	**0.048***
rno04921	Oxytocin signaling pathway	Organismal Systems	Endocrine system	0	-	31	**0.045***
rno05218	Melanoma	Human Diseases	Cancer: specific types	0	-	16	**0.044***
rno03460	Fanconi anemia pathway	Genetic Information Processing	Replication and repair	0	-	13	**0.044***
rno05100	Bacterial invasion of epithelial cells	Human Diseases	Infectious disease: bacterial	0	-	17	**0.044***
rno05017	Spinocerebellar ataxia	Human Diseases	Neurodegenerative disease	0	-	24	**0.044***
rno04380	Osteoclast differentiation	Organismal Systems	Development and regeneration	0	-	24	**0.044***
rno03015	mRNA surveillance pathway	Genetic Information Processing	Translation	0	-	19	**0.043***
rno04933	AGE-RAGE signaling pathway in diabetic complications	Human Diseases	Endocrine and metabolic disease	0	-	22	**0.040***
rno04514	Cell adhesion molecules	Environmental Information Processing	Signaling molecules and interaction	0	-	32	**0.039***

Continued Table 2. The KEGG pathway joint analysis in transcriptome and metabolome. Related to Figure 7C.

Pathway ID	Pathway Description	First Category	Second Category	Metabolite number	*P* value (metabolite)	Gene number	*P* value (gene)
rno04142	Lysosome	Cellular Processes	Transport and catabolism	0	-	24	**0.039***
rno03430	Mismatch repair	Genetic Information Processing	Replication and repair	0	-	7	**0.038***
rno04932	Non-alcoholic fatty liver disease	Human Diseases	Endocrine and metabolic disease	0	-	29	**0.038***
rno04115	p53 signaling pathway	Cellular Processes	Cell growth and death	0	-	15	**0.037***
rno05216	Thyroid cancer	Human Diseases	Cancer: specific types	0	-	9	**0.037***
rno05152	Tuberculosis	Human Diseases	Infectious disease: bacterial	0	-	30	**0.037***
rno04926	Relaxin signaling pathway	Organismal Systems	Endocrine system	0	-	26	**0.037***
rno05213	Endometrial cancer	Human Diseases	Cancer: specific types	0	-	13	**0.036***
rno05414	Dilated cardiomyopathy	Human Diseases	Cardiovascular disease	0	-	21	**0.036***
rno05142	Chagas disease	Human Diseases	Infectious disease: parasitic	0	-	22	**0.035***
rno00514	Other types of O-glycan biosynthesis	Metabolism	Glycan biosynthesis and metabolism	0	-	12	**0.035***
rno04935	Growth hormone synthesis, secretion and action	Organismal Systems	Endocrine system	0	-	26	**0.032***
rno05214	Glioma	Human Diseases	Cancer: specific types	0	-	17	**0.032***
rno04360	Axon guidance	Organismal Systems	Development and regeneration	0	-	35	**0.031***
rno03420	Nucleotide excision repair	Genetic Information Processing	Replication and repair	0	-	12	**0.028***
rno03020	RNA polymerase	Genetic Information Processing	Transcription	0	-	9	**0.028***
rno04924	Renin secretion	Organismal Systems	Endocrine system	0	-	18	**0.027***
rno05200	Pathways in cancer	Human Diseases	Cancer: overview	0	-	93	**0.025***
rno04720	Long-term potentiation	Organismal Systems	Nervous system	0	-	14	**0.025***

Continued Table 2. The KEGG pathway joint analysis in transcriptome and metabolome.

Pathway ID	Pathway Description	First Category	Second Category	Metabolite number	*P* value (metabolite)	Gene number	*P* value (gene)
rno04150	mTOR signaling pathway	Environmental Information Processing	Signal transduction	0	-	28	**0.024***
rno04022	cGMP-PKG signaling pathway	Environmental Information Processing	Signal transduction	0	-	37	**0.024***
rno00480	Glutathione metabolism	Metabolism	Metabolism of other amino acids	0	-	13	**0.023***
rno04670	Leukocyte transendothelial migration	Organismal Systems	Immune system	0	-	25	**0.022***
rno04024	cAMP signaling pathway	Environmental Information Processing	Signal transduction	0	-	44	**0.021***
rno05418	Fluid shear stress and atherosclerosis	Human Diseases	Cardiovascular disease	0	-	29	**0.020***
rno05415	Diabetic cardiomyopathy	Human Diseases	Cardiovascular disease	0	-	39	**0.020***
rno04666	Fc gamma R-mediated phagocytosis	Organismal Systems	Immune system	0	-	21	**0.019***
rno03008	Ribosome biogenesis in eukaryotes	Genetic Information Processing	Translation	0	-	20	**0.019***
rno05212	Pancreatic cancer	Human Diseases	Cancer: specific types	0	-	17	**0.018***
rno01524	Platinum drug resistance	Human Diseases	Drug resistance: antineoplastic	0	-	17	**0.018***
rno04151	PI3K-Akt signaling pathway	Environmental Information Processing	Signal transduction	0	-	63	**0.016***
rno04962	Vasopressin-regulated water reabsorption	Organismal Systems	Excretory system	0	-	11	**0.015***
rno04625	C-type lectin receptor signaling pathway	Organismal Systems	Immune system	0	-	23	**0.014***

Continued Table 2. The KEGG pathway joint analysis in transcriptome and metabolome.

Pathway ID	Pathway Description	First Category	Second Category	Metabolite number	*P* value (metabolite)	Gene number	*P* value (gene)
rno04071	Sphingolipid signaling pathway	Environmental Information Processing	Signal transduction	0	-	26	**0.014***
rno04211	Longevity regulating pathway	Organismal Systems	Aging	0	-	21	**0.013***
rno04520	Adherens junction	Cellular Processes	Cellular community - eukaryotes	0	-	18	**0.012***
rno04810	Regulation of actin cytoskeleton	Cellular Processes	Cell motility	0	-	45	**0.012***
rno03040	Spliceosome	Genetic Information Processing	Transcription	0	-	26	**0.012***
rno05144	Malaria	Human Diseases	Infectious disease: parasitic	0	-	13	**0.011***
rno05210	Colorectal cancer	Human Diseases	Cancer: specific types	0	-	20	**0.011***
rno04072	Phospholipase D signaling pathway	Environmental Information Processing	Signal transduction	0	-	34	**0.011***
rno01521	EGFR tyrosine kinase inhibitor resistance	Human Diseases	Drug resistance: antineoplastic	0	-	19	**0.010***
rno05225	Hepatocellular carcinoma	Human Diseases	Cancer: specific types	0	-	36	**0.010***
rno04510	Focal adhesion	Cellular Processes	Cellular community - eukaryotes	0	-	43	**0.010***
rno04722	Neurotrophin signaling pathway	Organismal Systems	Nervous system	0	-	24	**0.010***
rno04650	Natural killer cell mediated cytotoxicity	Organismal Systems	Immune system	0	-	19	**0.008***
rno04919	Thyroid hormone signaling pathway	Organismal Systems	Endocrine system	0	-	28	**0.008***
rno04144	Endocytosis	Cellular Processes	Transport and catabolism	0	-	47	**0.008***
rno04662	B cell receptor signaling pathway	Organismal Systems	Immune system	0	-	20	**0.007***
rno05164	Influenza A	Human Diseases	Infectious disease: viral	0	-	32	**0.007***
rno04068	FoxO signaling pathway	Environmental Information Processing	Signal transduction	0	-	27	**0.007***

Continued Table 2. The KEGG pathway joint analysis in transcriptome and metabolome.

Pathway ID	Pathway Description	First Category	Second Category	Metabolite number	*P* value (metabolite)	Gene number	*P* value (gene)
rno05010	Alzheimer disease	Human Diseases	Neurodegenerative disease	0	-	68	**0.007***
rno04611	Platelet activation	Organismal Systems	Immune system	0	-	31	**0.006***
rno04218	Cellular senescence	Cellular Processes	Cell growth and death	0	-	38	**0.006***
rno03050	Proteasome	Genetic Information Processing	Folding, sorting and degradation	0	-	13	**0.004***
rno05135	Yersinia infection	Human Diseases	Infectious disease: bacterial	0	-	29	**0.004***
rno04640	Hematopoietic cell lineage	Organismal Systems	Immune system	0	-	21	**0.004***
rno04530	Tight junction	Cellular Processes	Cellular community - eukaryotes	0	-	35	**0.003***
rno04660	T cell receptor signaling pathway	Organismal Systems	Immune system	0	-	25	**0.003***
rno04066	HIF-1 signaling pathway	Environmental Information Processing	Signal transduction	0	-	24	**0.002***
rno05016	Huntington disease	Human Diseases	Neurodegenerative disease	0	-	58	**0.002***
rno04137	Mitophagy - animal	Cellular Processes	Transport and catabolism	0	-	20	**0.002***
rno05020	Prion disease	Human Diseases	Neurodegenerative disease	0	-	56	**0.001***
rno05014	Amyotrophic lateral sclerosis	Human Diseases	Neurodegenerative disease	0	-	67	**0.001***
rno05022	Pathways of neurodegeneration - multiple diseases	Human Diseases	Neurodegenerative disease	0	-	88	**0.001***
rno04136	Autophagy - other	Cellular Processes	Transport and catabolism	0	-	10	**0.001***
rno05205	Proteoglycans in cancer	Human Diseases	Cancer: overview	0	-	46	**0.001***
rno04010	MAPK signaling pathway	Environmental Information Processing	Signal transduction	0	-	60	**0.001***
rno04216	Ferroptosis	Cellular Processes	Cell growth and death	0	-	13	**0.000***
rno04210	Apoptosis	Cellular Processes	Cell growth and death	0	-	33	**0.000***

Continued Table 2. The KEGG pathway joint analysis in transcriptome and metabolome.

Pathway ID	Pathway Description	First Category	Second Category	Metabolite number	*P* value (metabolite)	Gene number	*P* value (gene)
rno04140	Autophagy - animal	Cellular Processes	Transport and catabolism	0	-	35	**0.000***
rno05235	PD-L1 expression and PD-1 checkpoint pathway in cancer	Human Diseases	Cancer: overview	0	-	24	**0.000**
rno05012	Parkinson disease	Human Diseases	Neurodegenerative disease	0	-	57	**0.000***
rno05132	Salmonella infection	Human Diseases	Infectious disease: bacterial	0	-	47	**0.000***
rno04015	Rap1 signaling pathway	Environmental Information Processing	Signal transduction	0	-	54	**0.000***
rno05171	Coronavirus disease - COVID-19	Human Diseases	Infectious disease: viral	0	-	69	**0.000***
rno03010	Ribosome	Genetic Information Processing	Translation	0	-	58	**0.000***
rno00591	Linoleic acid metabolism	Metabolism	Lipid metabolism	4	0.019	0	-
rno00592	alpha-Linolenic acid metabolism	Metabolism	Lipid metabolism	4	0.030	0	-

Supplementary Table 3：The R of Spearman’s correlation analysis among obesity-related biochemical indexes, gut microbiota, SCFA, endocrine hormone, and differential metabolites.Related to Fig. 10A.

R	Weight	ALT	AST	CHO	TG	HDL	LDL	GLP-1	PYY	CCK	INS	GAS	MTL
Weight	1.00	0.25	0.24	0.55	0.74	-0.49	0.50	-0.09	-0.18	-0.52	0.10	0.15	-0.17
ALT	0.25	1.00	0.79	0.12	0.14	-0.13	0.45	0.18	0.11	-0.31	-0.41	-0.26	-0.04
AST	0.24	0.79	1.00	0.13	0.21	-0.27	0.40	0.06	0.04	-0.10	-0.29	-0.39	-0.36
CHO	0.55	0.12	0.13	1.00	0.46	-0.31	0.44	0.32	0.27	-0.52	0.13	-0.08	-0.37
TG	0.74	0.14	0.21	0.46	1.00	-0.31	0.68	-0.26	-0.28	-0.38	-0.08	-0.15	-0.37
HDL	-0.49	-0.13	-0.27	-0.31	-0.31	1.00	-0.09	0.03	0.38	0.16	0.16	-0.19	-0.02
LDL	0.50	0.45	0.40	0.44	0.68	-0.09	1.00	0.01	0.02	-0.53	-0.13	-0.15	-0.21
GLP-1	-0.09	0.18	0.06	0.32	-0.26	0.03	0.01	1.00	0.22	-0.11	-0.04	-0.37	-0.27
PYY	-0.18	0.11	0.04	0.27	-0.28	0.38	0.02	0.22	1.00	0.07	0.38	-0.04	0.00
CCK	-0.52	-0.31	-0.10	-0.52	-0.38	0.16	-0.53	-0.11	0.07	1.00	-0.17	-0.15	-0.26
INS	0.10	-0.41	-0.29	0.13	-0.08	0.16	-0.13	-0.04	0.38	-0.17	1.00	0.35	0.21
GAS	0.15	-0.26	-0.39	-0.08	-0.15	-0.19	-0.15	-0.37	-0.04	-0.15	0.35	1.00	0.64
MTL	-0.17	-0.04	-0.36	-0.37	-0.37	-0.02	-0.21	-0.27	0.00	-0.26	0.21	0.64	1.00
Acetic acid	-0.20	0.10	-0.06	-0.05	-0.38	0.67	0.05	0.10	0.62	-0.01	0.33	0.16	0.18
Propanoic acid	0.10	0.20	0.23	0.23	-0.16	0.24	0.11	0.31	0.50	0.02	0.25	0.12	-0.21
Isobutyric acid	-0.20	-0.25	-0.07	-0.15	-0.38	0.21	-0.25	0.45	0.24	0.47	0.31	-0.03	-0.16
Butanoic acid	-0.18	-0.09	0.04	0.11	-0.40	0.36	-0.20	0.17	0.32	0.22	0.29	0.12	-0.10
Isovaleric acid	-0.24	-0.19	-0.03	-0.31	-0.42	0.26	-0.26	0.37	0.15	0.47	0.20	-0.03	-0.08
Valeric acid	-0.10	0.06	0.08	-0.14	-0.44	0.26	-0.24	0.50	0.27	0.26	0.17	0.11	-0.03
Isohexanoic acid	-0.74	-0.49	-0.44	-0.68	-0.58	0.23	-0.56	0.01	-0.16	0.44	-0.04	0.04	0.37
Hexanoic acid	-0.64	-0.57	-0.51	-0.55	-0.57	0.43	-0.59	-0.05	0.08	0.34	0.37	0.24	0.42
Straight-chainfattyacids	-0.20	0.07	-0.03	-0.01	-0.41	0.63	0.00	0.14	0.60	0.05	0.34	0.17	0.11
Branched-chainfattyacids	-0.26	-0.25	-0.08	-0.25	-0.42	0.24	-0.29	0.41	0.19	0.49	0.26	-0.03	-0.10
TotalSCFA	-0.20	0.06	-0.03	-0.02	-0.42	0.63	-0.01	0.16	0.59	0.07	0.35	0.17	0.11
g__Blautia	0.46	0.16	0.35	0.28	0.67	-0.65	0.55	-0.33	-0.47	-0.29	-0.31	0.03	-0.19
g__Romboutsia	0.37	0.29	0.24	-0.01	0.35	-0.50	0.13	0.19	-0.52	-0.19	-0.31	0.11	-0.05
g__Lachnoclostridium	0.63	0.48	0.41	0.14	0.57	-0.60	0.42	0.11	-0.58	-0.40	-0.30	0.02	-0.09

Continued Table 3：The R of Spearman's correlation analysis among obesity-related biochemical indexes, gut microbiota, SCFA, endocrine hormone, and differential metabolites.

R	Weight	ALT	AST	CHO	TG	HDL	LDL	GLP-1	PYY	CCK	INS	GAS	MTL
g__Staphylococcus	0.49	0.34	0.26	0.07	0.48	-0.55	0.41	0.17	-0.59	-0.41	-0.26	0.05	-0.02
g__unclassified_f__Oscillospiraceae	-0.63	-0.13	-0.47	-0.44	-0.64	0.60	-0.32	0.05	0.26	0.06	0.13	0.21	0.65
g__Ruminococcus	-0.57	-0.19	-0.28	-0.36	-0.61	0.46	-0.37	0.09	0.51	0.40	0.09	-0.11	0.18
g__Lactobacillus	-0.58	-0.46	-0.45	-0.49	-0.45	0.69	-0.46	-0.11	0.15	0.23	0.31	-0.07	0.26
g__norank_f__Muribaculaceae	-0.08	-0.08	-0.19	0.23	-0.37	0.41	-0.07	0.16	0.67	-0.02	0.58	0.21	0.03
g__Lachnospiraceae_NK4A136_group	0.05	0.18	0.13	0.27	-0.13	0.47	0.30	0.15	0.35	-0.12	0.13	0.03	-0.12
g__norank_o__Clostridia_UCG-014	-0.58	-0.24	-0.45	-0.22	-0.47	0.41	-0.36	0.09	0.31	0.56	-0.14	0.08	-0.02
Ehhadh	-0.19	0.48	0.38	0.28	-0.29	0.15	0.10	0.24	0.52	0.11	-0.16	-0.20	-0.28
Acox1	0.04	0.08	0.14	0.44	0.22	0.18	0.20	-0.05	0.23	0.17	-0.01	-0.28	-0.48
Acsl1	0.15	0.17	0.10	0.58	0.09	0.17	0.23	0.13	0.39	-0.03	0.15	-0.08	-0.35
Fabp4	-0.17	-0.01	-0.09	-0.25	-0.32	-0.28	-0.31	-0.17	-0.33	-0.08	-0.12	0.28	0.53
Lpl	-0.16	-0.10	-0.17	0.45	-0.09	0.31	-0.07	0.11	0.46	0.18	0.18	-0.05	-0.34
Pparg	0.21	0.27	0.29	0.55	0.34	0.14	0.41	0.07	0.40	-0.03	0.11	-0.31	-0.44
Ndufaf1	0.07	0.20	0.09	0.38	0.12	0.33	0.22	-0.09	0.31	-0.02	0.05	-0.12	-0.27
Ndufs1	-0.21	-0.01	-0.03	0.31	-0.03	0.52	0.13	0.06	0.50	0.19	0.15	-0.15	-0.38
Klhl13	-0.72	-0.34	-0.28	-0.33	-0.45	0.58	-0.39	-0.02	0.31	0.56	0.03	-0.27	-0.18
Prdm16	-0.39	-0.35	-0.33	-0.20	-0.16	0.54	-0.10	0.16	0.06	0.34	-0.05	-0.30	-0.24
Elovl2	-0.44	-0.54	-0.52	-0.51	-0.45	0.26	-0.41	-0.31	0.08	0.49	0.25	0.58	0.42
Plpp3	-0.59	-0.30	-0.13	-0.48	-0.38	0.41	-0.32	-0.02	0.24	0.64	0.09	-0.32	-0.18
Lpcat3	0.48	0.35	0.36	0.44	0.30	-0.25	0.31	0.17	0.14	-0.02	-0.09	-0.04	-0.42
Pld4	0.23	0.48	0.36	0.32	-0.03	0.21	0.40	0.30	0.51	-0.26	0.11	0.07	-0.15
TG(18:4/18:2/22:6)	-0.79	-0.37	-0.38	-0.53	-0.63	0.51	-0.52	-0.20	0.26	0.46	0.17	0.10	0.35
TG(20:5/18:2/22:6)	-0.84	-0.22	-0.32	-0.49	-0.80	0.64	-0.54	0.11	0.45	0.40	0.14	0.05	0.32
DG(18:3/22:6)	-0.80	-0.42	-0.51	-0.52	-0.70	0.65	-0.49	0.08	0.28	0.35	0.14	0.07	0.34
DG(20:4/22:6)	-0.74	-0.36	-0.45	-0.37	-0.68	0.64	-0.47	0.21	0.47	0.36	0.19	0.01	0.29
OAHFA(22:6/22:5)	-0.79	-0.39	-0.44	-0.47	-0.65	0.45	-0.53	-0.02	0.33	0.39	0.24	0.20	0.38
FA(22:6)	-0.79	-0.42	-0.45	-0.56	-0.60	0.42	-0.53	-0.07	0.23	0.40	0.17	0.16	0.41
PE(18:2e/22:6)	-0.71	-0.31	-0.44	-0.32	-0.72	0.66	-0.53	0.20	0.56	0.37	0.29	0.01	0.22

Continued Table 3：The R of Spearman's correlation analysis among obesity-related biochemical indexes, gut microbiota, SCFA, endocrine hormone, and differential metabolites.

R	Weight	ALT	AST	CHO	TG	HDL	LDL	GLP-1	PYY	CCK	INS	GAS	MTL
PE(18:1e/22:6)	-0.70	-0.23	-0.33	-0.23	-0.67	0.69	-0.40	0.21	0.56	0.36	0.21	-0.08	0.05
PE(16:1e/22:6)	-0.79	-0.34	-0.44	-0.45	-0.70	0.69	-0.53	0.12	0.48	0.40	0.24	-0.01	0.27
PC(18:1/22:6)	-0.47	-0.31	-0.46	-0.09	-0.52	0.54	-0.43	0.18	0.51	0.26	0.23	0.00	0.08
PC(16:1/22:6)	-0.60	-0.16	-0.36	-0.39	-0.68	0.69	-0.47	0.24	0.53	0.36	0.20	-0.04	0.27

Continued Table 3：The R of Spearman's correlation analysis among obesity-related biochemical indexes, gut microbiota, SCFA, endocrine hormone, and differential metabolites.

R	Acetic acid	Propanoic acid	Isobutyric acid	Butanoic acid	Isovaleric acid	Valeric acid	Isohexanoic acid	Hexanoic acid	Straight-chainfattyacids	Branched-chainfattyacids	Total SCFA
Weight	-0.20	0.10	-0.20	-0.18	-0.24	-0.10	-0.74	-0.64	-0.20	-0.26	-0.20
ALT	0.10	0.20	-0.25	-0.09	-0.19	0.06	-0.49	-0.57	0.07	-0.25	0.06
AST	-0.06	0.23	-0.07	0.04	-0.03	0.08	-0.44	-0.51	-0.03	-0.08	-0.03
CHO	-0.05	0.23	-0.15	0.11	-0.31	-0.14	-0.68	-0.55	-0.01	-0.25	-0.02
TG	-0.38	-0.16	-0.38	-0.40	-0.42	-0.44	-0.58	-0.57	-0.41	-0.42	-0.42
HDL	0.67	0.24	0.21	0.36	0.26	0.26	0.23	0.43	0.63	0.24	0.63
LDL	0.05	0.11	-0.25	-0.20	-0.26	-0.24	-0.56	-0.59	0.00	-0.29	-0.01
GLP-1	0.10	0.31	0.45	0.17	0.37	0.50	0.01	-0.05	0.14	0.41	0.16
PYY	0.62	0.50	0.24	0.32	0.15	0.27	-0.16	0.08	0.60	0.19	0.59
CCK	-0.01	0.02	0.47	0.22	0.47	0.26	0.44	0.34	0.05	0.49	0.07
INS	0.33	0.25	0.31	0.29	0.20	0.17	-0.04	0.37	0.34	0.26	0.35
GAS	0.16	0.12	-0.03	0.12	-0.03	0.11	0.04	0.24	0.17	-0.03	0.17
MTL	0.18	-0.21	-0.16	-0.10	-0.08	-0.03	0.37	0.42	0.11	-0.10	0.11
Acetic acid	1.00	0.75	0.32	0.63	0.29	0.52	-0.12	0.15	0.98	0.29	0.98
Propanoic acid	0.75	1.00	0.37	0.63	0.25	0.62	-0.44	-0.19	0.81	0.29	0.81
Isobutyric acid	0.32	0.37	1.00	0.50	0.96	0.84	0.31	0.47	0.40	0.99	0.43
Butanoic acid	0.63	0.63	0.50	1.00	0.45	0.62	-0.04	0.20	0.75	0.47	0.76
Isovaleric acid	0.29	0.25	0.96	0.45	1.00	0.84	0.44	0.53	0.36	0.99	0.39
Valeric acid	0.52	0.62	0.84	0.62	0.84	1.00	0.15	0.32	0.60	0.84	0.63

Continued Table 3：The R of Spearman’s correlation analysis among obesity-related biochemical indexes, gut microbiota, SCFA, endocrine hormone, and differential metabolites.

R	Acetic acid	Propanoic acid	Isobutyric acid	Butanoic acid	Isovaleric acid	Valeric acid	Isohexanoic acid	Hexanoic acid	Straight-chainfattyacids	Branched-chainfattyacids	Total SCFA
Isohexanoic acid	-0.12	-0.44	0.31	-0.04	0.44	0.15	1.00	0.80	-0.13	0.42	-0.11
Hexanoic acid	0.15	-0.19	0.47	0.20	0.53	0.32	0.80	1.00	0.16	0.53	0.18
Straight-chainfattyacids	0.98	0.81	0.40	0.75	0.36	0.60	-0.13	0.16	1.00	0.37	1.00
Branched-chainfattyacids	0.29	0.29	0.99	0.47	0.99	0.84	0.42	0.53	0.37	1.00	0.40
TotalSCFA	0.98	0.81	0.43	0.76	0.39	0.63	-0.11	0.18	1.00	0.40	1.00
g__Blautia	-0.62	-0.34	-0.47	-0.54	-0.48	-0.59	-0.36	-0.49	-0.64	-0.49	-0.65
g__Romboutsia	-0.46	-0.04	-0.15	-0.34	-0.12	0.07	-0.08	-0.33	-0.43	-0.14	-0.43
g__Lachnoclostridium	-0.49	-0.11	-0.26	-0.42	-0.23	-0.09	-0.37	-0.53	-0.48	-0.26	-0.49
g__Staphylococcus	-0.53	-0.18	-0.28	-0.46	-0.24	-0.14	-0.19	-0.42	-0.52	-0.27	-0.53
g__unclassified_f__Oscillospiraceae	0.53	-0.01	0.15	0.15	0.19	0.18	0.50	0.62	0.45	0.20	0.45
g__Ruminococcus	0.53	0.29	0.25	0.22	0.24	0.24	0.33	0.38	0.50	0.26	0.50
g__Lactobacillus	0.34	-0.12	0.25	0.28	0.35	0.19	0.64	0.78	0.33	0.33	0.34
g__norank_f__Muribaculaceae	0.75	0.67	0.21	0.54	0.07	0.27	-0.32	0.03	0.75	0.13	0.75
g__Lachnospiraceae_NK4A136_group	0.75	0.63	0.28	0.80	0.27	0.46	-0.33	-0.09	0.80	0.25	0.80
g__norank_o__Clostridia_UCG-014	0.19	0.04	0.09	0.21	0.07	0.08	0.31	0.28	0.20	0.10	0.20
Ehhadh	0.39	0.50	-0.10	0.36	-0.20	0.04	-0.45	-0.39	0.41	-0.17	0.40
Acox1	0.29	0.34	0.04	0.44	-0.10	-0.07	-0.49	-0.32	0.33	-0.05	0.33
Acsl1	0.46	0.52	0.09	0.54	-0.06	0.10	-0.60	-0.36	0.50	-0.01	0.49
Fabp4	-0.24	-0.45	-0.19	-0.09	-0.04	-0.13	0.45	0.12	-0.25	-0.10	-0.25
Lpl	0.35	0.35	0.07	0.51	-0.07	0.04	-0.29	-0.08	0.40	-0.01	0.39
Pparg	0.31	0.35	0.10	0.23	-0.06	-0.04	-0.61	-0.39	0.31	-0.01	0.30
Ndufaf1	0.53	0.46	-0.10	0.43	-0.22	-0.06	-0.58	-0.37	0.52	-0.18	0.51
Ndufs1	0.60	0.56	0.13	0.54	-0.01	0.11	-0.33	-0.11	0.62	0.05	0.62

Continued Table 3：The R of Spearman's correlation analysis among obesity-related biochemical indexes, gut microbiota, SCFA, endocrine hormone, and differential metabolites.

R	Acetic acid	Propanoic acid	Isobutyric acid	Butanoic acid	Isovaleric acid	Valeric acid	Isohexanoic acid	Hexanoic acid	Straight-chainfattyacids	Branched-chainfattyacids	Total SCFA
Klhl13	0.35	0.17	0.14	0.30	0.12	0.05	0.35	0.39	0.35	0.16	0.36
Prdm16	0.23	0.11	0.28	0.18	0.20	0.11	0.18	0.36	0.23	0.25	0.24
Elovl2	0.22	-0.01	0.38	0.22	0.38	0.24	0.50	0.73	0.23	0.41	0.24
Plpp3	0.22	0.12	0.33	0.16	0.31	0.12	0.36	0.43	0.22	0.34	0.23
Lpcat3	0.26	0.71	0.03	0.28	-0.11	0.21	-0.71	-0.69	0.31	-0.08	0.31
Pld4	0.60	0.75	0.11	0.43	0.09	0.44	-0.52	-0.30	0.62	0.07	0.62
TG(18:4/18:2/22:6)	0.28	-0.11	0.18	0.27	0.21	0.06	0.59	0.78	0.27	0.23	0.28
TG(20:5/18:2/22:6)	0.47	0.12	0.34	0.37	0.39	0.37	0.62	0.77	0.47	0.39	0.48
DG(18:3/22:6)	0.38	-0.02	0.33	0.27	0.38	0.28	0.71	0.85	0.37	0.39	0.38
DG(20:4/22:6)	0.49	0.15	0.40	0.41	0.41	0.40	0.61	0.76	0.49	0.43	0.50
OAHFA(22:6/22:5)	0.27	-0.01	0.25	0.21	0.25	0.18	0.65	0.83	0.27	0.29	0.28
FA(22:6)	0.13	-0.20	0.23	0.08	0.29	0.14	0.78	0.88	0.12	0.30	0.13
PE(18:2e/22:6)	0.58	0.27	0.37	0.42	0.36	0.38	0.48	0.65	0.58	0.39	0.59
PE(18:1e/22:6)	0.64	0.41	0.27	0.50	0.22	0.31	0.29	0.46	0.65	0.26	0.65
PE(16:1e/22:6)	0.51	0.15	0.34	0.31	0.36	0.32	0.59	0.74	0.49	0.38	0.50
PC(18:1/22:6)	0.62	0.48	0.15	0.45	0.05	0.24	0.14	0.29	0.63	0.11	0.62
PC(16:1/22:6)	0.57	0.19	0.45	0.43	0.50	0.52	0.50	0.65	0.57	0.50	0.58

Continued Table 3：The R of Spearman's correlation analysis among obesity-related biochemical indexes, gut microbiota, SCFA, endocrine hormone, and differential metabolites.

R	g__Blautia	g__Romboutsia	g__Lachnoclostridium	g__Staphylococcus	g__unclassified_f__Oscillospiraceae	g__Ruminococcus	g__Lactobacillus	g__norank_f__Muribaculaceae	g__Lachnospiraceae_NK4A136_group	g__norank_o__Clostridia_UCG-014
Weight	0.46	0.37	0.63	0.49	-0.63	-0.57	-0.58	-0.08	0.05	-0.58
ALT	0.16	0.29	0.48	0.34	-0.13	-0.19	-0.46	-0.08	0.18	-0.24
AST	0.35	0.24	0.41	0.26	-0.47	-0.28	-0.45	-0.19	0.13	-0.45
CHO	0.28	-0.01	0.14	0.07	-0.44	-0.36	-0.49	0.23	0.27	-0.22
TG	0.67	0.35	0.57	0.48	-0.64	-0.61	-0.45	-0.37	-0.13	-0.47
HDL	-0.65	-0.50	-0.60	-0.55	0.60	0.46	0.69	0.41	0.47	0.41
LDL	0.55	0.13	0.42	0.41	-0.32	-0.37	-0.46	-0.07	0.30	-0.36
GLP-1	-0.33	0.19	0.11	0.17	0.05	0.09	-0.11	0.16	0.15	0.09
PYY	-0.47	-0.52	-0.58	-0.59	0.26	0.51	0.15	0.67	0.35	0.31
CCK	-0.29	-0.19	-0.40	-0.41	0.06	0.40	0.23	-0.02	-0.12	0.56
INS	-0.31	-0.31	-0.30	-0.26	0.13	0.09	0.31	0.58	0.13	-0.14
GAS	0.03	0.11	0.02	0.05	0.21	-0.11	-0.07	0.21	0.03	0.08
MTL	-0.19	-0.05	-0.09	-0.02	0.65	0.18	0.26	0.03	-0.12	-0.02
Acetic acid	-0.62	-0.46	-0.49	-0.53	0.53	0.53	0.34	0.75	0.75	0.19
Propanoic acid	-0.34	-0.04	-0.11	-0.18	-0.01	0.29	-0.12	0.67	0.63	0.04
Isobutyric acid	-0.47	-0.15	-0.26	-0.28	0.15	0.25	0.25	0.21	0.28	0.09
Butanoic acid	-0.54	-0.34	-0.42	-0.46	0.15	0.22	0.28	0.54	0.80	0.21
Isovaleric acid	-0.48	-0.12	-0.23	-0.24	0.19	0.24	0.35	0.07	0.27	0.07
Valeric acid	-0.59	0.07	-0.09	-0.14	0.18	0.24	0.19	0.27	0.46	0.08
Isohexanoic acid	-0.36	-0.08	-0.37	-0.19	0.50	0.33	0.64	-0.32	-0.33	0.31
Hexanoic acid	-0.49	-0.33	-0.53	-0.42	0.62	0.38	0.78	0.03	-0.09	0.28
Straight-chainfattyacids	-0.64	-0.43	-0.48	-0.52	0.45	0.50	0.33	0.75	0.80	0.20
Branched-chainfattyacids	-0.49	-0.14	-0.26	-0.27	0.20	0.26	0.33	0.13	0.25	0.10
TotalSCFA	-0.65	-0.43	-0.49	-0.53	0.45	0.50	0.34	0.75	0.80	0.20

Continued Table 3：The R of Spearman's correlation analysis among obesity-related biochemical indexes, gut microbiota, SCFA, endocrine hormone, and differential metabolites.

R	g__Blautia	g__Romboutsia	g__Lachnoclostridium	g__Staphylococcus	g__unclassified_f__Oscillospiraceae	g__Ruminococcus	g__Lactobacillus	g__norank_f__Muribaculaceae	g__Lachnospiraceae_NK4A136_group	g__norank_o__Clostridia_UCG-014
g__Blautia	1.00	0.36	0.55	0.53	-0.56	-0.63	-0.67	-0.53	-0.36	-0.46
g__Romboutsia	0.36	1.00	0.86	0.87	-0.45	-0.57	-0.51	-0.55	-0.40	-0.25
g__Lachnoclostridium	0.55	0.86	1.00	0.94	-0.56	-0.64	-0.62	-0.51	-0.28	-0.49
g__Staphylococcus	0.53	0.87	0.94	1.00	-0.49	-0.57	-0.53	-0.51	-0.34	-0.37
g__unclassified_f__Oscillospiraceae	-0.56	-0.45	-0.56	-0.49	1.00	0.51	0.55	0.29	0.15	0.37
g__Ruminococcus	-0.63	-0.57	-0.64	-0.57	0.51	1.00	0.58	0.50	0.17	0.40
g__Lactobacillus	-0.67	-0.51	-0.62	-0.53	0.55	0.58	1.00	0.17	0.16	0.22
g__norank_f__Muribaculaceae	-0.53	-0.55	-0.51	-0.51	0.29	0.50	0.17	1.00	0.57	0.32
g__Lachnospiraceae_NK4A136_group	-0.36	-0.40	-0.28	-0.34	0.15	0.17	0.16	0.57	1.00	0.08
g__norank_o__Clostridia_UCG-014	-0.46	-0.25	-0.49	-0.37	0.37	0.40	0.22	0.32	0.08	1.00
Ehhadh	-0.16	-0.32	-0.25	-0.30	0.04	0.28	-0.27	0.58	0.44	0.42
Acox1	-0.02	-0.41	-0.25	-0.43	-0.11	0.03	-0.10	0.35	0.50	0.12
Acsl1	-0.18	-0.38	-0.20	-0.36	-0.08	0.07	-0.13	0.61	0.66	0.17
Fabp4	-0.04	0.19	0.07	0.10	0.18	-0.21	0.05	-0.34	-0.25	-0.08
Lpl	-0.34	-0.42	-0.42	-0.49	0.03	0.17	0.09	0.58	0.48	0.53
Pparg	0.07	-0.36	-0.14	-0.35	-0.11	-0.07	-0.25	0.35	0.41	-0.09
Ndufaf1	-0.16	-0.43	-0.26	-0.43	0.08	0.12	-0.06	0.55	0.61	0.15
Ndufs1	-0.32	-0.47	-0.45	-0.54	0.13	0.29	0.13	0.62	0.61	0.38
Klhl13	-0.53	-0.51	-0.64	-0.57	0.31	0.75	0.64	0.38	0.16	0.57
Prdm16	-0.22	-0.44	-0.43	-0.35	0.35	0.47	0.40	0.21	0.20	0.36
Elovl2	-0.22	-0.33	-0.49	-0.40	0.53	0.31	0.37	0.18	0.01	0.47
Plpp3	-0.38	-0.47	-0.50	-0.42	0.20	0.78	0.57	0.28	0.01	0.38

Continued Table 3：The R of Spearman's correlation analysis among obesity-related biochemical indexes, gut microbiota, SCFA, endocrine hormone, and differential metabolites.

R	g__Blautia	g__Romboutsia	g__Lachnoclostridium	g__Staphylococcus	g__unclassified_f__Oscillospiraceae	g__Ruminococcus	g__Lactobacillus	g__norank_f__Muribaculaceae	g__Lachnospiraceae_NK4A136_group	g__norank_o__Clostridia_UCG-014
Lpcat3	0.08	0.26	0.35	0.20	-0.50	0.00	-0.51	0.32	0.36	-0.12
Pld4	-0.23	-0.02	0.08	0.05	-0.12	0.21	-0.12	0.57	0.68	0.04
TG(18:4/18:2/22:6)	-0.49	-0.62	-0.71	-0.62	0.65	0.66	0.74	0.26	0.07	0.54
TG(20:5/18:2/22:6)	-0.72	-0.50	-0.71	-0.61	0.73	0.73	0.75	0.35	0.18	0.58
DG(18:3/22:6)	-0.64	-0.50	-0.69	-0.54	0.74	0.71	0.83	0.25	0.13	0.54
DG(20:4/22:6)	-0.72	-0.49	-0.72	-0.58	0.71	0.72	0.74	0.34	0.21	0.55
OAHFA(22:6/22:5)	-0.52	-0.40	-0.61	-0.48	0.63	0.64	0.70	0.25	-0.04	0.54
FA(22:6)	-0.46	-0.33	-0.56	-0.41	0.60	0.56	0.74	0.05	-0.16	0.50
PE(18:2e/22:6)	-0.85	-0.53	-0.73	-0.64	0.64	0.83	0.76	0.55	0.25	0.59
PE(18:1e/22:6)	-0.77	-0.57	-0.73	-0.64	0.56	0.82	0.64	0.66	0.38	0.63
PE(16:1e/22:6)	-0.79	-0.52	-0.73	-0.62	0.70	0.81	0.81	0.41	0.14	0.56
PC(18:1/22:6)	-0.73	-0.44	-0.63	-0.56	0.46	0.80	0.52	0.67	0.30	0.53
PC(16:1/22:6)	-0.91	-0.47	-0.63	-0.57	0.67	0.74	0.76	0.41	0.30	0.55

Continued Table 3：The R of Spearman's correlation analysis among obesity-related biochemical indexes, gut microbiota, SCFA, endocrine hormone, and differential metabolites.

R	Ehhadh	Acox1	Acsl1	Fabp4	Lpl	Pparg	Ndufaf1	Ndufs1	Klhl13	Prdm16	Elovl2	Plpp3	Lpcat3	Pld4
Weight	-0.19	0.04	0.15	-0.17	-0.16	0.21	0.07	-0.21	-0.72	-0.39	-0.44	-0.59	0.48	0.23
ALT	0.48	0.08	0.17	-0.01	-0.10	0.27	0.20	-0.01	-0.34	-0.35	-0.54	-0.30	0.35	0.48
AST	0.38	0.14	0.10	-0.09	-0.17	0.29	0.09	-0.03	-0.28	-0.33	-0.52	-0.13	0.36	0.36
CHO	0.28	0.44	0.58	-0.25	0.45	0.55	0.38	0.31	-0.33	-0.20	-0.51	-0.48	0.44	0.32
TG	-0.29	0.22	0.09	-0.32	-0.09	0.34	0.12	-0.03	-0.45	-0.16	-0.45	-0.38	0.30	-0.03
HDL	0.15	0.18	0.17	-0.28	0.31	0.14	0.33	0.52	0.58	0.54	0.26	0.41	-0.25	0.21
LDL	0.10	0.20	0.23	-0.31	-0.07	0.41	0.22	0.13	-0.39	-0.10	-0.41	-0.32	0.31	0.40
GLP-1	0.24	-0.05	0.13	-0.17	0.11	0.07	-0.09	0.06	-0.02	0.16	-0.31	-0.02	0.17	0.30
PYY	0.52	0.23	0.39	-0.33	0.46	0.40	0.31	0.50	0.31	0.06	0.08	0.24	0.14	0.51

Continued Table 3：The R of Spearman's correlation analysis among obesity-related biochemical indexes, gut microbiota, SCFA, endocrine hormone, and differential metabolites.

R	Ehhadh	Acox1	Acsl1	Fabp4	Lpl	Pparg	Ndufaf1	Ndufs1	Klhl13	Prdm16	Elovl2	Plpp3	Lpcat3	Pld4
CCK	0.11	0.17	-0.03	-0.08	0.18	-0.03	-0.02	0.19	0.56	0.34	0.49	0.64	-0.02	-0.26
INS	-0.16	-0.01	0.15	-0.12	0.18	0.11	0.05	0.15	0.03	-0.05	0.25	0.09	-0.09	0.11
GAS	-0.20	-0.28	-0.08	0.28	-0.05	-0.31	-0.12	-0.15	-0.27	-0.30	0.58	-0.32	-0.04	0.07
MTL	-0.28	-0.48	-0.35	0.53	-0.34	-0.44	-0.27	-0.38	-0.18	-0.24	0.42	-0.18	-0.42	-0.15
Acetic acid	0.39	0.29	0.46	-0.24	0.35	0.31	0.53	0.60	0.35	0.23	0.22	0.22	0.26	0.60
Propanoic acid	0.50	0.34	0.52	-0.45	0.35	0.35	0.46	0.56	0.17	0.11	-0.01	0.12	0.71	0.75
Isobutyric acid	-0.10	0.04	0.09	-0.19	0.07	0.10	-0.10	0.13	0.14	0.28	0.38	0.33	0.03	0.11
Butanoic acid	0.36	0.44	0.54	-0.09	0.51	0.23	0.43	0.54	0.30	0.18	0.22	0.16	0.28	0.43
Isovaleric acid	-0.20	-0.10	-0.06	-0.04	-0.07	-0.06	-0.22	-0.01	0.12	0.20	0.38	0.31	-0.11	0.09
Valeric acid	0.04	-0.07	0.10	-0.13	0.04	-0.04	-0.06	0.11	0.05	0.11	0.24	0.12	0.21	0.44
Isohexanoic acid	-0.45	-0.49	-0.60	0.45	-0.29	-0.61	-0.58	-0.33	0.35	0.18	0.50	0.36	-0.71	-0.52
Hexanoic acid	-0.39	-0.32	-0.36	0.12	-0.08	-0.39	-0.37	-0.11	0.39	0.36	0.73	0.43	-0.69	-0.30
Straight-chainfattyacids	0.41	0.33	0.50	-0.25	0.40	0.31	0.52	0.62	0.35	0.23	0.23	0.22	0.31	0.62
Branched-chainfattyacids	-0.17	-0.05	-0.01	-0.10	-0.01	-0.01	-0.18	0.05	0.16	0.25	0.41	0.34	-0.08	0.07
TotalSCFA	0.40	0.33	0.49	-0.25	0.39	0.30	0.51	0.62	0.36	0.24	0.24	0.23	0.31	0.62
g__Blautia	-0.16	-0.02	-0.18	-0.04	-0.34	0.07	-0.16	-0.32	-0.53	-0.22	-0.22	-0.38	0.08	-0.23
g__Romboutsia	-0.32	-0.41	-0.38	0.19	-0.42	-0.36	-0.43	-0.47	-0.51	-0.44	-0.33	-0.47	0.26	-0.02
g__Lachnoclostridium	-0.25	-0.25	-0.20	0.07	-0.42	-0.14	-0.26	-0.45	-0.64	-0.43	-0.49	-0.50	0.35	0.08
g__Staphylococcus	-0.30	-0.43	-0.36	0.10	-0.49	-0.35	-0.43	-0.54	-0.57	-0.35	-0.40	-0.42	0.20	0.05
g__unclassified_f__Oscillospiraceae	0.04	-0.11	-0.08	0.18	0.03	-0.11	0.08	0.13	0.31	0.35	0.53	0.20	-0.50	-0.12
g__Ruminococcus	0.28	0.03	0.07	-0.21	0.17	-0.07	0.12	0.29	0.75	0.47	0.31	0.78	0.00	0.21
g__Lactobacillus	-0.27	-0.10	-0.13	0.05	0.09	-0.25	-0.06	0.13	0.64	0.40	0.37	0.57	-0.51	-0.12
g__norank_f__Muribaculaceae	0.58	0.35	0.61	-0.34	0.58	0.35	0.55	0.62	0.38	0.21	0.18	0.28	0.32	0.57
g__Lachnospiraceae_NK4A136_group	0.44	0.50	0.66	-0.25	0.48	0.41	0.61	0.61	0.16	0.20	0.01	0.01	0.36	0.68
g__norank_o__Clostridia_UCG-014	0.42	0.12	0.17	-0.08	0.53	-0.09	0.15	0.38	0.57	0.36	0.47	0.38	-0.12	0.04
Ehhadh	1.00	0.51	0.63	-0.27	0.59	0.46	0.60	0.60	0.31	0.10	-0.17	0.17	0.43	0.54
Acox1	0.51	1.00	0.89	-0.39	0.78	0.88	0.91	0.86	0.33	0.24	-0.17	0.14	0.50	0.16
Acsl1	0.63	0.89	1.00	-0.35	0.86	0.82	0.91	0.86	0.25	0.10	-0.20	0.02	0.60	0.47

Continued Table 3：The R of Spearman's correlation analysis among obesity-related biochemical indexes, gut microbiota, SCFA, endocrine hormone, and differential metabolites.

R	Ehhadh	Acox1	Acsl1	Fabp4	Lpl	Pparg	Ndufaf1	Ndufs1	Klhl13	Prdm16	Elovl2	Plpp3	Lpcat3	Pld4
Fabp4	-0.27	-0.39	-0.35	1.00	-0.27	-0.44	-0.31	-0.45	-0.25	-0.65	-0.04	-0.42	-0.38	-0.43
Lpl	0.59	0.78	0.86	-0.27	1.00	0.61	0.76	0.87	0.51	0.17	-0.01	0.17	0.34	0.30
Pparg	0.46	0.88	0.82	-0.44	0.61	1.00	0.82	0.74	0.07	0.10	-0.28	-0.02	0.46	0.22
Ndufaf1	0.60	0.91	0.91	-0.31	0.76	0.82	1.00	0.88	0.31	0.16	-0.18	0.05	0.52	0.33
Ndufs1	0.60	0.86	0.86	-0.45	0.87	0.74	0.88	1.00	0.58	0.33	0.00	0.30	0.44	0.39
Klhl13	0.31	0.33	0.25	-0.25	0.51	0.07	0.31	0.58	1.00	0.54	0.25	0.84	-0.02	0.06
Prdm16	0.10	0.24	0.10	-0.65	0.17	0.10	0.16	0.33	0.54	1.00	0.40	0.64	-0.13	-0.03
Elovl2	-0.17	-0.17	-0.20	-0.04	-0.01	-0.28	-0.18	0.00	0.25	0.40	1.00	0.35	-0.41	-0.20
Plpp3	0.17	0.14	0.02	-0.42	0.17	-0.02	0.05	0.30	0.84	0.64	0.35	1.00	-0.05	0.03
Lpcat3	0.43	0.50	0.60	-0.38	0.34	0.46	0.52	0.44	-0.02	-0.13	-0.41	-0.05	1.00	0.57
Pld4	0.54	0.16	0.47	-0.43	0.30	0.22	0.33	0.39	0.06	-0.03	-0.20	0.03	0.57	1.00
TG(18:4/18:2/22:6)	0.10	0.02	-0.04	-0.04	0.23	-0.18	0.01	0.21	0.71	0.51	0.67	0.69	-0.50	-0.13
TG(20:5/18:2/22:6)	0.20	-0.10	-0.05	-0.01	0.23	-0.22	-0.03	0.24	0.69	0.42	0.56	0.60	-0.42	0.10
DG(18:3/22:6)	-0.02	-0.18	-0.17	-0.04	0.12	-0.32	-0.14	0.15	0.68	0.56	0.63	0.62	-0.53	-0.03
DG(20:4/22:6)	0.07	-0.10	-0.08	-0.14	0.18	-0.22	-0.09	0.22	0.62	0.57	0.57	0.57	-0.39	0.06
OAHFA(22:6/22:5)	0.03	-0.14	-0.13	-0.05	0.20	-0.27	-0.14	0.17	0.67	0.42	0.66	0.63	-0.45	-0.05
FA(22:6)	-0.15	-0.29	-0.33	0.07	0.04	-0.41	-0.32	-0.01	0.60	0.35	0.65	0.58	-0.61	-0.18
PE(18:2e/22:6)	0.22	0.05	0.15	-0.11	0.41	-0.08	0.14	0.41	0.79	0.43	0.42	0.65	-0.19	0.19
PE(18:1e/22:6)	0.45	0.23	0.31	-0.26	0.53	0.05	0.32	0.59	0.86	0.53	0.34	0.69	-0.02	0.31
PE(16:1e/22:6)	0.11	-0.03	0.00	-0.08	0.29	-0.14	0.04	0.33	0.79	0.46	0.48	0.68	-0.34	0.06
PC(18:1/22:6)	0.35	0.25	0.33	-0.25	0.50	0.04	0.36	0.55	0.76	0.46	0.23	0.56	0.18	0.26
PC(16:1/22:6)	0.13	-0.06	0.05	-0.06	0.26	-0.13	0.03	0.25	0.60	0.38	0.41	0.53	-0.27	0.21

Continued Table 3：The R of Spearman's correlation analysis among obesity-related biochemical indexes, gut microbiota, SCFA, endocrine hormone, and differential metabolites.

R	TG(18:4/18:2/22:6)	TG(20:5/18:2/22:6)	DG(18:3/22:6)	DG(20:4/22:6)	OAHFA(22:6/22:5)	FA(22:6)	PE(18:2e/22:6)	PE(18:1e/22:6)	PE(16:1e/22:6)	PC(18:1/22:6)	PC(16:1/22:6)
Weight	-0.79	-0.84	-0.80	-0.74	-0.79	-0.79	-0.71	-0.70	-0.79	-0.47	-0.60
ALT	-0.37	-0.22	-0.42	-0.36	-0.39	-0.42	-0.31	-0.23	-0.34	-0.31	-0.16
AST	-0.38	-0.32	-0.51	-0.45	-0.44	-0.45	-0.44	-0.33	-0.44	-0.46	-0.36
CHO	-0.53	-0.49	-0.52	-0.37	-0.47	-0.56	-0.32	-0.23	-0.45	-0.09	-0.39
TG	-0.63	-0.80	-0.70	-0.68	-0.65	-0.60	-0.72	-0.67	-0.70	-0.52	-0.68
HDL	0.51	0.64	0.65	0.64	0.45	0.42	0.66	0.69	0.69	0.54	0.69
LDL	-0.52	-0.54	-0.49	-0.47	-0.53	-0.53	-0.53	-0.40	-0.53	-0.43	-0.47
GLP-1	-0.20	0.11	0.08	0.21	-0.02	-0.07	0.20	0.21	0.12	0.18	0.24
PYY	0.26	0.45	0.28	0.47	0.33	0.23	0.56	0.56	0.48	0.51	0.53
CCK	0.46	0.40	0.35	0.36	0.39	0.40	0.37	0.36	0.40	0.26	0.36
INS	0.17	0.14	0.14	0.19	0.24	0.17	0.29	0.21	0.24	0.23	0.20
GAS	0.10	0.05	0.07	0.01	0.20	0.16	0.01	-0.08	-0.01	0.00	-0.04
MTL	0.35	0.32	0.34	0.29	0.38	0.41	0.22	0.05	0.27	0.08	0.27
Acetic acid	0.28	0.47	0.38	0.49	0.27	0.13	0.58	0.64	0.51	0.62	0.57
Propanoic acid	-0.11	0.12	-0.02	0.15	-0.01	-0.20	0.27	0.41	0.15	0.48	0.19
Isobutyric acid	0.18	0.34	0.33	0.40	0.25	0.23	0.37	0.27	0.34	0.15	0.45
Butanoic acid	0.27	0.37	0.27	0.41	0.21	0.08	0.42	0.50	0.31	0.45	0.43
Isovaleric acid	0.21	0.39	0.38	0.41	0.25	0.29	0.36	0.22	0.36	0.05	0.50
Valeric acid	0.06	0.37	0.28	0.40	0.18	0.14	0.38	0.31	0.32	0.24	0.52
Isohexanoic acid	0.59	0.62	0.71	0.61	0.65	0.78	0.48	0.29	0.59	0.14	0.50
Hexanoic acid	0.78	0.77	0.85	0.76	0.83	0.88	0.65	0.46	0.74	0.29	0.65
Straight-chainfattyacids	0.27	0.47	0.37	0.49	0.27	0.12	0.58	0.65	0.49	0.63	0.57
Branched-chainfattyacids	0.23	0.39	0.39	0.43	0.29	0.30	0.39	0.26	0.38	0.11	0.50
TotalSCFA	0.28	0.48	0.38	0.50	0.28	0.13	0.59	0.65	0.50	0.62	0.58
g__Blautia	-0.49	-0.72	-0.64	-0.72	-0.52	-0.46	-0.85	-0.77	-0.79	-0.73	-0.91
g__Romboutsia	-0.62	-0.50	-0.50	-0.49	-0.40	-0.33	-0.53	-0.57	-0.52	-0.44	-0.47
g__Lachnoclostridium	-0.71	-0.71	-0.69	-0.72	-0.61	-0.56	-0.73	-0.73	-0.73	-0.63	-0.63

Continued Table 3：The R of Spearman’s correlation analysis among obesity-related biochemical indexes, gut microbiota, SCFA, endocrine hormone, and differential metabolites.

R	TG(18:4/18:2/22:6)	TG(20:5/18:2/22:6)	DG(18:3/22:6)	DG(20:4/22:6)	OAHFA(22:6/22:5)	FA(22:6)	PE(18:2e/22:6)	PE(18:1e/22:6)	PE(16:1e/22:6)	PC(18:1/22:6)	PC(16:1/22:6)
g__Staphylococcus	-0.62	-0.61	-0.54	-0.58	-0.48	-0.41	-0.64	-0.64	-0.62	-0.56	-0.57
g__unclassified_f__Oscillospiraceae	0.65	0.73	0.74	0.71	0.63	0.60	0.64	0.56	0.70	0.46	0.67
g__Ruminococcus	0.66	0.73	0.71	0.72	0.64	0.56	0.83	0.82	0.81	0.80	0.74
g__Lactobacillus	0.74	0.75	0.83	0.74	0.70	0.74	0.76	0.64	0.81	0.52	0.76
g__norank_f__Muribaculaceae	0.26	0.35	0.25	0.34	0.25	0.05	0.55	0.66	0.41	0.67	0.41
g__Lachnospiraceae_NK4A136_group	0.07	0.18	0.13	0.21	-0.04	-0.16	0.25	0.38	0.14	0.30	0.30
g__norank_o__Clostridia_UCG-014	0.54	0.58	0.54	0.55	0.54	0.50	0.59	0.63	0.56	0.53	0.55
Ehhadh	0.10	0.20	-0.02	0.07	0.03	-0.15	0.22	0.45	0.11	0.35	0.13
Acox1	0.02	-0.10	-0.18	-0.10	-0.14	-0.29	0.05	0.23	-0.03	0.25	-0.06
Acsl1	-0.04	-0.05	-0.17	-0.08	-0.13	-0.33	0.15	0.31	0.00	0.33	0.05
Fabp4	-0.04	-0.01	-0.04	-0.14	-0.05	0.07	-0.11	-0.26	-0.08	-0.25	-0.06
Lpl	0.23	0.23	0.12	0.18	0.20	0.04	0.41	0.53	0.29	0.50	0.26
Pparg	-0.18	-0.22	-0.32	-0.22	-0.27	-0.41	-0.08	0.05	-0.14	0.04	-0.13
Ndufaf1	0.01	-0.03	-0.14	-0.09	-0.14	-0.32	0.14	0.32	0.04	0.36	0.03
Ndufs1	0.21	0.24	0.15	0.22	0.17	-0.01	0.41	0.59	0.33	0.55	0.25
Klhl13	0.71	0.69	0.68	0.62	0.67	0.60	0.79	0.86	0.79	0.76	0.60
Prdm16	0.51	0.42	0.56	0.57	0.42	0.35	0.43	0.53	0.46	0.46	0.38
Elovl2	0.67	0.56	0.63	0.57	0.66	0.65	0.42	0.34	0.48	0.23	0.41
Plpp3	0.69	0.60	0.62	0.57	0.63	0.58	0.65	0.69	0.68	0.56	0.53
Lpcat3	-0.50	-0.42	-0.53	-0.39	-0.45	-0.61	-0.19	-0.02	-0.34	0.18	-0.27
Pld4	-0.13	0.10	-0.03	0.06	-0.05	-0.18	0.19	0.31	0.06	0.26	0.21
TG(18:4/18:2/22:6)	1.00	0.89	0.89	0.81	0.92	0.89	0.78	0.73	0.84	0.53	0.71
TG(20:5/18:2/22:6)	0.89	1.00	0.95	0.92	0.91	0.88	0.92	0.85	0.95	0.64	0.88

Continued Table 3：The R of Spearman's correlation analysis among obesity-related biochemical indexes, gut microbiota, SCFA, endocrine hormone, and differential metabolites.

R	TG(18:4/18:2/22:6)	TG(20:5/18:2/22:6)	DG(18:3/22:6)	DG(20:4/22:6)	OAHFA(22:6/22:5)	FA(22:6)	PE(18:2e/22:6)	PE(18:1e/22:6)	PE(16:1e/22:6)	PC(18:1/22:6)	PC(16:1/22:6)
DG(18:3/22:6)	0.89	0.95	1.00	0.94	0.92	0.91	0.87	0.79	0.93	0.61	0.84
DG(20:4/22:6)	0.81	0.92	0.94	1.00	0.86	0.83	0.89	0.82	0.91	0.70	0.89
OAHFA(22:6/22:5)	0.92	0.91	0.92	0.86	1.00	0.96	0.84	0.75	0.90	0.58	0.73
FA(22:6)	0.89	0.88	0.91	0.83	0.96	1.00	0.76	0.62	0.85	0.43	0.70
PE(18:2e/22:6)	0.78	0.92	0.87	0.89	0.84	0.76	1.00	0.94	0.98	0.85	0.92
PE(18:1e/22:6)	0.73	0.85	0.79	0.82	0.75	0.62	0.94	1.00	0.90	0.90	0.81
PE(16:1e/22:6)	0.84	0.95	0.93	0.91	0.90	0.85	0.98	0.90	1.00	0.77	0.90
PC(18:1/22:6)	0.53	0.64	0.61	0.70	0.58	0.43	0.85	0.90	0.77	1.00	0.68
PC(16:1/22:6)	0.71	0.88	0.84	0.89	0.73	0.70	0.92	0.81	0.90	0.68	1.00

Supplementary Table 4：The *P* values of Spearman's correlation analysis among obesity-related biochemical indexes, gut microbiota, SCFA, endocrine hormone, and differential metabolites.Related to Figure 10A.

P	Weight	ALT	AST	CHO	TG	HDL	LDL	GLP-1	PYY	CCK	INS	GAS	MTL
Weight		0.326	0.329	0.018	0.000	0.040	0.037	0.728	0.463	0.027	0.680	0.558	0.491
ALT	0.326		0.000	0.635	0.574	0.611	0.058	0.475	0.660	0.209	0.089	0.301	0.871
AST	0.329	0.000		0.598	0.392	0.284	0.104	0.811	0.880	0.695	0.248	0.110	0.146
CHO	0.018	0.635	0.598		0.058	0.204	0.065	0.198	0.284	0.028	0.594	0.752	0.126
TG	0.000	0.574	0.392	0.058		0.216	0.002	0.303	0.260	0.115	0.749	0.559	0.134
HDL	0.040	0.611	0.284	0.204	0.216		0.718	0.896	0.120	0.515	0.514	0.441	0.946
LDL	0.037	0.058	0.104	0.065	0.002	0.718		0.972	0.939	0.025	0.598	0.546	0.398
GLP-1	0.728	0.475	0.811	0.198	0.303	0.896	0.972		0.387	0.665	0.860	0.130	0.278
PYY	0.463	0.660	0.880	0.284	0.260	0.120	0.939	0.387		0.789	0.116	0.859	0.996
CCK	0.027	0.209	0.695	0.028	0.115	0.515	0.025	0.665	0.789		0.509	0.561	0.291
INS	0.680	0.089	0.248	0.594	0.749	0.514	0.598	0.860	0.116	0.509		0.150	0.404
GAS	0.558	0.301	0.110	0.752	0.559	0.441	0.546	0.130	0.859	0.561	0.150		0.004
MTL	0.491	0.871	0.146	0.126	0.134	0.946	0.398	0.278	0.996	0.291	0.404	0.004	
Acetic acid	0.420	0.706	0.799	0.858	0.120	0.002	0.836	0.706	0.007	0.980	0.188	0.513	0.465
Propanoic acid	0.701	0.424	0.362	0.349	0.518	0.328	0.670	0.211	0.033	0.939	0.308	0.627	0.411
Isobutyric acid	0.436	0.318	0.797	0.564	0.123	0.404	0.309	0.061	0.342	0.048	0.216	0.921	0.513
Butanoic acid	0.466	0.714	0.875	0.671	0.099	0.141	0.426	0.508	0.196	0.385	0.246	0.638	0.708
Isovaleric acid	0.328	0.448	0.915	0.216	0.084	0.296	0.300	0.131	0.563	0.051	0.424	0.902	0.765
Valeric acid	0.704	0.826	0.739	0.592	0.066	0.290	0.336	0.034	0.270	0.297	0.493	0.658	0.897
Isohexanoic acid	0.000	0.040	0.066	0.002	0.012	0.355	0.016	0.961	0.513	0.071	0.878	0.886	0.128
Hexanoic acid	0.004	0.014	0.030	0.017	0.014	0.075	0.010	0.852	0.742	0.166	0.130	0.329	0.080
Straight-chainfattyacids	0.431	0.795	0.905	0.974	0.094	0.005	0.993	0.567	0.009	0.847	0.163	0.498	0.650
Branched-chainfattyacids	0.298	0.313	0.764	0.313	0.079	0.333	0.247	0.090	0.459	0.039	0.305	0.921	0.686
TotalSCFA	0.415	0.826	0.897	0.945	0.086	0.005	0.959	0.532	0.009	0.793	0.157	0.507	0.666
g__Blautia	0.055	0.526	0.161	0.267	0.002	0.003	0.019	0.179	0.046	0.251	0.210	0.891	0.461
g__Romboutsia	0.128	0.249	0.339	0.976	0.156	0.034	0.606	0.455	0.028	0.443	0.207	0.656	0.830
g__Lachnoclostridium	0.005	0.043	0.091	0.572	0.013	0.009	0.081	0.666	0.012	0.097	0.222	0.937	0.714

Continued Table 4：The *P* values of Spearman's correlation analysis among obesity-related biochemical indexes, gut microbiota, SCFA, endocrine hormone, and differential metabolites.

P	Weight	ALT	AST	CHO	TG	HDL	LDL	GLP-1	PYY	CCK	INS	GAS	MTL
g__Staphylococcus	0.040	0.174	0.297	0.786	0.043	0.017	0.090	0.501	0.010	0.088	0.295	0.851	0.924
g__unclassified_f__Oscillospiraceae	0.005	0.613	0.050	0.066	0.004	0.009	0.197	0.851	0.295	0.827	0.619	0.402	0.004
g__Ruminococcus	0.014	0.456	0.268	0.138	0.007	0.053	0.130	0.711	0.030	0.103	0.733	0.652	0.476
g__Lactobacillus	0.011	0.052	0.060	0.039	0.059	0.001	0.057	0.654	0.553	0.362	0.215	0.770	0.299
g__norank_f__Muribaculaceae	0.765	0.742	0.449	0.366	0.133	0.094	0.781	0.522	0.002	0.937	0.011	0.406	0.899
g__Lachnospiraceae_NK4A136_group	0.837	0.483	0.603	0.273	0.621	0.049	0.231	0.555	0.157	0.623	0.606	0.894	0.629
g__norank_o__Clostridia_UCG-014	0.012	0.342	0.061	0.372	0.049	0.092	0.138	0.709	0.203	0.015	0.587	0.749	0.948
Ehhadh	0.444	0.045	0.124	0.266	0.243	0.555	0.680	0.344	0.025	0.654	0.517	0.418	0.262
Acox1	0.862	0.756	0.575	0.068	0.385	0.464	0.428	0.851	0.351	0.499	0.954	0.265	0.043
Acsl1	0.540	0.500	0.688	0.012	0.717	0.494	0.365	0.598	0.107	0.907	0.543	0.755	0.150
Fabp4	0.491	0.970	0.714	0.308	0.189	0.252	0.208	0.499	0.176	0.739	0.644	0.269	0.023
Lpl	0.538	0.680	0.494	0.063	0.736	0.216	0.798	0.656	0.057	0.474	0.484	0.858	0.171
Pparg	0.409	0.280	0.239	0.018	0.168	0.592	0.092	0.795	0.097	0.895	0.657	0.205	0.067
Ndufaf1	0.786	0.416	0.732	0.124	0.644	0.175	0.377	0.729	0.207	0.938	0.844	0.649	0.282
Ndufs1	0.412	0.971	0.893	0.215	0.915	0.028	0.609	0.823	0.035	0.442	0.562	0.541	0.120
Klhl13	0.001	0.173	0.267	0.183	0.059	0.011	0.112	0.934	0.217	0.017	0.894	0.275	0.469
Prdm16	0.106	0.156	0.181	0.425	0.532	0.022	0.693	0.513	0.824	0.167	0.859	0.230	0.342
Elovl2	0.067	0.021	0.025	0.031	0.064	0.291	0.092	0.212	0.752	0.038	0.315	0.012	0.085
Plpp3	0.010	0.228	0.614	0.044	0.118	0.087	0.202	0.927	0.335	0.004	0.715	0.199	0.466
Lpcat3	0.045	0.157	0.137	0.069	0.220	0.326	0.211	0.501	0.588	0.944	0.720	0.876	0.081
Pld4	0.349	0.045	0.142	0.199	0.917	0.407	0.098	0.231	0.031	0.292	0.678	0.780	0.566
TG(18:4/18:2/22:6)	0.000	0.133	0.117	0.025	0.005	0.033	0.028	0.418	0.291	0.055	0.496	0.691	0.155
TG(20:5/18:2/22:6)	0.000	0.382	0.199	0.040	0.000	0.004	0.022	0.663	0.062	0.099	0.593	0.835	0.194
DG(18:3/22:6)	0.000	0.085	0.031	0.028	0.001	0.004	0.039	0.762	0.252	0.150	0.571	0.792	0.162
DG(20:4/22:6)	0.001	0.140	0.061	0.131	0.002	0.004	0.049	0.406	0.051	0.146	0.462	0.961	0.241
OAHFA(22:6/22:5)	0.000	0.106	0.067	0.051	0.004	0.061	0.023	0.942	0.175	0.115	0.340	0.435	0.116
FA(22:6)	0.000	0.080	0.063	0.016	0.009	0.080	0.025	0.775	0.352	0.102	0.506	0.536	0.095

Continued Table 4：The *P* values of Spearman's correlation analysis among obesity-related biochemical indexes, gut microbiota, SCFA, endocrine hormone, and differential metabolites.

P	Weight	ALT	AST	CHO	TG	HDL	LDL	GLP-1	PYY	CCK	INS	GAS	MTL
PE(18:2e/22:6)	0.001	0.206	0.067	0.192	0.001	0.003	0.025	0.430	0.016	0.129	0.251	0.976	0.380
PE(18:1e/22:6)	0.001	0.358	0.179	0.367	0.002	0.001	0.098	0.397	0.015	0.139	0.411	0.761	0.853
PE(16:1e/22:6)	0.000	0.174	0.065	0.061	0.001	0.002	0.023	0.628	0.042	0.102	0.335	0.964	0.280
PC(18:1/22:6)	0.048	0.204	0.056	0.718	0.028	0.020	0.073	0.474	0.032	0.290	0.359	0.994	0.743
PC(16:1/22:6)	0.008	0.519	0.146	0.107	0.002	0.001	0.047	0.329	0.025	0.140	0.437	0.861	0.275

Continued Table 4：The *P* values of Spearman's correlation analysis among obesity-related biochemical indexes, gut microbiota, SCFA, endocrine hormone, and differential metabolites.

P	Acetic acid	Propanoic acid	Isobuty ric acid	Butanoic acid	Isovaleri c acid	Valeric acid	Isohexanoic acid	Hexanoic acid	Straight-chain fatty acids	Branched-cha in fatty acids	Total SCFA
Weight	0.420	0.701	0.436	0.466	0.328	0.704	0.000	0.004	0.431	0.298	0.415
ALT	0.706	0.424	0.318	0.714	0.448	0.826	0.040	0.014	0.795	0.313	0.826
AST	0.799	0.362	0.797	0.875	0.915	0.739	0.066	0.030	0.905	0.764	0.897
CHO	0.858	0.349	0.564	0.671	0.216	0.592	0.002	0.017	0.974	0.313	0.945
TG	0.120	0.518	0.123	0.099	0.084	0.066	0.012	0.014	0.094	0.079	0.086
HDL	0.002	0.328	0.404	0.141	0.296	0.290	0.355	0.075	0.005	0.333	0.005
LDL	0.836	0.670	0.309	0.426	0.300	0.336	0.016	0.010	0.993	0.247	0.959
GLP-1	0.706	0.211	0.061	0.508	0.131	0.034	0.961	0.852	0.567	0.090	0.532
PYY	0.007	0.033	0.342	0.196	0.563	0.270	0.513	0.742	0.009	0.459	0.009
CCK	0.980	0.939	0.048	0.385	0.051	0.297	0.071	0.166	0.847	0.039	0.793
INS	0.188	0.308	0.216	0.246	0.424	0.493	0.878	0.130	0.163	0.305	0.157
GAS	0.513	0.627	0.921	0.638	0.902	0.658	0.886	0.329	0.498	0.921	0.507
MTL	0.465	0.411	0.513	0.708	0.765	0.897	0.128	0.080	0.650	0.686	0.666
Acetic acid		0.000	0.200	0.005	0.246	0.028	0.622	0.561	0.000	0.239	0.000
Propanoic acid	0.000		0.127	0.005	0.326	0.006	0.069	0.445	0.000	0.247	0.000
Isobutyric acid	0.200	0.127		0.036	0.000	0.000	0.216	0.051	0.099	0.000	0.074
Butanoic acid	0.005	0.005	0.036		0.061	0.006	0.878	0.417	0.000	0.051	0.000

Continued Table 4：The *P* values of Spearman's correlation analysis among obesity-related biochemical indexes, gut microbiota, SCFA, endocrine hormone, and differential metabolites.

P	Acetic acid	Propanoic acid	Isobutyric acid	Butanoic acid	Isovaleric acid	Valeric acid	Isohexanoic acid	Hexanoic acid	Straight-chain fatty acids	Branched-chain fatty acids	Total SCFA
Isovaleric acid	0.246	0.326	0.000	0.061		0.000	0.069	0.025	0.144	0.000	0.109
Valeric acid	0.028	0.006	0.000	0.006	0.000		0.552	0.193	0.008	0.000	0.005
Isohexanoic acid	0.622	0.069	0.216	0.878	0.069	0.552		0.000	0.614	0.083	0.664
Hexanoic acid	0.561	0.445	0.051	0.417	0.025	0.193	0.000		0.523	0.022	0.478
Straight-chainfattyacids	0.000	0.000	0.099	0.000	0.144	0.008	0.614	0.523		0.131	0.000
Branched-chainfattyacids	0.239	0.247	0.000	0.051	0.000	0.000	0.083	0.022	0.131		0.099
TotalSCFA	0.000	0.000	0.074	0.000	0.109	0.005	0.664	0.478	0.000	0.099	
g__Blautia	0.006	0.162	0.051	0.019	0.041	0.010	0.147	0.037	0.004	0.039	0.003
g__Romboutsia	0.055	0.881	0.560	0.174	0.648	0.785	0.750	0.188	0.076	0.587	0.077
g__Lachnoclostridium	0.037	0.675	0.302	0.085	0.362	0.718	0.136	0.022	0.042	0.288	0.040
g__Staphylococcus	0.025	0.482	0.264	0.052	0.342	0.574	0.455	0.086	0.026	0.280	0.025
g__unclassified_f__Oscillospiraceae	0.025	0.968	0.554	0.554	0.445	0.480	0.036	0.006	0.058	0.436	0.058
g__Ruminococcus	0.022	0.250	0.316	0.391	0.341	0.339	0.188	0.118	0.035	0.293	0.034
g__Lactobacillus	0.163	0.634	0.307	0.253	0.148	0.449	0.004	0.000	0.183	0.178	0.173
g__norank_f__Muribaculaceae	0.000	0.002	0.401	0.021	0.778	0.271	0.196	0.906	0.000	0.607	0.000
g__Lachnospiraceae_NK4A136_group	0.000	0.005	0.264	0.000	0.278	0.054	0.186	0.722	0.000	0.316	0.000
g__norank_o__Clostridia_UCG-014	0.450	0.880	0.713	0.405	0.779	0.758	0.208	0.255	0.432	0.686	0.430
Ehhadh	0.106	0.036	0.690	0.146	0.436	0.871	0.063	0.106	0.088	0.509	0.099
Acox1	0.235	0.165	0.867	0.069	0.684	0.795	0.038	0.191	0.176	0.847	0.185
Acsl1	0.056	0.027	0.727	0.020	0.804	0.687	0.008	0.144	0.034	0.959	0.038
Fabp4	0.340	0.060	0.442	0.708	0.870	0.602	0.060	0.643	0.317	0.694	0.316
Lpl	0.158	0.156	0.796	0.029	0.770	0.889	0.244	0.763	0.103	0.965	0.109
Pparg	0.214	0.150	0.699	0.350	0.826	0.890	0.007	0.105	0.214	0.984	0.221

Continued Table 4：The *P* values of Spearman's correlation analysis among obesity-related biochemical indexes, gut microbiota, SCFA, endocrine hormone, and differential metabolites.

P	Acetic acid	Propanoic acid	Isobutyric acid	Butanoic acid	Isovaleric acid	Valeric acid	Isohexanoic acid	Hexanoic acid	Straight-chain fatty acids	Branched-chain fatty acids	Total SCFA
Ndufaf1	0.025	0.055	0.692	0.075	0.381	0.820	0.012	0.134	0.025	0.465	0.030
Ndufs1	0.008	0.016	0.606	0.021	0.984	0.659	0.175	0.669	0.006	0.839	0.006
Klhl13	0.155	0.493	0.566	0.221	0.632	0.851	0.151	0.114	0.148	0.539	0.148
Prdm16	0.360	0.665	0.262	0.482	0.419	0.670	0.485	0.144	0.353	0.308	0.340
Elovl2	0.377	0.962	0.120	0.384	0.119	0.334	0.033	0.001	0.352	0.094	0.328
Plpp3	0.386	0.629	0.182	0.522	0.218	0.623	0.140	0.075	0.371	0.171	0.351
Lpcat3	0.297	0.001	0.917	0.255	0.654	0.397	0.001	0.001	0.204	0.764	0.216
Pld4	0.008	0.000	0.654	0.073	0.728	0.065	0.028	0.219	0.006	0.786	0.006
TG(18:4/18:2/22:6)	0.268	0.653	0.481	0.274	0.404	0.802	0.009	0.000	0.275	0.369	0.267
TG(20:5/18:2/22:6)	0.046	0.647	0.172	0.135	0.112	0.134	0.006	0.000	0.047	0.108	0.043
DG(18:3/22:6)	0.116	0.932	0.187	0.286	0.117	0.258	0.001	0.000	0.130	0.110	0.120
DG(20:4/22:6)	0.041	0.546	0.102	0.095	0.093	0.104	0.008	0.000	0.038	0.072	0.034
OAHFA(22:6/22:5)	0.280	0.965	0.323	0.411	0.309	0.465	0.004	0.000	0.279	0.248	0.267
FA(22:6)	0.609	0.426	0.357	0.743	0.237	0.583	0.000	0.000	0.638	0.223	0.611
PE(18:2e/22:6)	0.011	0.271	0.135	0.080	0.146	0.115	0.046	0.004	0.011	0.113	0.010
PE(18:1e/22:6)	0.004	0.088	0.282	0.034	0.375	0.209	0.239	0.054	0.003	0.290	0.003
PE(16:1e/22:6)	0.030	0.550	0.164	0.204	0.143	0.190	0.009	0.000	0.037	0.118	0.034
PC(18:1/22:6)	0.006	0.044	0.563	0.063	0.842	0.343	0.567	0.245	0.005	0.653	0.006
PC(16:1/22:6)	0.014	0.448	0.059	0.076	0.033	0.028	0.034	0.003	0.014	0.035	0.012

Continued Table 4：The *P* values of Spearman's correlation analysis among obesity-related biochemical indexes, gut microbiota, SCFA, endocrine hormone, and differential metabolites.

P	g__Blautia	g__Romboutsia	g__Lachnoclostridium	g__Staphylococcus	g__unclassified_f__Oscillospiraceae	g__Ruminococcus	g__Lactobacillus	g__norank_f__Muribaculaceae	g__Lachnospiraceae_NK4A136_group	g__norank_o__Clostridia_UCG-014
Weight	0.055	0.128	0.005	0.040	0.005	0.014	0.011	0.765	0.837	0.012
ALT	0.526	0.249	0.043	0.174	0.613	0.456	0.052	0.742	0.483	0.342
AST	0.161	0.339	0.091	0.297	0.050	0.268	0.060	0.449	0.603	0.061
CHO	0.267	0.976	0.572	0.786	0.066	0.138	0.039	0.366	0.273	0.372
TG	0.002	0.156	0.013	0.043	0.004	0.007	0.059	0.133	0.621	0.049
HDL	0.003	0.034	0.009	0.017	0.009	0.053	0.001	0.094	0.049	0.092
LDL	0.019	0.606	0.081	0.090	0.197	0.130	0.057	0.781	0.231	0.138
GLP-1	0.179	0.455	0.666	0.501	0.851	0.711	0.654	0.522	0.555	0.709
PYY	0.046	0.028	0.012	0.010	0.295	0.030	0.553	0.002	0.157	0.203
CCK	0.251	0.443	0.097	0.088	0.827	0.103	0.362	0.937	0.623	0.015
INS	0.210	0.207	0.222	0.295	0.619	0.733	0.215	0.011	0.606	0.587
GAS	0.891	0.656	0.937	0.851	0.402	0.652	0.770	0.406	0.894	0.749
MTL	0.461	0.830	0.714	0.924	0.004	0.476	0.299	0.899	0.629	0.948
Acetic acid	0.006	0.055	0.037	0.025	0.025	0.022	0.163	0.000	0.000	0.450
Propanoic acid	0.162	0.881	0.675	0.482	0.968	0.250	0.634	0.002	0.005	0.880
Isobutyric acid	0.051	0.560	0.302	0.264	0.554	0.316	0.307	0.401	0.264	0.713
Butanoic acid	0.019	0.174	0.085	0.052	0.554	0.391	0.253	0.021	0.000	0.405
Isovaleric acid	0.041	0.648	0.362	0.342	0.445	0.341	0.148	0.778	0.278	0.779
Valeric acid	0.010	0.785	0.718	0.574	0.480	0.339	0.449	0.271	0.054	0.758
Isohexanoic acid	0.147	0.750	0.136	0.455	0.036	0.188	0.004	0.196	0.186	0.208
Hexanoic acid	0.037	0.188	0.022	0.086	0.006	0.118	0.000	0.906	0.722	0.255
Straight-chainfattyacids	0.004	0.076	0.042	0.026	0.058	0.035	0.183	0.000	0.000	0.432
Branched-chainfatty acids	0.039	0.587	0.288	0.280	0.436	0.293	0.178	0.607	0.316	0.686
TotalSCFA	0.003	0.077	0.040	0.025	0.058	0.034	0.173	0.000	0.000	0.430

Continued Table 4：The *P* values of Spearman's correlation analysis among obesity-related biochemical indexes, gut microbiota, SCFA, endocrine hormone, and differential metabolites.

P	g__Blautia	g__Romboutsia	g__Lachnoclostridium	g__Staphylococcus	g__unclassified_f__Oscillospiraceae	g__Ruminococcus	g__Lactobacillus	g__norank_f__Muribaculaceae	g__Lachnospiraceae_NK4A136_group	g__norank_o__Clostridia_UCG-014
g__Blautia		0.148	0.018	0.025	0.015	0.005	0.002	0.023	0.137	0.057
g__Romboutsia	0.148		0.000	0.000	0.061	0.013	0.030	0.018	0.098	0.315
g__Lachnoclostridium	0.018	0.000		0.000	0.015	0.004	0.007	0.030	0.260	0.040
g__Staphylococcus	0.025	0.000	0.000		0.038	0.013	0.025	0.032	0.163	0.127
g__unclassified_f__Oscillospiraceae	0.015	0.061	0.015	0.038		0.032	0.018	0.246	0.554	0.133
g__Ruminococcus	0.005	0.013	0.004	0.013	0.032		0.012	0.035	0.510	0.097
g__Lactobacillus	0.002	0.030	0.007	0.025	0.018	0.012		0.508	0.524	0.378
g__norank_f__Muribaculaceae	0.023	0.018	0.030	0.032	0.246	0.035	0.508		0.014	0.201
g__Lachnospiraceae_NK4A136_group	0.137	0.098	0.260	0.163	0.554	0.510	0.524	0.014		0.752
g__norank_o__Clostridia_UCG-014	0.057	0.315	0.040	0.127	0.133	0.097	0.378	0.201	0.752	
Ehhadh	0.527	0.202	0.317	0.220	0.881	0.262	0.274	0.012	0.069	0.085
Acox1	0.934	0.093	0.323	0.075	0.656	0.893	0.706	0.151	0.034	0.628
Acsl1	0.473	0.117	0.425	0.140	0.767	0.775	0.594	0.007	0.003	0.509
Fabp4	0.869	0.438	0.787	0.696	0.467	0.392	0.843	0.163	0.312	0.755
Lpl	0.163	0.086	0.083	0.038	0.913	0.494	0.718	0.011	0.046	0.024
Pparg	0.786	0.143	0.581	0.158	0.671	0.779	0.326	0.159	0.090	0.718
Ndufaf1	0.536	0.076	0.306	0.071	0.751	0.643	0.808	0.017	0.008	0.562
Ndufs1	0.196	0.051	0.059	0.020	0.609	0.243	0.595	0.006	0.007	0.116
Klhl13	0.023	0.031	0.004	0.014	0.216	0.000	0.004	0.121	0.524	0.013
Prdm16	0.378	0.067	0.075	0.156	0.156	0.049	0.104	0.394	0.431	0.144
Elovl2	0.382	0.185	0.037	0.096	0.025	0.207	0.134	0.481	0.975	0.046

Continued Table 4：The *P* values of Spearman's correlation analysis among obesity-related biochemical indexes, gut microbiota, SCFA, endocrine hormone, and differential metabolites.

P	g__Blautia	g__Romboutsia	g__Lachnoclostridium	g__Staphylococcus	g__unclassified_f__Oscillospiraceae	g__Ruminococcus	g__Lactobacillus	g__norank_f__Muribaculaceae	g__Lachnospiraceae_NK4A136_group	g__norank_o__Clostridia_UCG-014
Plpp3	0.120	0.047	0.034	0.082	0.432	0.000	0.014	0.263	0.954	0.118
Lpcat3	0.753	0.294	0.158	0.424	0.035	0.988	0.029	0.194	0.145	0.641
Pld4	0.356	0.924	0.750	0.837	0.629	0.398	0.642	0.014	0.002	0.865
TG(18:4/18:2/22:6)	0.040	0.006	0.001	0.006	0.004	0.003	0.000	0.297	0.791	0.021
TG(20:5/18:2/22:6)	0.001	0.033	0.001	0.007	0.001	0.001	0.000	0.157	0.464	0.011
DG(18:3/22:6)	0.004	0.034	0.002	0.021	0.000	0.001	0.000	0.319	0.619	0.021
DG(20:4/22:6)	0.001	0.040	0.001	0.011	0.001	0.001	0.000	0.173	0.393	0.017
OAHFA(22:6/22:5)	0.028	0.098	0.007	0.046	0.005	0.004	0.001	0.316	0.863	0.020
FA(22:6)	0.053	0.176	0.015	0.095	0.009	0.015	0.000	0.845	0.524	0.034
PE(18:2e/22:6)	0.000	0.022	0.001	0.004	0.004	0.000	0.000	0.018	0.315	0.011
PE(18:1e/22:6)	0.000	0.014	0.001	0.004	0.015	0.000	0.005	0.003	0.118	0.005
PE(16:1e/22:6)	0.000	0.029	0.001	0.006	0.001	0.000	0.000	0.090	0.583	0.017
PC(18:1/22:6)	0.001	0.066	0.005	0.016	0.053	0.000	0.027	0.002	0.220	0.025
PC(16:1/22:6)	0.000	0.050	0.005	0.013	0.002	0.000	0.000	0.088	0.219	0.018

Continued Table 4：The *P* values of Spearman's correlation analysis among obesity-related biochemical indexes, gut microbiota, SCFA, endocrine hormone, and differential metabolites.

P	Ehhadh	Acox1	Acsl1	Fabp4	Lpl	Pparg	Ndufaf1	Ndufs1	Klhl13	Prdm16	Elovl2	Plpp3	Lpcat3	Pld4
Weight	0.444	0.862	0.540	0.491	0.538	0.409	0.786	0.412	0.001	0.106	0.067	0.010	0.045	0.349
ALT	0.045	0.756	0.500	0.970	0.680	0.280	0.416	0.971	0.173	0.156	0.021	0.228	0.157	0.045
AST	0.124	0.575	0.688	0.714	0.494	0.239	0.732	0.893	0.267	0.181	0.025	0.614	0.137	0.142
CHO	0.266	0.068	0.012	0.308	0.063	0.018	0.124	0.215	0.183	0.425	0.031	0.044	0.069	0.199
TG	0.243	0.385	0.717	0.189	0.736	0.168	0.644	0.915	0.059	0.532	0.064	0.118	0.220	0.917
HDL	0.555	0.464	0.494	0.252	0.216	0.592	0.175	0.028	0.011	0.022	0.291	0.087	0.326	0.407
LDL	0.680	0.428	0.365	0.208	0.798	0.092	0.377	0.609	0.112	0.693	0.092	0.202	0.211	0.098
GLP-1	0.344	0.851	0.598	0.499	0.656	0.795	0.729	0.823	0.934	0.513	0.212	0.927	0.501	0.231
PYY	0.025	0.351	0.107	0.176	0.057	0.097	0.207	0.035	0.217	0.824	0.752	0.335	0.588	0.031
CCK	0.654	0.499	0.907	0.739	0.474	0.895	0.938	0.442	0.017	0.167	0.038	0.004	0.944	0.292
INS	0.517	0.954	0.543	0.644	0.484	0.657	0.844	0.562	0.894	0.859	0.315	0.715	0.720	0.678
GAS	0.418	0.265	0.755	0.269	0.858	0.205	0.649	0.541	0.275	0.230	0.012	0.199	0.876	0.780
MTL	0.262	0.043	0.150	0.023	0.171	0.067	0.282	0.120	0.469	0.342	0.085	0.466	0.081	0.566
Acetic acid	0.106	0.235	0.056	0.340	0.158	0.214	0.025	0.008	0.155	0.360	0.377	0.386	0.297	0.008
Propanoic acid	0.036	0.165	0.027	0.060	0.156	0.150	0.055	0.016	0.493	0.665	0.962	0.629	0.001	0.000
Isobutyric acid	0.690	0.867	0.727	0.442	0.796	0.699	0.692	0.606	0.566	0.262	0.120	0.182	0.917	0.654
Butanoic acid	0.146	0.069	0.020	0.708	0.029	0.350	0.075	0.021	0.221	0.482	0.384	0.522	0.255	0.073
Isovaleric acid	0.436	0.684	0.804	0.870	0.770	0.826	0.381	0.984	0.632	0.419	0.119	0.218	0.654	0.728
Valeric acid	0.871	0.795	0.687	0.602	0.889	0.890	0.820	0.659	0.851	0.670	0.334	0.623	0.397	0.065
Isohexanoic acid	0.063	0.038	0.008	0.060	0.244	0.007	0.012	0.175	0.151	0.485	0.033	0.140	0.001	0.028
Hexanoic acid	0.106	0.191	0.144	0.643	0.763	0.105	0.134	0.669	0.114	0.144	0.001	0.075	0.001	0.219
Straight-chainfattyacids	0.088	0.176	0.034	0.317	0.103	0.214	0.025	0.006	0.148	0.353	0.352	0.371	0.204	0.006
Branched-chainfattyacids	0.509	0.847	0.959	0.694	0.965	0.984	0.465	0.839	0.539	0.308	0.094	0.171	0.764	0.786
TotalSCFA	0.099	0.185	0.038	0.316	0.109	0.221	0.030	0.006	0.148	0.340	0.328	0.351	0.216	0.006
g__Blautia	0.527	0.934	0.473	0.869	0.163	0.786	0.536	0.196	0.023	0.378	0.382	0.120	0.753	0.356
g__Romboutsia	0.202	0.093	0.117	0.438	0.086	0.143	0.076	0.051	0.031	0.067	0.185	0.047	0.294	0.924
g__Lachnoclostridium	0.317	0.323	0.425	0.787	0.083	0.581	0.306	0.059	0.004	0.075	0.037	0.034	0.158	0.750

Continued Table 4：The *P* values of Spearman's correlation analysis among obesity-related biochemical indexes, gut microbiota, SCFA, endocrine hormone, and differential metabolites.

P	Ehhadh	Acox1	Acsl1	Fabp4	Lpl	Pparg	Ndufaf1	Ndufs1	Klhl13	Prdm16	Elovl2	Plpp3	Lpcat3	Pld4
g__Staphylococcus	0.220	0.075	0.140	0.696	0.038	0.158	0.071	0.020	0.014	0.156	0.096	0.082	0.424	0.837
g__unclassified_f__Oscillospiraceae	0.881	0.656	0.767	0.467	0.913	0.671	0.751	0.609	0.216	0.156	0.025	0.432	0.035	0.629
g__Ruminococcus	0.262	0.893	0.775	0.392	0.494	0.779	0.643	0.243	0.000	0.049	0.207	0.000	0.988	0.398
g__Lactobacillus	0.274	0.706	0.594	0.843	0.718	0.326	0.808	0.595	0.004	0.104	0.134	0.014	0.029	0.642
g__norank_f__Muribaculaceae	0.012	0.151	0.007	0.163	0.011	0.159	0.017	0.006	0.121	0.394	0.481	0.263	0.194	0.014
g__Lachnospiraceae_NK4A136_group	0.069	0.034	0.003	0.312	0.046	0.090	0.008	0.007	0.524	0.431	0.975	0.954	0.145	0.002
g__norank_o__Clostridia_UCG-014	0.085	0.628	0.509	0.755	0.024	0.718	0.562	0.116	0.013	0.144	0.046	0.118	0.641	0.865
Ehhadh		0.031	0.005	0.283	0.011	0.053	0.008	0.009	0.203	0.685	0.508	0.491	0.071	0.019
Acox1	0.031		0.000	0.114	0.000	0.000	0.000	0.000	0.179	0.327	0.508	0.577	0.033	0.524
Acsl1	0.005	0.000		0.156	0.000	0.000	0.000	0.000	0.325	0.704	0.429	0.932	0.009	0.051
Fabp4	0.283	0.114	0.156		0.273	0.071	0.204	0.060	0.319	0.003	0.874	0.083	0.124	0.076
Lpl	0.011	0.000	0.000	0.273		0.007	0.000	0.000	0.032	0.511	0.980	0.499	0.164	0.228
Pparg	0.053	0.000	0.000	0.071	0.007		0.000	0.000	0.785	0.703	0.256	0.929	0.057	0.378
Ndufaf1	0.008	0.000	0.000	0.204	0.000	0.000		0.000	0.211	0.518	0.485	0.851	0.027	0.181
Ndufs1	0.009	0.000	0.000	0.060	0.000	0.000	0.000		0.012	0.181	0.992	0.225	0.067	0.112
Klhl13	0.203	0.179	0.325	0.319	0.032	0.785	0.211	0.012		0.022	0.322	0.000	0.945	0.814
Prdm16	0.685	0.327	0.704	0.003	0.511	0.703	0.518	0.181	0.022		0.098	0.004	0.614	0.894
Elovl2	0.508	0.508	0.429	0.874	0.980	0.256	0.485	0.992	0.322	0.098		0.150	0.095	0.433
Plpp3	0.491	0.577	0.932	0.083	0.499	0.929	0.851	0.225	0.000	0.004	0.150		0.841	0.898
Lpcat3	0.071	0.033	0.009	0.124	0.164	0.057	0.027	0.067	0.945	0.614	0.095	0.841		0.013
Pld4	0.019	0.524	0.051	0.076	0.228	0.378	0.181	0.112	0.814	0.894	0.433	0.898	0.013	
TG(18:4/18:2/22:6)	0.680	0.945	0.866	0.878	0.358	0.482	0.965	0.393	0.001	0.029	0.002	0.001	0.036	0.609
TG(20:5/18:2/22:6)	0.435	0.704	0.845	0.973	0.360	0.391	0.903	0.332	0.001	0.080	0.016	0.008	0.080	0.701
DG(18:3/22:6)	0.928	0.485	0.503	0.861	0.634	0.195	0.585	0.562	0.002	0.015	0.005	0.006	0.022	0.893
DG(20:4/22:6)	0.773	0.694	0.741	0.591	0.469	0.384	0.736	0.386	0.006	0.014	0.013	0.014	0.108	0.813
OAHFA(22:6/22:5)	0.913	0.581	0.594	0.842	0.430	0.274	0.585	0.505	0.002	0.079	0.003	0.005	0.058	0.830
FA(22:6)	0.560	0.240	0.187	0.791	0.873	0.095	0.195	0.979	0.009	0.151	0.003	0.012	0.007	0.472

Continued Table 4：The *P* values of Spearman's correlation analysis among obesity-related biochemical indexes, gut microbiota, SCFA, endocrine hormone, and differential metabolites.

P	Ehhadh	Acox1	Acsl1	Fabp4	Lpl	Pparg	Ndufaf1	Ndufs1	Klhl13	Prdm16	Elovl2	Plpp3	Lpcat3	Pld4
PE(18:2e/22:6)	0.375	0.838	0.561	0.657	0.093	0.757	0.589	0.087	0.000	0.078	0.081	0.003	0.448	0.453
PE(18:1e/22:6)	0.059	0.363	0.209	0.303	0.023	0.847	0.190	0.010	0.000	0.024	0.165	0.002	0.928	0.210
PE(16:1e/22:6)	0.655	0.908	0.993	0.746	0.249	0.574	0.881	0.180	0.000	0.054	0.041	0.002	0.173	0.807
PC(18:1/22:6)	0.154	0.324	0.185	0.308	0.034	0.869	0.138	0.018	0.000	0.054	0.359	0.016	0.470	0.305
PC(16:1/22:6)	0.605	0.822	0.852	0.816	0.301	0.596	0.914	0.310	0.009	0.117	0.093	0.023	0.272	0.414

Continued Table 4：The *P* values of Spearman's correlation analysis among obesity-related biochemical indexes, gut microbiota, SCFA, endocrine hormone, and differential metabolites.

P	TG(18:4/18:2/22:6)	TG(20:5/18:2/22:6)	DG(18:3/22:6)	DG(20:4/22:6)	OAHFA(22:6/22:5)	FA(22:6)	PE(18:2e/22:6)	PE(18:1e/22:6)	PE(16:1e/22:6)	PC(18:1/22:6)	PC(16:1/22:6)
Weight	0.000	0.000	0.000	0.001	0.000	0.000	0.001	0.001	0.000	0.048	0.008
ALT	0.133	0.382	0.085	0.140	0.106	0.080	0.206	0.358	0.174	0.204	0.519
AST	0.117	0.199	0.031	0.061	0.067	0.063	0.067	0.179	0.065	0.056	0.146
CHO	0.025	0.040	0.028	0.131	0.051	0.016	0.192	0.367	0.061	0.718	0.107
TG	0.005	0.000	0.001	0.002	0.004	0.009	0.001	0.002	0.001	0.028	0.002
HDL	0.033	0.004	0.004	0.004	0.061	0.080	0.003	0.001	0.002	0.020	0.001
LDL	0.028	0.022	0.039	0.049	0.023	0.025	0.025	0.098	0.023	0.073	0.047
GLP-1	0.418	0.663	0.762	0.406	0.942	0.775	0.430	0.397	0.628	0.474	0.329
PYY	0.291	0.062	0.252	0.051	0.175	0.352	0.016	0.015	0.042	0.032	0.025
CCK	0.055	0.099	0.150	0.146	0.115	0.102	0.129	0.139	0.102	0.290	0.140
INS	0.496	0.593	0.571	0.462	0.340	0.506	0.251	0.411	0.335	0.359	0.437
GAS	0.691	0.835	0.792	0.961	0.435	0.536	0.976	0.761	0.964	0.994	0.861
MTL	0.155	0.194	0.162	0.241	0.116	0.095	0.380	0.853	0.280	0.743	0.275
Acetic acid	0.268	0.046	0.116	0.041	0.280	0.609	0.011	0.004	0.030	0.006	0.014
Propanoic acid	0.653	0.647	0.932	0.546	0.965	0.426	0.271	0.088	0.550	0.044	0.448
Isobutyric acid	0.481	0.172	0.187	0.102	0.323	0.357	0.135	0.282	0.164	0.563	0.059
Butanoic acid	0.274	0.135	0.286	0.095	0.411	0.743	0.080	0.034	0.204	0.063	0.076

Continued Table 4：The *P* values of Spearman's correlation analysis among obesity-related biochemical indexes, gut microbiota, SCFA, endocrine hormone, and differential metabolites.

P	TG(18:4/18:2/22:6)	TG(20:5/18:2/22:6)	DG(18:3/22:6)	DG(20:4/22:6)	OAHFA(22:6/22:5)	FA(22:6)	PE(18:2e/22:6)	PE(18:1e/22:6)	PE(16:1e/22:6)	PC(18:1/22:6)	PC(16:1/22:6)
Isovaleric acid	0.404	0.112	0.117	0.093	0.309	0.237	0.146	0.375	0.143	0.842	0.033
Valeric acid	0.802	0.134	0.258	0.104	0.465	0.583	0.115	0.209	0.190	0.343	0.028
Isohexanoic acid	0.009	0.006	0.001	0.008	0.004	0.000	0.046	0.239	0.009	0.567	0.034
Hexanoic acid	0.000	0.000	0.000	0.000	0.000	0.000	0.004	0.054	0.000	0.245	0.003
Straight-chainfattyacids	0.275	0.047	0.130	0.038	0.279	0.638	0.011	0.003	0.037	0.005	0.014
Branched-chainfattyacids	0.369	0.108	0.110	0.072	0.248	0.223	0.113	0.290	0.118	0.653	0.035
TotalSCFA	0.267	0.043	0.120	0.034	0.267	0.611	0.010	0.003	0.034	0.006	0.012
g__Blautia	0.040	0.001	0.004	0.001	0.028	0.053	0.000	0.000	0.000	0.001	0.000
g__Romboutsia	0.006	0.033	0.034	0.040	0.098	0.176	0.022	0.014	0.029	0.066	0.050
g__Lachnoclostridium	0.001	0.001	0.002	0.001	0.007	0.015	0.001	0.001	0.001	0.005	0.005
g__Staphylococcus	0.006	0.007	0.021	0.011	0.046	0.095	0.004	0.004	0.006	0.016	0.013
g__unclassified_f__Oscillospiraceae	0.004	0.001	0.000	0.001	0.005	0.009	0.004	0.015	0.001	0.053	0.002
g__Ruminococcus	0.003	0.001	0.001	0.001	0.004	0.015	0.000	0.000	0.000	0.000	0.000
g__Lactobacillus	0.000	0.000	0.000	0.000	0.001	0.000	0.000	0.005	0.000	0.027	0.000
g__norank_f__Muribaculaceae	0.297	0.157	0.319	0.173	0.316	0.845	0.018	0.003	0.090	0.002	0.088
g__Lachnospiraceae_NK4A136_group	0.791	0.464	0.619	0.393	0.863	0.524	0.315	0.118	0.583	0.220	0.219
g__norank_o__Clostridia_UCG-014	0.021	0.011	0.021	0.017	0.020	0.034	0.011	0.005	0.017	0.025	0.018
Ehhadh	0.680	0.435	0.928	0.773	0.913	0.560	0.375	0.059	0.655	0.154	0.605
Acox1	0.945	0.704	0.485	0.694	0.581	0.240	0.838	0.363	0.908	0.324	0.822
Acsl1	0.866	0.845	0.503	0.741	0.594	0.187	0.561	0.209	0.993	0.185	0.852
Fabp4	0.878	0.973	0.861	0.591	0.842	0.791	0.657	0.303	0.746	0.308	0.816
Lpl	0.358	0.360	0.634	0.469	0.430	0.873	0.093	0.023	0.249	0.034	0.301
Pparg	0.482	0.391	0.195	0.384	0.274	0.095	0.757	0.847	0.574	0.869	0.596
Ndufaf1	0.965	0.903	0.585	0.736	0.585	0.195	0.589	0.190	0.881	0.138	0.914
Ndufs1	0.393	0.332	0.562	0.386	0.505	0.979	0.087	0.010	0.180	0.018	0.310
Klhl13	0.001	0.001	0.002	0.006	0.002	0.009	0.000	0.000	0.000	0.000	0.009

Continued Table 4：The *P* values of Spearman's correlation analysis among obesity-related biochemical indexes, gut microbiota, SCFA, endocrine hormone, and differential metabolites.

P	TG(18:4/18:2/22:6)	TG(20:5/18:2/22:6)	DG(18:3/22:6)	DG(20:4/22:6)	OAHFA(22:6/22:5)	FA(22:6)	PE(18:2e/22:6)	PE(18:1e/22:6)	PE(16:1e/22:6)	PC(18:1/22:6)	PC(16:1/22:6)
Prdm16	0.029	0.080	0.015	0.014	0.079	0.151	0.078	0.024	0.054	0.054	0.117
Elovl2	0.002	0.016	0.005	0.013	0.003	0.003	0.081	0.165	0.041	0.359	0.093
Plpp3	0.001	0.008	0.006	0.014	0.005	0.012	0.003	0.002	0.002	0.016	0.023
Lpcat3	0.036	0.080	0.022	0.108	0.058	0.007	0.448	0.928	0.173	0.470	0.272
Pld4	0.609	0.701	0.893	0.813	0.830	0.472	0.453	0.210	0.807	0.305	0.414
TG(18:4/18:2/22:6)		0.000	0.000	0.000	0.000	0.000	0.000	0.001	0.000	0.022	0.001
TG(20:5/18:2/22:6)	0.000		0.000	0.000	0.000	0.000	0.000	0.000	0.000	0.004	0.000
DG(18:3/22:6)	0.000	0.000		0.000	0.000	0.000	0.000	0.000	0.000	0.007	0.000
DG(20:4/22:6)	0.000	0.000	0.000		0.000	0.000	0.000	0.000	0.000	0.001	0.000
OAHFA(22:6/22:5)	0.000	0.000	0.000	0.000		0.000	0.000	0.000	0.000	0.012	0.001
FA(22:6)	0.000	0.000	0.000	0.000	0.000		0.000	0.006	0.000	0.075	0.001
PE(18:2e/22:6)	0.000	0.000	0.000	0.000	0.000	0.000		0.000	0.000	0.000	0.000
PE(18:1e/22:6)	0.001	0.000	0.000	0.000	0.000	0.006	0.000		0.000	0.000	0.000
PE(16:1e/22:6)	0.000	0.000	0.000	0.000	0.000	0.000	0.000	0.000		0.000	0.000
PC(18:1/22:6)	0.022	0.004	0.007	0.001	0.012	0.075	0.000	0.000	0.000		0.002
PC(16:1/22:6)	0.001	0.000	0.000	0.000	0.001	0.001	0.000	0.000	0.000	0.002	
