# Supplementary material for: Betaine alleviates obesity-related metabolic disorders in rats: insights from microbiomes, lipidomics, and transcriptomics
Source: Front Nutr. 2025 Jul 10;12:1604801. doi: 10.3389/fnut.2025.1604801 (PMC12286965; doi:10.3389/fnut.2025.1604801)
Supplement: Supplementary file 2 [file Image_1.pdf]

Supplementary Material

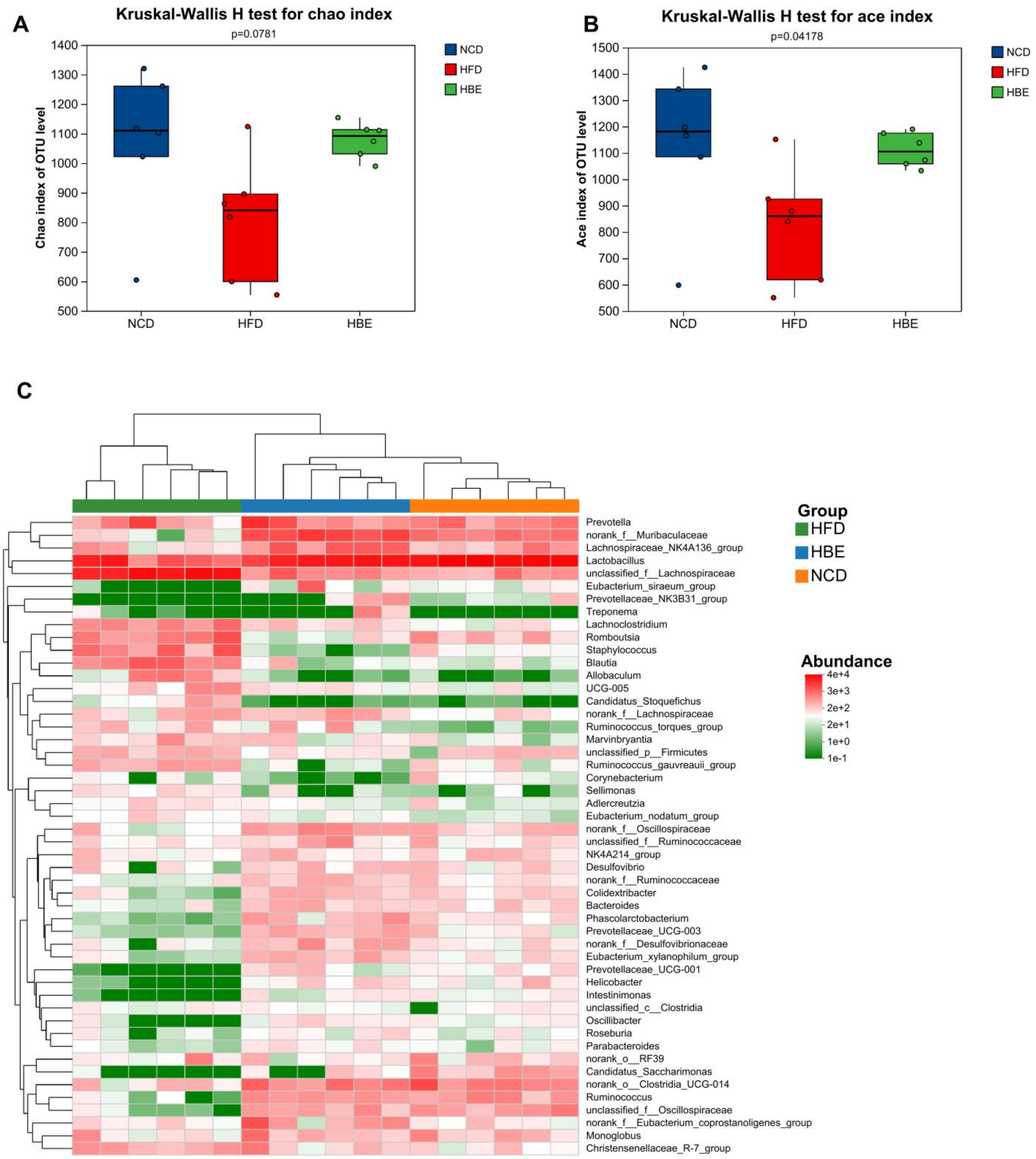

Supplementary Figure 1. Betaine influences gut microbiota in HFD-fed rats. (A) Kruskal-Wallis H test for chao index; (B) Kruskal-Wallis H test for ace index; (C) Community heatmap analysis on genus level. Data are shown as means ± SD (n=6).

.

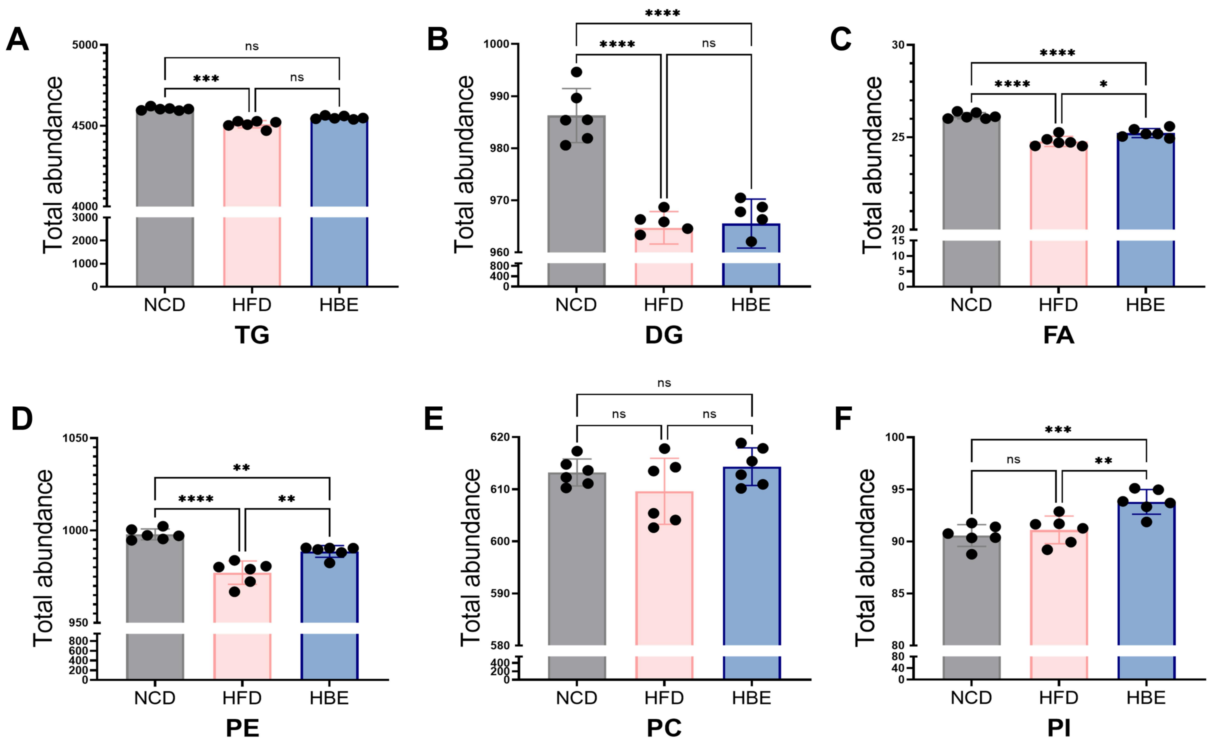

Supplementary Figure 2. Betaine alters glycerolipid, glycerophospholipid and fatty acids metabolism of adipose tissues. Lipidomic analysis of adipose tissues was performed by LC-MS/MS. Changes of overall abundance of (A) Triglyceride (TG), (B) Diglyceride (DG), (C) Phosphatidylethanolamine (PE), (D) Phosphatidylcholine (PC), (E) Phosphatidylinositol (PI), and (F) fatty acids (FA) in the adipose tissues were detected (n = 6/group). Data were expressed as mean ± SD of (n = 6). *P<0.05, **P<0.01,***P<0.001, ****P<0.0001. ns, not significant.

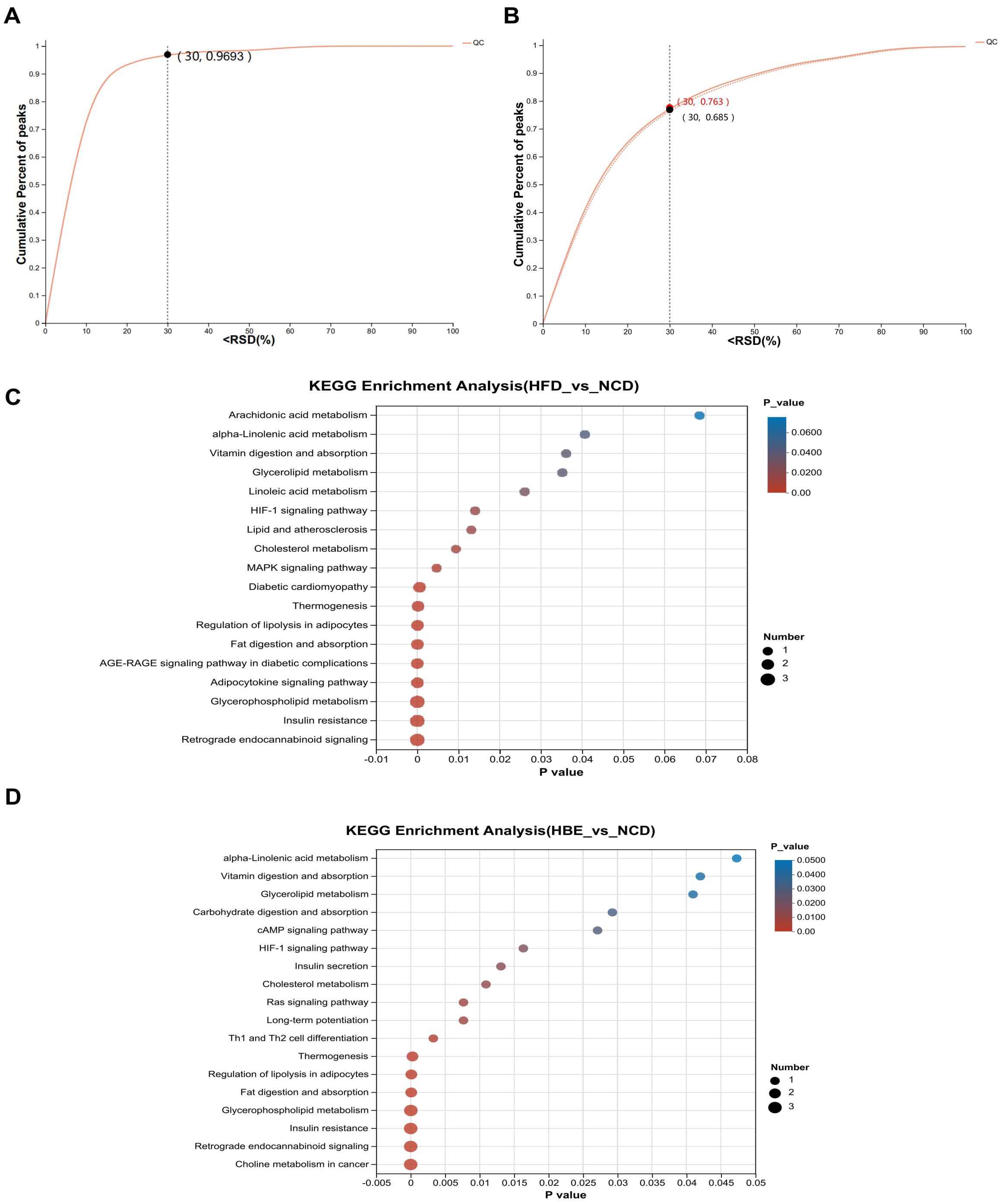

Supplementary Figure 3. Lipidomics analysis of the adipose tissues. QC samples with relative standard deviation (RSD) distribution plot of (A) ESI+ions and (B) ESI-ions (The dashed line indicates before the preprocessing, and the solid line indicates after the preprocessing); Pathway enrichments by Kyoto Encyclopedia of Genes and Genomes (KEGG) analyses between (C) HFD vs. NCD and （D）HBE vs. NCD.

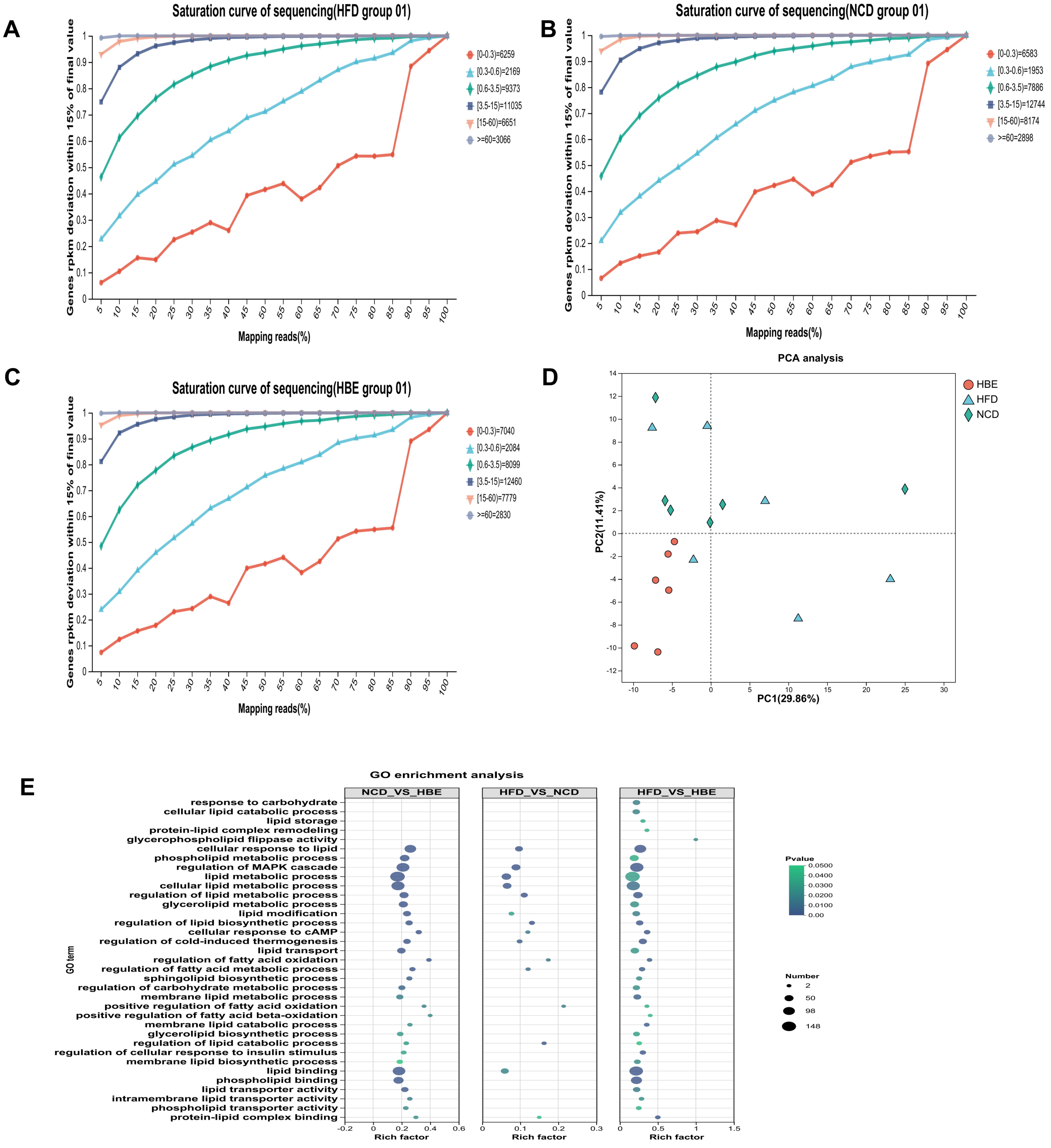

Supplementary Figure 4. Transcriptomic analysis of the adipose tissues. Saturation curve of sequencing (A)HFD group sample ; (B)NCD group sample; (C)HBE group sample; (D) Principal component analysis; (E)Pathway enrichments by Genomes (GO) analyses between NCD vs. HBE, HFD vs. NCD, and HBE vs. NCD.
